# Charged Thienobenzo-1,2,3-Triazoles as Especially Potent Non-Selective Cholinesterase Inhibitors: Design, Anti-Inflammatory Activity, and Computational Study

**DOI:** 10.3390/ph18071032

**Published:** 2025-07-11

**Authors:** Antonija Jelčić, Anamarija Raspudić, Danijela Barić, Ana Ratković, Ivana Šagud, Paula Pongrac, Dora Štefok, Martina Bosnar, Sunčica Roca, Zlata Lasić, Ilijana Odak, Irena Škorić

**Affiliations:** 1 Department of Organic Chemistry, Faculty of Chemical Engineering and Technology, University of Zagreb, Trg Marka Marulića 19, HR-10 000 Zagreb, Croatia; ajelcic@fkit.unizg.hr (A.J.); ana.ratkovic30@gmail.com (A.R.); 2 Department of Chemistry, Faculty of Science and Education, University of Mostar, Matice Hrvatske bb, 88 000 Mostar, Bosnia and Herzegovina; anamarija.raspudic@fpmoz.sum.ba; 3 Group for Computational Life Sciences, Division of Physical Chemistry, Ruđer Bošković Institute, Bijenička Cesta 54, HR-10 000 Zagreb, Croatia; dbaric@irb.hr; 4 Croatian Agency for Medicinal Products and Medical Devices, Ksaverska Cesta 4, HR-10 000 Zagreb, Croatia; ivana.sagud@halmed.hr; 5 Pharmacology In Vitro, Selvita Ltd., Prilaz Baruna Filipovića 29, HR-10 000 Zagreb, Croatia; paula.pongrac@selvita.com (P.P.); dora.stefok@selvita.com (D.Š.); martina.bosnar@selvita.com (M.B.); 6 NMR Center, Ruđer Bošković Institute, Bijenička Cesta 54, HR-10 000 Zagreb, Croatia; sroca@irb.hr; 7 TEVA Global R&D, E&L R&D, Pliva Hrvatska d.o.o., Prilaz Baruna Filipovića 25, HR-10 000 Zagreb, Croatia; zlata.lasic01@pliva.com

**Keywords:** thienobenzo-1,2,3-triazoles, cholinesterase, BChE inhibitors, anti-inflammatory activity, molecular docking, ADME-Tox prediction

## Abstract

**Background/Objectives**: This research reports the synthesis and evaluation of novel charged thienobenzo-triazoles as non-selective cholinesterase inhibitors (AChEs and BChEs), their anti-inflammatory properties, and a computational study. **Methods**: Fifteen derivatives were created through photochemical cyclization and quaternization of the triazole core. The compounds were tested for AChE and BChE inhibition. They showed greater potency and selectivity toward BChE. **Results**: The most potent compound, derivative 14, inhibited BChE with an IC_50_ of 98 nM, while derivative **9** also displayed significant anti-inflammatory activity by inhibiting LPS-induced TNF-α production (IC_50_ = 0.66 µM). Molecular docking revealed that triazolinium salts form key π-π and electrostatic interactions within enzyme active sites. In silico predictions indicated favorable ADME-Tox properties for compounds **9** and **11**, including low mutagenicity and moderate CNS permeability. **Conclusions**: These findings highlight the potential of new charged triazolinium salts as peripherally selective cholinesterase inhibitors with additional anti-inflammatory potential.

## 1. Introduction

Cholinesterases are essential enzymes that hydrolyze acetylcholine (ACh) and butyrylcholine (BCh), ending cholinergic signaling. The main types are acetylcholinesterase (AChE), mainly located in synaptic clefts, and butyrylcholinesterase (BChE), which is more diffused in plasma and non-neuronal tissues [1]. Pharmacological blocking of these enzymes has therapeutic uses, especially in treating Alzheimer’s disease (AD) and acute organophosphate poisoning [2]. Non-selective cholinesterase inhibitors (ChEIs) affect both AChE and BChE, making them a flexible group of compounds with increasing clinical and research interest. A growing area of focus is the impact of molecular charge, particularly the benefits of positively charged (cationic) molecules.

ChEIs work by preventing the breakdown of ACh, thereby boosting cholinergic signaling. This approach is used in treating symptoms of neurodegenerative diseases like AD, where cholinergic deficits are common [3,4]. Non-selective inhibitors, which target both AChE and BChE, may offer broader benefits. In the later stages of AD, BChE activity rises and may help compensate for the reduced activity of AChE, underscoring the need to target both enzymes [5,6]. Approved ChEIs such as donepezil, galantamine, and rivastigmine have shown effectiveness in the brain, while peripheral inhibitors like neostigmine are important in anesthesia and neuromuscular disorders [7,8].

Non-selective cholinesterase inhibitors include both traditional compounds like physostigmine and neostigmine, as well as newer experimental agents that have reversible and irreversible qualities. Many of these molecules are designed with quaternary ammonium groups, which give them a permanent positive charge. This positive charge makes the molecules hydrophilic and greatly limits their ability to cross the blood–brain barrier (BBB), leading to mainly peripheral effects [9]. Charged ChEIs, especially those with quaternary ammonium groups, provide several pharmacological and safety benefits in clinical use. Because of their polar nature, these molecules poorly penetrate the BBB, which is beneficial in clinical situations where avoiding central nervous system (CNS) side effects is important [10,11].

Furthermore, the limited CNS distribution of charged molecules increases their suitability for peripheral therapeutic targets. This is especially advantageous in conditions such as myasthenia gravis or in reversing neuromuscular blockade after surgery, where a peripheral mode of action is preferred [8,12]. Their restricted distribution in the CNS also helps reduce the risk of neurotoxicity and convulsions, making them safer for use in acute or emergency situations [13,14]. Additionally, their hydrophilic nature results in predictable pharmacokinetics, with distribution mainly confined to extracellular fluids, which allows for consistent plasma levels and simpler dosing adjustments [15]. Despite these benefits, charged ChEIs have some limitations. Their inability to cross the BBB renders them ineffective for treating CNS-related disorders like AD unless delivered through alternative methods or chemically modified to improve CNS penetration [16]. Several charged non-selective ChEIs are currently used in clinical practice [17]. These drugs demonstrate how charged structures can offer targeted therapeutic effects while minimizing central toxicity.

Recent advances in medicinal chemistry aim to overcome the limitations of charged molecules while retaining their advantages. One approach involves designing hybrid or prodrug forms that temporarily mask the charge to facilitate BBB penetration, with activation occurring at the target site [16]. Research also continues into dual-action inhibitors that combine ChE inhibition with anti-oxidant or anti-amyloid properties, which could provide multifunctional benefits in neurodegenerative diseases [5,11]. Efforts are also ongoing to develop tissue-specific delivery systems and nanocarriers that allow controlled distribution of polar ChEIs [18]. Non-selective cholinesterase inhibitors with charged structures offer considerable therapeutic advantages, particularly in the peripheral nervous system, where CNS penetration is undesirable. Their hydrophilic and cationic nature allows for targeted, predictable, and relatively safe pharmacological profiles. However, their limited CNS access remains a significant obstacle in treating brain-related disorders. Ongoing research into prodrug strategies and hybrid molecules holds promise for extending the utility of charged ChEIs in broader clinical contexts.

**Figure 1:**
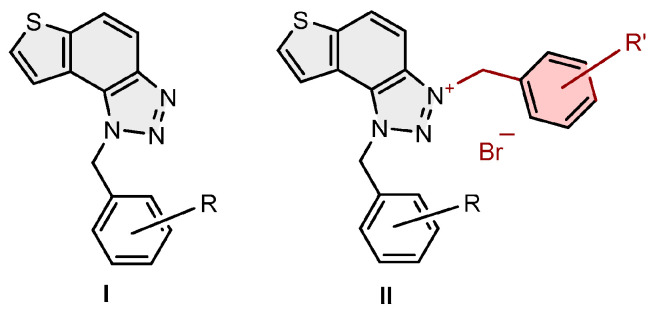
Structures of uncharged thienobenzo-triazoles **I** with preferred inhibition of BChE and their charged triazolinium salts **II** as dual cholinesterase inhibitors [19].

In our previous research [19], pairs of uncharged thienobenzo-triazoles and their corresponding charged salts were prepared to examine the role of the positive charge on the nitrogen of the triazole ring in interactions within the active site of cholinesterase enzymes. The goal was also to compare the selectivity of 1,2,3-triazolinium salts with that of their uncharged analogs. Uncharged thienobenzo-triazoles I (Figure 1) favored inhibition of BChE over AChE. Conversion to the salt form (structure II, Figure 1) significantly increased the inhibition of AChE (the most active IC_50_ 4.4 μM) and also, to a reasonable degree, that of BChE (the most active IC_50_ 0.47 μM). It was concluded that the key structural feature for thienobenzo-triazoles is the presence or absence of charge. Triazolinium salts [19] were shown to be dual inhibitors, with their potency depending on the substituents on the thiophene and triazole heterocycles.

## 2. Results and Discussion

### 2.1. Synthesis of Charged Thienobenzo-1,2,3-Triazoles ***1**–**15***

**Scheme 1:**
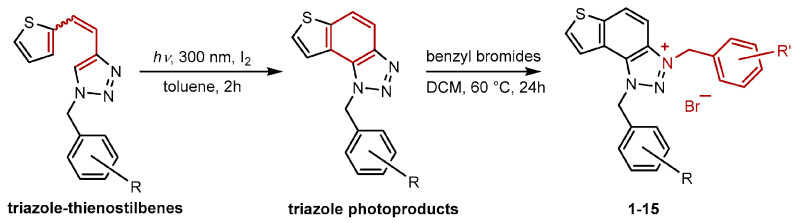
Synthetic steps to charged triazolinium benzyl salts **1**–**15**.

**Figure 2:**
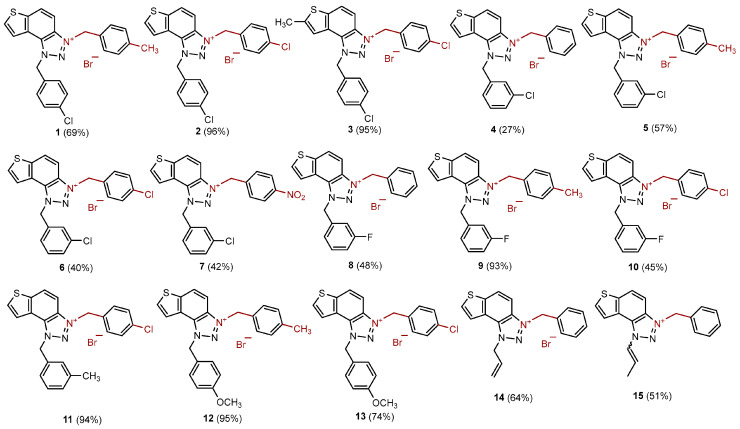
Structures of new charged thienobenzo-1,2,3-triazolinium salts **1**–**15** and their isolated yields in brackets.

New charged thienobenzo-1,2,3-triazoles **1**–**15** were synthesized to explore their potential as AChE and BChE inhibitors, building on previous positive results [19]. To accomplish this, targeted triazole-thienostilbenes (Scheme 1) were prepared through a sequence of consecutive reactions [19]. Mixtures of geometrical isomers of triazole-thienostilbenes were subjected to photochemical cyclization, producing triazole photoproducts that served as starting materials for synthesizing charged triazole benzyl salts **1**–**15**. These photoproducts were then converted into triazolinium salts **1**–**15** by reacting with the corresponding benzyl bromides. NMR analysis confirmed that all bromide salts 1–15 were successfully synthesized with high yields (Figure 2, isolated yields ranging from 27% to 96%).

**Figure 3:**
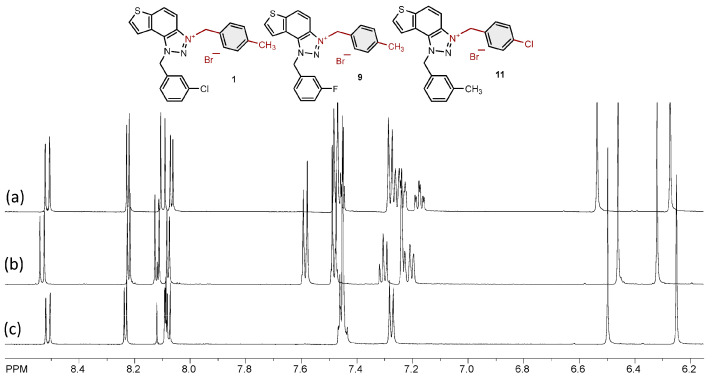
Parts of the ^1^H NMR spectra of charged triazolinium salts (a) **9**, (b) **11**, and (c) **1**.

New charged thienobenzo-1,2,3-triazolinium salts **1**–**15** were fully spectroscopically characterized (See Section 3 and Supplementary Materials). In the ^1^H NMR spectra of triazolinium salts **1**–**15**, a new signal of the second methylene group on the charged triazole nitrogen is visible between 6.1 and 6.4 ppm, undoubtedly confirming the formation of the target structures (Figure 3).

### 2.2. Cholinesterase Inhibition Activity of Triazolinium Salts ***1**–**15***

To assess the biological potency of newly synthesized thienobenzo-triazolinium salts **1**–**15** as inhibitors of acetylcholinesterase (AChE) and butyrylcholinesterase (BChE), a modified Ellman’s method [20] was used. The compounds were tested across a broad concentration range, depending on their solubility, and their inhibitory activities were compared to those of the commercially available drug donepezil. The IC_50_ values and the selectivity index for triazolinium salts **1**–**3** and **5**–**15** are listed in Table 1 (the amount of triazolinium salt **4** obtained was insufficient for cholinesterase inhibition testing). As expected, based on our previous findings [19,21], the tested triazolinium salts inhibited both enzymes, AChE and BChE. Most of the compounds showed stronger inhibition of BChE. However, enzyme selectivity varies and depends on the specific molecular structure (Table 1).

Among the tested compounds, derivative **14**, which has allyl and benzyl groups on the triazole ring, stands out as the most potent and selective BChE inhibitor. The presence of the allyl group enhances inhibitory activity, as we previously found that among the non-charged thienobenzo-triazoles, the derivative with the allyl substituent was the most potent BChE inhibitor [22,23]. Introducing a charge on the triazole ring with the benzyl group increases the inhibition of both enzymes. The IC_50_ value for BChE is in the nanomolar range at 98.0 nM, which is better than the standard donepezil (IC_50_ for BChE at 4.25 μM), while the IC_50_ for inhibiting AChE is 2.13 μM, which is much weaker than donepezil’s 23.0 nM (Table 1). Derivative **15**, with a propenyl instead of allyl, is also a strong BChE inhibitor, with an IC_50_ of 732.0 nM. In contrast, its IC_50_ for AChE is similar to that of **14**, at 3.62 μM.

The remaining tested compounds (**1**–**3** and **5**–**13**) contain two monosubstituted benzyl groups (with –Cl, –F, –OCH_3_, –NO_2_, or –CH_3_) attached to the triazole ring, while only derivative **3** has a methyl substituent on the thiophene ring. Among these derivatives, compound **9**, with a methyl group at the para-position of one benzyl ring and fluorine at the meta-position of another benzyl ring, exhibited the most potent dual inhibitory activity, with IC_50_ values of 363.0 nM for BChE and 4.80 μM for AChE. The next most potent inhibitors were derivatives **6** and **11** (Table 1), while the others exhibited a wide range of inhibitory activity toward BChE and good to moderate activity toward AChE. Overall, the synthesized charged thienobenzo-triazoles proved to be potent inhibitors of both enzymes, with activity and selectivity depending on the type, electron-withdrawing or donating effect, and position of substituents on the rings.

### 2.3. Anti-Inflammatory Activity of Triazolinium Salts ***1**–**15***

**Figure 4:**
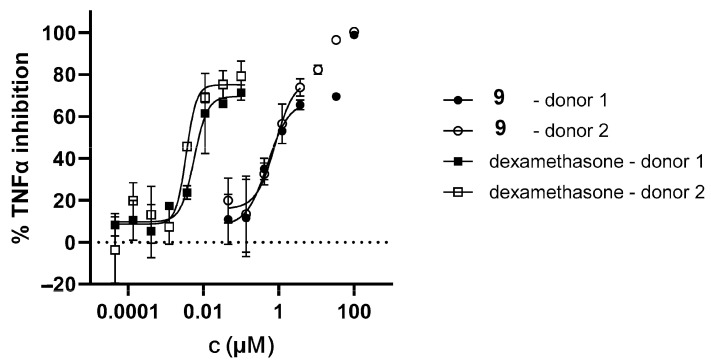
Inhibition of LPS-stimulated TNFα production in PBMCs from two donors for charged triazolinium bromide salt **9**.

To assess the potential anti-inflammatory activity of the compounds, their effects on LPS-stimulated TNF-α production in human peripheral blood mononuclear cells (PBMCs) were examined. Triazolinium salt **9** inhibited LPS-stimulated TNF-α production with an IC_50_ of 0.66 µM (Figure 4). The compound affected cell viability at the three highest concentrations tested, but at lower concentrations, it inhibited TNF-α production without reducing cell numbers. Conversely, compounds **1**, **2**, **4**, **5**, **8**, and **10**–**13** decreased cell viability, but at concentrations that did not reduce cell number, they did not inhibit TNF-α production. Compounds **3**, **6**, and **7** did not inhibit TNF-α production at any tested concentrations. Consistent with earlier results, the control compound, dexamethasone, inhibited LPS-stimulated TNF-α production with an IC_50_ of 4.6 nM (Figure 4). Notably, among derivatives **1**–**3** and **5**–**13**, all containing two monosubstituted benzyl groups attached to the triazole ring, compound **9** once again showed the most potent dual inhibitory activity, with IC_50_ values of 363.0 nM for BChE and 4.80 μM for AChE (Table 1).

### 2.4. Molecular Docking of Bioactive Triazolinium Salts ***9*** and ***11***

The inhibitory activity results presented in Table 1 show that, among the tested compounds (**1**–**3** and **5**–**13**) that contain two monosubstituted benzyl groups, salts **9** and **11** demonstrate notable activity against both cholinesterases. Although not the most potent inhibitors identified, they were selected for molecular docking due to their synthetic accessibility and suitability for further analysis. Docking was performed to elucidate the structures of the non-covalent complexes they form with cholinesterases.

**Figure 5:**
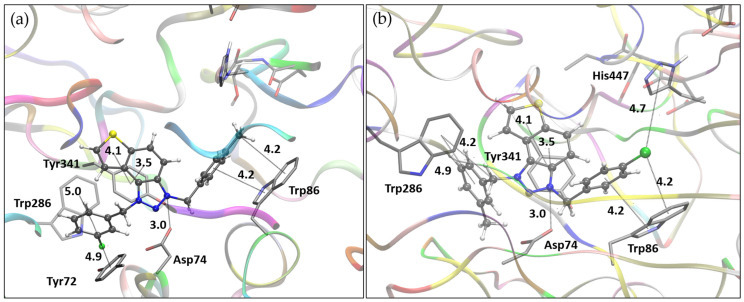
(**a**) The structure of triazolinium salt **9** docked into the active site of AChE. (**b**) Structure of triazolinium salt **11** docked into the active site of AChE. Ligands are presented using a ball-and-stick model, with distances given in angstroms.

As illustrated in Figure 5, the complexes of salts **9** and **11** with AChE reveal that both ligands adopt a similar orientation within the enzyme’s active site. The triazolinium ring is positioned toward residue Asp74 in the peripheral anionic site (PAS) due to electrostatic attraction, while the thienobenzo fragment engages in π-π stacking with Tyr341.

In the case of salt **9**, this orientation allows the meta-fluorobenzyl group attached to the triazole ring to engage in π-π interactions with Trp286 and Tyr72, both of which are located within the PAS. At the same time, the para-methylbenzyl substituent on the opposite side of the triazole ring forms π-π stacking and alkyl-π interactions with Trp86, a residue that belongs to the active site’s anionic subsite. For salt **11**, which contains a para-chlorinated benzyl group on the triazole ring, π-π stacking with Trp86 is similarly observed. Additionally, the chlorine at the para position interacts to stabilize dispersion with Trp86 and His447, with interatomic distances of approximately 4.2 Å to the centroid of the tryptophan phenyl ring and 4.7 Å to the centroid of the histidine imidazole ring.

**Figure 6:**
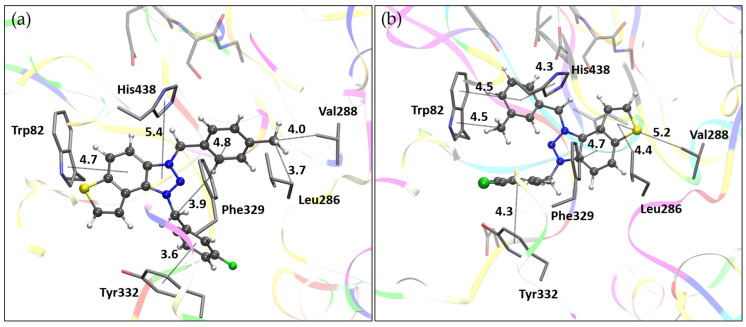
(**a**) The structure of triazolinium salt **9** docked into the active site of BChE. (**b**) Structure of triazolinium salt **11** docked into the active site of BChE. Ligands are presented using a ball-and-stick model, with distances given in angstroms.

The binding modes of salts **9** and **11** with BChE are depicted in Figure 6. Unlike their orientation in AChE, the ligands take on distinct conformations within the BChE active site. For salt **9**, the thienobenzo core engages in π-π stacking with Trp82, situated in the anionic site, while the triazole ring is oriented toward His438 in the catalytic site. The para-methylbenzyl substituent interacts with Phe329, with the methyl group directed toward the acyl pocket, comprising residues Leu286 and Val288. The second substituent, a meta-fluorobenzyl group linked by a flexible –CH_2_– spacer, participates in parallel π-π stacking with Tyr332. In the case of salt **11**, the thienobenzo fragment is positioned near the acyl pocket and also interacts with Phe329. This orientation enables the *para*-chlorobenzyl group attached to the triazolinium ring to form π-π stacking interactions with Tyr332, while the second substituent, a *meta*-methylbenzyl group, engages in π-π interactions with Trp82.

### 2.5. Genotoxicity of Triazolinium Salts ***1**–**15***

Two complementary (Q) SAR models were used to predict biological activity based on structural features [24]. In silico analyses are essential in the early stages of drug development. The commonly used software, Lhasa’s M7 package, is preferred for its use of complementary models and its practice of expert review of predictions. All compounds intended for synthesis were evaluated for in silico mutagenicity during the synthesis process (Table S3). The results showed that compounds **5**, **7**, **12**, and **13** have the potential for positive mutagenicity. However, since these are only computational predictions, all compounds were still synthesized, though they have not yet been tested for biological activity. Notably, compounds **9** and **11** have shown strong potential for biological activity (as discussed above), and given their negative mutagenicity profiles, they can be considered promising candidates for further development as lead molecules.

Both compounds exhibit limited central nervous system (CNS) permeability (Table 2), as indicated by their negative log p Values (−0.910 for compound **9** and −0.888 for compound **11**), which suggests a reduced ability to cross the blood–brain barrier (BBB) and reach CNS tissues. However, their log BB values (0.883 and 0.81, respectively) are relatively high, indicating some degree of BBB penetration. This apparent contradiction may reflect differences in the modeling methods or how the compounds interact with the BBB. Despite being P-glycoprotein substrates, which can limit CNS exposure through efflux, the moderate log BB values imply that both compounds may still penetrate the CNS to some extent. Overall, both compounds demonstrate limited potential for CNS penetration, with compound **9** showing a slightly higher theoretical capacity to cross the blood–brain barrier.

## 3. Materials and Methods

### 3.1. General Remarks

NMR spectra were obtained using either a Bruker AV300 or AV600 spectrometer (Bruker BioSpin GmbH, Rheinstetten, Germany) with a 5 mm probe. Standard ^1^H and proton-decoupled ^13^C{1 H} NMR spectra were recorded at a frequency of 600.130 MHz for ^1^H and 75.432 and 150.903 MHz for ^13^C. Chemical shifts (*δ*/ppm) for both ^1^H and ^13^C NMR spectra were referenced to the tetramethylsilane (TMS) signal. All spectra were measured in deuterated chloroform (CD_3_OD) at 25 °C. Photochemical reactions were carried out in a 50.0 mL solution in quartz cuvettes that allowed light transmission. For this purpose, a Luzchem photochemical reactor equipped with UV lamps (16) with a wavelength of 300 nm was used. All solvents used in this work were purified by distillation and were commercially available. The phosphonium salts were synthesized in our laboratory, and 1-(4-nitrophenyl)-1*H*-1,2,3-triazole-4-carbaldehyde used was previously synthesized in our laboratory [25]. Reaction progress was monitored via thin-layer chromatography (TLC) using silica gel-coated plates (0.2 mm, 60/Kieselguhr F254), placed in 10 mL of the appropriate solvent system. After each synthesis, the reaction mixture was cooled to 0 °C, and then a certain amount of diethyl ether was added to precipitate the product. The resulting suspension was centrifuged (Centrifuge Eba 20, Hettich, Tuttlingen, Germany) at 2 × 3000 rpm for 10 min and then at 5 × 5000 rpm for 10 min. It was then decanted and finally evaporated. High-resolution mass spectrometry (HRMS) analyses were performed on a MALDI TOF/TOF analyzer, using an Nd:YAG laser at 355 nm with a fitting rate of 200 Hz.

### 3.2. Synthesis of Bromide Salts ***1**–**15***

Triazole photoproducts (Scheme 1) previously developed by our group [19,21,22,23] served as the starting materials for the synthesis of bromide salts **1**–**15**. In a small vial, triazole analogues were dissolved in 0.6 mL of dry DCM and then purged briefly with argon. Furthermore, 20 eq of the corresponding benzyl bromide was added, and the reaction vial was then stirred in an oil bath at 60 °C for 24 h. Afterward, the reaction mixture was cooled to 0 °C, and then approximately 5 mL of diethyl ether was added, forming a white suspension. At the end, the suspension was centrifuged at 2 × 3000 rpm for 10 min and then at 5 × 5000 rpm for 10 min, before being decanted and evaporated using a rotary evaporator. According to NMR analyses, all bromide salts **1**–**15** were successfully synthesized in mostly high yields.

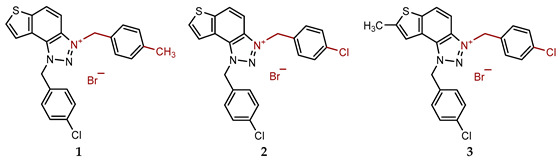

1-(4-chlorobenzyl)-3-(4-methylbenzyl)-1*H*-thieno[3′,2′:3,4]benzo[1,2-*d*][1,2,3]triazol-3-ium bromide (**1**): 9 mg (isolated 69%), white powder; m.p. 117–118 °C; ^1^H NMR (CD_3_OD, 600 MHz) *δ*/ppm: 8.51 (d, *J* = 9.2 Hz, 1H), 8.23 (d, *J* = 5.5 Hz, 1H), 8.10–8.06 (m, 2H), 7.47–7.43 (m, 6H), 7.28 (d, *J* = 8.1 Hz, 2H), 6.50 (s, 2H), 6.25 (s, 2H), 2.36 (s, 3H); ^13^C NMR (CD_3_OD, 150 MHz) *δ*/ppm: 143.0, 139.7, 135.1, 134.0, 133.1, 130.9, 130.7, 129.7, 129.4, 129.2, 129.0, 128.5, 126.9, 123.1, 120.4, 107.9, 55.3, 55.2, 19.8; HRMS (ESI) (*m*/*z*) for C_23_H_19_ClN_3_S^+^ Br^−^: [M + H]^+^_calcd_ = 404.0988, and [M + H]^+^_measured_ = 404.0997.
1,3-bis(4-chlorobenzyl)-1*H*-thieno[3′,2′:3,4]benzo[1,2-*d*][1,2,3]triazol-3-ium bromide (**2**): 13 mg (isolated 96%), white powder; m.p. 108–110 °C; ^1^H NMR (CD_3_OD, 600 MHz) *δ*/ppm: 8.54 (d, *J* = 9.2 Hz, 1H), 8.25 (d, *J* = 5.7 Hz, 1H), 8.13–8.08 (m, 2H), 7.57 (d, *J* = 8.6 Hz, 2H), 7.50–7.43 (m, 6H), 6.50 (s, 2H), 6.30 (s, 2H); ^13^C NMR (CD_3_OD, 150 MHz) *δ*/ppm: 143.1, 135.1, 135.40, 134.2, 133.2, 131.0, 130.7, 130.6, 130.3, 129.5, 129.4, 129.2, 127.2, 123.2, 120.4, 107.7, 55.4, 54.4.; HRMS (ESI) (*m*/*z*) for C_22_H_16_Cl_2_N_3_S^+^Br^−^: [M + H]^+^_calcd_ = 424.0442, and [M + H]^+^_measured_ = 424.0448.
1,3-bis(4-chlorobenzyl)-7-methyl-1*H*-thieno[3′,2′:3,4]benzo[1,2-*d*][1,2,3]triazol-3-ium bromide (**3**): 10 mg (isolated 95%), white powder; m.p. 105–107 °C; ^1^H NMR (CD_3_OD, 600 MHz) *δ*/ppm: 8.39 (d, *J* = 9.2 Hz, 1H), 8.00 (d, *J* = 9.2 Hz, 1H), 7.83 (s, 1H), 7.54 (d, *J* = 8.7 Hz, 2H), 7.49–7.43 (m, 6H), 6.45 (s, 2H), 6.26 (s, 2H), 2.77 (s, 3H); ^13^C NMR (CD_3_OD, 75 MHz) *δ*/ppm: 150.2, 131.7, 131.0, 130.6, 129.8, 129.4, 119.9, 108.0, 55.7, 56.7, 16.1 (several signals are missing due to the small quantity of the sample); HRMS (ESI) (*m*/*z*) for C_23_H_18_C_l2_N_3_S^+^Br^−^: [M + H]^+^_calcd_ = 438.0598, and [M + H]^+^_measured_ = 438.0610.

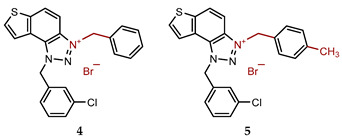

3-benzyl-1-(3-chlorobenzyl)-1*H*-thieno[3′,2′:3,4]benzo[1,2-*d*][1,2,3]triazol-3-ium bromide (**4**): 5 mg (isolated 27%), white powder; m.p. 119–120 °C; ^1^H NMR (CD_3_OD, 600 MHz) *δ*/ppm: 8.53 (d, *J* = 9.1 Hz, 1H), 8.25 (d, *J* = 5.7 Hz, 1H), 8.13–8.09 (m, 3H), 7.57 (d, *J* = 7.8 Hz, 2H), 7.52 (s, 1H), 7.49–7.40 (m, 5H), 6.52 (s, 2H), 6.31 (s, 2H); ^13^C NMR (CD_3_OD, 150 MHz) *δ*/ppm: 143.1, 135.0, 134.1, 133.2, 132.1, 131.0, 130.6, 129.4, 129.3, 129.1, 128.5, 127.8, 127.0, 126.1, 123.1, 120.4, 107.8, 55.3; MS (ESI) (*m*/*z*, %) for C_22_H_17_ClN_3_S^+^Br^−^: [M + H]^+^ 391 (100), 250 (85).
1-(3-chlorobenzyl)-3-(4-methylbenzyl)-1*H*-thieno[3′,2′:3,4]benzo[1,2-*d*][1,2,3]triazol-3-ium bromide (**5**): 11 mg (isolated 57%), white powder; m.p. 104–105 °C; ^1^H NMR (CD_3_OD, 600 MHz) *δ*/ppm: 8.51 (d, *J* = 9.2 Hz, 1H), 8.24 (d, *J* = 5.4 Hz, 1H), 8.12–8.08 (m, 2H), 7.53–7.36 (m, 6H), 7.28 (d, *J* = 8.0 Hz, 2H), 6.51 (s, 2H), 6.26 (s, 2H), 2.36 (s, 3H); ^13^C NMR (CD_3_OD, 150 MHz) *δ*/ppm: 144.5, 141.1, 136.4, 135.5, 135.4, 134.5, 132.4, 132.0, 131.1, 130.7, 130.0, 129.2, 128.4, 127.5, 124.5, 121.8, 109.3, 56.7, 56.6, 21.2; HRMS (ESI) (*m*/*z*) for C_23_H_19_ClN_3_S^+^Br^−^: [M + H]^+^_calcd_ = 404.0988, and [M + H]^+^_measured_ = 404.0998.

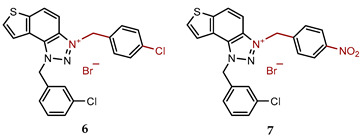

1-(3-chlorobenzyl)-3-(4-chlorobenzyl)-1*H*-thieno[3′,2′:3,4]benzo[1,2-*d*][1,2,3]triazol-3-ium bromide (**6**): 8 mg (isolated 40%), white powder; m.p. 111–113 °C; ^1^H NMR (CD_3_OD, 600 MHz) *δ*/ppm: 8.55 (d, *J* = 9.2 Hz, 1H), 8.26 (d, *J* = 5.5 Hz, 1H), 8.14–8.10 (m, 2H), 7.58 (d, *J* = 8.8 Hz, 2H), 7.52 (t, *J* = 1.5, 3.4 Hz, 1H), 7.50–7.41 (m, 4H), 7.38 (dt, *J* = 1.5, 3.3 Hz, 1H), 6.49 (s, 2H), 6.28 (s, 2H); ^13^C NMR (CD_3_OD, 150 MHz) *δ*/ppm: 143.2, 135.4, 134.9, 134.1, 133.8, 133.2, 130.6, 130.3, 129.2, 129.3, 127.9, 127.2, 126.2, 123.1, 120.3 55.3, 54.3; HRMS (ESI) (*m*/*z*) for C_22_H_16_Cl_2_N_3_S^+^Br^−^: [M + H]^+^_calcd_ = 424.0442, and [M + H]^+^_measured_ = 424.0447.
1-(3-chlorobenzyl)-3-(4-nitrobenzyl)-1*H*-thieno[3′,2′:3,4]benzo[1,2-*d*][1,2,3]triazol-3-ium bromide (**7**): 9 mg (isolated 42%), white powder; m.p. 125–126 °C; ^1^H NMR (CD_3_OD, 600 MHz) *δ*/ppm: 8.57 (d, *J* = 9.2 Hz, 1H), 8.32 (d, *J* = 8.7 Hz, 2H), 8.27 (d, *J* = 5.7 Hz, 1H), 8.15–8.11 (m, 3H), 7.80–7.77 (m, 2H), 7.47–7.38 (m, 3H) 6.53 (s, 2H), 6.47 (s, 2H); ^13^C NMR (CD_3_OD, 75 MHz) *δ*/ppm: 140.3, 136.4, 135.9, 135.3, 134.8, 132.1, 131.1, 130.6, 130.7, 129.4, 128.8, 127.7, 125.4, 124.6, 123.9, 121.8, 110.0, 56.9, 55.4; HRMS (ESI) (*m*/*z*) for C_22_H_16_ClN_4_O_2_S^+^Br^−^: [M + H]^+^_calcd_ = 435.0682, and [M + H]^+^_measured_ = 435.0690.

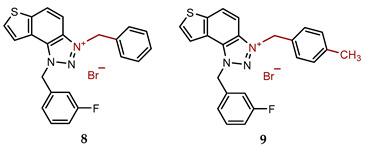

3-benzyl-1-(3-fluorobenzyl)-1*H*-thieno[3′,2′:3,4]benzo[1,2-*d*][1,2,3]triazol-3-ium bromide (**8**): 7 mg (isolated 48%), white powder; m.p. 103–104 °C; ^1^H NMR (CD_3_OD, 600 MHz) *δ*/ppm: 8.53 (d, *J* = 9.1 Hz, 1H), 8.23 (d, *J* = 5.5 Hz, 1H), 8.13–8.05 (m, 3H), 7.58 (dd, *J* = 1.8, 8.0 Hz, 2H), 7.49–7.42 (m, 4H), 7.27–7.21 (m, 2H), 6.53 (s, 2H), 6.31 (s, 2H); ^13^C NMR (CD_3_OD, 75 MHz) *δ*/ppm: 145.3 (d, *J_CF_* = 236.7 Hz), 143.7, 139.1, 138.0, 137.1, 134.9, 134.6, 133.6, 133.4, 133.1, 132.9, 132.5, 130.9, 127.1, 124.3, 111.8, 59.3, 59.2 (signals for four quaternary C are missing); HRMS (ESI) (*m*/*z*) for C_22_H_17_FN_3_S^+^Br^−^: [M + H]^+^_calcd_ = 374.1127, and [M + H]^+^_measured_ = 374.1131.
1-(3-fluorobenzyl)-3-(4-methylbenzyl)-1*H*-thieno[3′,2′:3,4]benzo[1,2-*d*][1,2,3]triazol-3-ium bromide (**9**): 14 mg (isolated 93%), white powder; m.p. 116–117 °C; ^1^H NMR (CD_3_OD, 600 MHz) *δ*/ppm: 8.51 (dd, *J* = 0.7, 9.2 Hz, 1H), 8.22 (d, *J* = 5.2 Hz, 1H), 8.12–8.05 (m, 2H), 7.50–7.43 (m, 3H), 7.30–7.22 (m, 4H), 7.19–7.15 (m, 1H), 6.53 (s, 2H), 6.27 (s, 2H), 2.36 (s, 3H); ^13^C NMR (CD_3_OD, 75 MHz) *δ*/ppm: 167.1 (d, *J_CF_* = 246.2 Hz), 147.0, 143.7, 138.4 (d, *J_CF_* = 8.0 Hz), 138.0, 137.0, 135.0 (d, *J_CF_* = 8.7 Hz), 133.6, 132.9, 132.5, 130.9, 127.4, 127.3, 127.0, 124.3, 119.9 (d, *J_CF_* = 21.7 Hz), 118.5 (d, *J_CF_* = 23.3 Hz), 59.3, 59.2, 23.7; HRMS (ESI) (*m*/*z*) for C_23_H_19_FN_3_S^+^Br^−^: [M + H]^+^_calcd_ = 388.1284, and [M + H]^+^_measured_ = 388.1292.

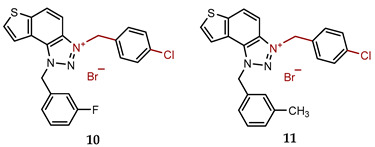

3-(4-chlorobenzyl)-1-(3-fluorobenzyl)-1*H*-thieno[3′,2′:3,4]benzo[1,2-*d*][1,2,3]triazol-3-ium bromide (**10**): 7 mg (isolated 45%), white powder; m.p. 110–112 °C; ^1^H NMR (CD_3_OD, 600 MHz) *δ*/ppm: 8.55 (dd, *J* = 0.7, 9.2 Hz, 1H), 8.24 (d, *J* = 5.5 Hz, 1H), 8.14–8.10 (m, 1H), 8.08–8.06 (m, 1H), 7.58 (d, *J* = 8.6 Hz, 2H), 7.50–7.44 (m, 3H), 7.29–7.15 (m, 3H), 6.53 (s, 2H), 6.31 (s, 2H); ^13^C NMR (CD_3_OD, 150 MHz) *δ*/ppm: 143.2, 136.3 (d, *J_CF_* = 216.2 Hz), 135.4, 134.2, 133.6, 133.2, 132.7, 131.1 (d, *J_CF_* = 8.1 Hz), 130.7, 130.3, 129.2, 127.2, 123.5, 123.1, 120.4, 115.9 (d, *J_CF_* = 21.9 Hz), 114.6 (d, *J_CF_* = 23.2 Hz), 107.7, 55.4, 54.5 (signals for 2 quaternary C are missing); HRMS (ESI) (*m*/*z*) for C_22_H_16_ClFN_3_S^+^Br^−^: [M + H]^+^_calcd_ = 408.0738, and [M + H]^+^_measured_ = 408.0745.
3-(4-chlorobenzyl)-1-(3-methylbenzyl)-1*H*-thieno[3′,2′:3,4]benzo[1,2-*d*][1,2,3]triazol-3-ium bromide (**11**): 8 mg (isolated 94%), white powder; m.p. 122–123 °C; ^1^H NMR (CD_3_OD, 600 MHz) *δ*/ppm: 8.53 (d, *J* = 9.2 Hz, 1H), 8.22 (d, *J* =5.5 Hz, 1H), 8.14–8.06 (m, 2H), 7.58 (d, *J* = 8.6 Hz, 2H), 7.48 (d, *J* = 8.5 Hz, 2H), 7.32–7.17 (m, 4H), 6.46 (s, 2H), 6.32 (s, 2H), 2.32 (s, 3H); ^13^C NMR (CD_3_OD, 75 MHz) *δ*/ppm: 144.5, 140.7, 136.8, 135.6, 134.5, 133.2, 132.4, 131.7, 131.2, 130.6, 130.4, 129.4, 128.5, 125.9, 124.6, 122.0, 109.1, 55.8, 57.5, 21.3; HRMS (ESI) (*m*/*z*) for C_23_H_19_ClN_3_S^+^Br^−^: [M + H]^+^_calcd_ = 404.0988, and [M + H]^+^_measured_ = 404.0996.

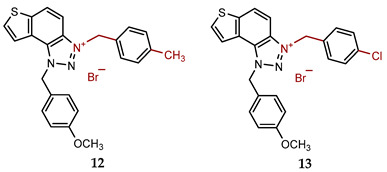

1-(4-methoxybenzyl)-3-(4-methylbenzyl)-1*H*-thieno[3′,2′:3,4]benzo[1,2-*d*][1,2,3]triazol-3-ium bromide (**12**): 9 mg (isolated 95%), white powder; m.p. 120–122 °C; ^1^H NMR (CD_3_OD, 600 MHz) *δ*/ppm: 8.49 (d, *J* = 9.5 Hz, 1H), 8.22 (d, *J* = 5.5 Hz, 1H), 8.15–8.11 (m, 1H), 8.06 (d, *J* = 9.2 Hz, 1H), 7.44 (d, *J* = 8.2 Hz, 2H), 7.39 (d, *J* = 8.9 Hz, 2H), 7.27 (d, *J* = 8.2 Hz, 2H), 6.97 (d, *J* = 8.9 Hz, 2H), 6.42 (s, 2H), 6.24 (s, 2H), 3.79 (s, 3H), 2.35 (s, 3H); ^13^C NMR (CD_3_OD, 150 MHz) *δ*/ppm: 142.9, 139.7, 134.3, 133.9, 132.9, 130.8, 129.7, 129.2, 129.1, 128.4, 126.8, 123.6, 123.2, 120.6, 114.3, 107.8, 65.4, 54.3, 53.4, 19.8; HRMS (ESI) (*m*/*z*) for C_24_H_22_N_3_OS^+^Br^−^: [M + H]^+^_calcd_ = 400.1484, and [M + H]^+^_measured_ = 400.1487.
3-(4-chlorobenzyl)-1-(4-methoxybenzyl)-1*H*-thieno[3′,2′:3,4]benzo[1,2-*d*][1,2,3]triazol-3-ium bromide (**13**): 7 mg (isolated 74%), white powder; m.p. 124–125 °C; ^1^H NMR (CD_3_OD, 600 MHz) *δ*/ppm: 8.52 (dd, *J* = 0.7, 9.2 Hz, 1H), 8.24 (d, *J* = 5.5 Hz, 1H), 8.15 (d, *J* = 5.5 Hz, 1H), 8.09 (d, *J* = 9.2 Hz, 1H), 7.36 (d, *J* = 8.6 Hz, 2H), 7.47 (d, *J* = 8.6 Hz, 2H), 7.40 (d, *J* = 8.8 Hz, 2H), 7.00 (d, *J* = 8.7 Hz, 2H), 6.42 (s, 2H), 6.29 (s, 2H), 3.80 (s, 3H); ^13^C NMR (CD_3_OD, 75 MHz) *δ*/ppm: 134.5, 132.3, 131.6, 130.7, 130.6, 128.5, 125.5, 124.9, 122.1, 115.8, 109.1, 57.3, 55.8, 55.7 (signals for 5 quaternary C are missing); HRMS (ESI) (*m*/*z*) for C_23_H_19_ClN_3_OS^+^Br^−^: [M + H]^+^_calcd_ = 420.0937, and [M + H]^+^_measured_ = 420.0943.

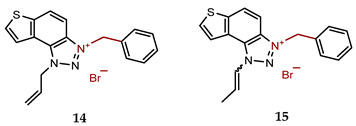

1-allyl-3-benzyl-1*H*-thieno[3′,2′:3,4]benzo[1,2-*d*][1,2,3]triazol-3-ium bromide (**14**): 8 mg (isolated 64%), white powder; m.p. 101–102 °C; ^1^H NMR (CD_3_OD, 300 MHz) *δ*/ppm: 8.52 (d, *J* = 9.2 Hz, 1H), 8.29 (d, *J* = 5.5 Hz, 1H), 8.18 (d, *J* = 5.6 Hz, 1H), 8.14–8.05 (m, 2H), 7.61–7.53 (m, 2H), 7.49–7.41 (m, 2H), 6.40–6.32 (m, 1H), 6.30 (s, 2H), 5.92 (d, *J* = 5.5 Hz, 2H), 5.52 (d, *J* = 10.4 Hz, 1H), 5.41 (d, *J* = 17.1 Hz, 1H); ^13^C NMR (CD_3_OD, 75 MHz) *δ*/ppm: 142.9, 133.9, 133.02, 132.1, 129.3, 129.1, 128.8, 128.5, 126.9, 123.2, 120.7, 120.2, 114.5, 107.7, 55.2, 54.9; MS (EI) (*m*/*z*, %) for C_18_H_16_N_3_S^+^Br^−^: 306 (100).
3-benzyl-1-(prop-1-en-1-yl)-1*H*-thieno[3′,2′:3,4]benzo[1,2-*d*][1,2,3]triazol-3-ium bromide (**15**): 11 mg (isolated 51%), yellow oil; mixture of *cis*- and *trans*-isomer; MS (EI) (*m*/*z*, %) for C_18_H_16_N_3_S^+^Br^−^: 306 (100).

### 3.3. In Vitro Cholinesterase Inhibition Activity Measurements

The inhibition of AChE and BChE was determined using a modified Ellman’s spectrophotometric assay [20]. Enzymes AChE (EC 3.1.1.7, *Electrophorus electricus*, Type V-S) and BChE (EC 3.1.1.8, equine serum) were purchased from Sigma-Aldrich (St. Louis, MO, USA). All the remaining chemicals, Trisma base (2-amino-2-(hydroxymethyl)-1,3-propanediol), acetylthiocholine iodide (ATChI), *S*-butyrylthiocholine iodide (BTChI), and standard Donepezil, were also purchased from Sigma-Aldrich, while Ellman’s reagent 5,5-dithiobis-(2-nitrobenzoic acid) (DTNB) was sourced from Zwijndrecht (Antwerpen, Belgium). The various concentrations of the tested compounds dissolved in ethanol were added (10 µL) to 180 µL of Tris buffer (50 mM, pH 8.0), 10 µL of enzyme solution (final concentration 0.03 U/mL), and 10 µL of DTNB (final concentration 0.3 mM). The enzymatic reaction was initiated by the addition of 10 µL of substrate ATChI or BTChI, resulting in a final substrate concentration of 0.5 mM. Non-enzymatic hydrolysis was measured as a blank for control measurement without inhibitors. The non-enzymatic hydrolysis reaction with added inhibitor was used as a blank for the samples. The enzyme was replaced with an equivalent amount of buffer. Absorbance was measured at 405 nm over 5 min at room temperature using a 96-well microplate reader (BioTek 800TSUV Absorbance Reader, Agilent, Santa Clara, CA, USA). All measurements were conducted in triplicate. The percentage of inhibition was calculated using the following equation: Inhibition (%) = [(*A*_C_ − *A*_T_)/*A*_C_] × 100, where AC represents the enzyme activity in the absence of the test sample, and AT represents the enzyme activity in the presence of the test compound. Inhibition data were used to calculate the IC_50_ value by nonlinear fitting of the inhibitor concentration versus response. The inhibitory activity of ethanol was also measured, and its contribution to inhibition was subtracted. The IC_50_ value is represented as a mean value of three measurements ± standard deviation. The selectivity index is calculated as IC_50_(AChE)/IC_50_(BChE).

### 3.4. Anti-Inflammatory Activity

The effect of triazolinium salts **1**–**15** on lipopolysaccharide (LPS)-stimulated tumor necrosis factor alpha (TNF-α) production was analyzed as described before [26]. Peripheral blood mononuclear cells (PBMCs) were isolated from buffy coats obtained from healthy adult volunteers and resuspended in RPMI1640 medium (Capricorn Scientific, Ebsdorfergrund, Germany) supplemented with 10% heat-inactivated FBS (Biowest, Riverside, MO, USA), 1% GlutaMAX (Gibco, Waltham, MA, USA), and 1% Antibiotic-Antimycotic (Gibco). In a 96-well plate, 2 × 10^5^ PBMCs were seeded per well. Triazolinium salts **1**–**15** were dissolved in dimethyl sulfoxide solvent (DMSO, Sigma), and three-fold serial dilutions in DMSO were assembled and added to the cells, starting at a concentration of 100 µM. After a 1-h pre-incubation with triazolinium salts **1**–**15**, cells were stimulated with 1 ng/mL LPS from *E. coli* 0111:B4 (Sigma). Cells were incubated for 24 h at 37 °C in 5% CO_2_, followed by the collection of supernatants for TNF-α measurement and cell viability assessment. To study cell viability, CellTiter-Glo reagent (Promega, Madison, WI, USA) was applied according to the manufacturer’s instructions, and signals obtained from compound-treated wells were compared with those in LPS-stimulated, vehicle-treated samples.

TNF-α concentration in supernatants was measured by ELISA utilizing antibodies and recombinant human TNF-α protein (standard) from R&D Systems (Minneapolis, MN, USA). Lumitrac 600 384-well plates (Greiner Bio-One, Kremsmünster, Austria) were coated with 1 µg/mL of TNF-α capture antibody diluted in phosphate-buffered saline (PBS; Gibco) overnight at 4 °C. The next day, plates were blocked with 5% sucrose (Kemika, Ovada, Italy) in assay diluent (1% bovine serum albumin [BSA; Sigma] in PBS) for 4 h at room temperature (RT). After blocking, samples and standards from triazolinium salts **1**–**15** were joined to the plates and incubated overnight at 4 °C. Subsequently, 250 ng/mL of TNF-α detection antibody was added to the wells, followed by a 2-h incubation at RT. Finally, after incubation with streptavidin-HRP (Invitrogen, Waltham, MA, USA), chemiluminescence ELISA Substrate (Roche, Basel, Switzerland) was added, and luminescence was measured using an EnVision 2105 multilabel reader (Revvity, Waltham, MA, USA). Measured luminescence was used to determine the concentrations of TNF-α in the supernatants by interpolation from the standard curve. Percentages of inhibition were calculated from the cytokine concentrations obtained, and IC_50_ values were determined using GraphPad Prism v9 software with a nonlinear regression curve fit (four parameters with variable slope).

### 3.5. Computational Details

Conformational analysis and geometry optimization of two compounds chosen for the docking study were carried out using the Gaussian16 program package [27], at the B3LYP/6-31G(d) level of theory. The most stable conformers were used as ligands in the molecular docking process, conducted with the AutoDock4 suite [28]. Crystal structures 4EY7.pdb and 1P0I.pdb, representing AChE and BChE, respectively, were downloaded from the Protein Data Bank [29,30] and prepared for docking (non-amino acid residues removed, polar hydrogens added, and Kollman charges assigned). The Lamarckian Genetic Algorithm was used for docking, with 25 runs per ligand, while the enzyme residues remained rigid.

### 3.6. ADME-Tox Predictions

pkCSM is a free-to-use machine learning programme that anticipates small-molecule pharmacokinetic values using graph-based signatures. It covers 28 models with key ADMET effects, such as permeability, solubility, absorption, distribution in the body, interactions with metabolic enzymes, excretion, and various toxicity measures. Swiss ADME permits computing physicochemical descriptors for free and predicts ADME parameters, pharmacokinetic properties, drug-like nature, and medicinal chemistry friendliness for one or multiple small molecules to support drug discovery. AdmetSAR 3.0 is a free-access chemical risk assessment tool [31]. ADMETLAb 3.0 provides easy access to comprehensive, accurate, and efficient predictions of ADMET profiles for compounds. These predictions are based on a high-quality database of 0.37 million entries spanning 77 endpoints and the Directed Message Passing Neural Network (DMPNN) framework. For this paper, all triazolinium salts **1**–**15** were evaluated using multiple tools, and predictions of key features for the lead molecules were presented.

## 4. Conclusions

The findings of this study demonstrate that the newly synthesized charged thienobenzo-1,2,3-triazole derivatives exhibit strong inhibitory activity against both acetylcholinesterase (AChE) and butyrylcholinesterase (BChE), with a clear tendency toward higher potency and selectivity for BChE. This makes them promising candidates for treating disorders that require peripheral cholinesterase inhibition. Introducing a permanent positive charge on the triazole ring was a key structural modification that significantly enhanced enzyme binding affinity, particularly for BChE. Among all of the tested compounds, derivative 14 stood out with sub-micromolar inhibition of both enzymes and excellent selectivity. Meanwhile, compound **9** demonstrated not only potent dual inhibition but also notable anti-inflammatory activity in a TNF-α suppression assay. These dual actions suggest that certain charged triazole derivatives could serve as multifunctional drug candidates, combining cholinesterase inhibition with immunomodulatory effects. This is especially relevant for neurodegenerative diseases involving both cholinergic deficits and chronic inflammation, such as Alzheimer’s disease. Additionally, molecular docking studies provided structural insights into ligand-enzyme interactions, supporting the biological data and confirming stable and specific binding of ligands to key residues in the active sites of AChE and BChE. Besides biological testing, in silico ADME-Tox profiling of selected lead compounds indicated favorable pharmacokinetic and safety profiles, including good solubility, moderate permeability, non-mutagenicity, and predictable metabolism. These properties, along with their synthetic accessibility and chemical stability, enhance the potential of these molecules for further development in preclinical studies. In summary, this work lays a solid foundation for designing new charged heterocyclic compounds with dual therapeutic functions. The promising in vitro and in silico results of derivatives **9** and **14** highlight the importance of structural charge modulation in optimizing cholinesterase inhibitors that are selective for the periphery and offer additional therapeutic benefits.

## Figures and Tables

**Table 1:** Calculated IC_50_ values for the inhibition of AChE and BChE by the charged triazolinium salts **1**–**3** and **5**–**15**.

Compound	AChE IC_50_/μM	BChE IC_50_/μM	Selectivity Index
**1**	12.74 ± 1.85	2.55 ± 0.58	5.0
**2**	10.71 ± 1.46	4.99 ± 1.10	2.1
**3**	8.89 ± 3.26	7.60 ± 0.82	1.2
**5**	55.62 ± 2.36	8.13 ± 0.83	6.8
**6**	12.90 ± 0.87	0.96 ± 0.14	13.4
**7**	11.42 ± 0.39	10.03 ± 1.64	1.1
**8**	22.23 ± 2.83	4.52 ± 0.17	4.9
**9**	4.80 ± 0.71	0.363 ± 0.038	13.2
**10**	9.96 ± 0.42	2.45 ± 0.43	4.1
**11**	6.23 ± 0.58	1.11 ± 0.77	5.6
**12**	11.81 ± 2.15	3.98 ± 1.25	3.0
**13**	7.40 ± 0.94	4.05 ± 1.05	1.8
**14**	2.13 ± 0.13	0.098 ± 0.018	21.7
**15**	3.62 ± 0.42	0.732 ± 0.137	4.9
**Donepezil**	0.023 ± 0.004	4.25 ± 0.09	0.005

**Table 2:** In silico analysis of additional ADME (T) indicators—absorption, distribution, metabolism, excretion, and toxicity—in the human body for new charged thienobenzo-1,2,3-triazolinium salts **9** and **11**.

Property	Model Name	9	11	Unit
**Absorption**	Water solubility	−5.103	−5.31	log mol/L
	Caco2	0.814	0.812	log Papp in 10^−6^ cm/s
	Intestinal absorption	100	100	% Absorbed
	Skin permeability	−2.732	−2.732	log Kp
	P–glycoprotein substrate	Yes	Yes	
	P–glycoprotein I inhibitor	No	No	
	P–glycoprotein II inhibitor	Yes	Yes	
**Distribution**	VDss (human)	−0.333	−0.186	log L/kg
	Fraction unbound	0.299	0.289	Fu
	BBB permeability	0.883	0.81	log BB
	CNS permeability	−0.91	−0.888	log PS
**Metabolism**	CYP2D6 substrate	No	No	
	CYP3A4 substrate	Yes	Yes	
	CYP1A2 inhibitor	Yes	Yes	
	CYP2C19 inhibitor	Yes	No	
	CYP2C9 inhibitor	No	No	
	CYP2D6 inhibitor	Yes	Yes	
	CYP3A4 inhibitor	No	No	
**Excretion**	Total clearance	0.215	1.231	log ml/min/kg
	Renal OCT2 substrate	No	No	Yes/No

## Data Availability

Dataset available on request from the authors.

## Supplementary Materials

The following supporting information can be downloaded at https://www.mdpi.com/article/10.3390/ph18071032/s1: ^1^H and ^13^C NMR spectra of new charged triazolinium salts **1**–**15** (Figures S1–S30); Mass spectra and HRMS analyses of new charged triazolinium salts **1**–**15** (Figures S31–S43); Cartesian coordinates of ligands **9** and **11** docked into the active site of AChE; Cartesian coordinates of ligands **9** and **11** docked into the active site of BChE; Free energies of binding, the number of conformational clusters, and distribution of conformations obtained by molecular docking (Table S1. Free energies of binding, *ΔG*_bind_, obtained by molecular docking of ligands **9**, **11**, and reference ligand donepezil into the active site of AChE (4EY7.pdb), along with the number of conformational clusters and distribution of conformations and Table S2. Free energies of binding, *ΔG*_bind_, obtained by molecular docking of ligands **9**, **11**, and reference ligand donepezil into the active site of BChE (1P0I.pdb), along with the number of conformational clusters and distribution of conformations); The mutagenic potential of new charged thienobenzo-1,2,3-triazolinium salts **1**–**15** through Lhasa M7 evaluation (Table S3).
