# Supplementary material for: Charged Thienobenzo-1,2,3-Triazoles as Especially Potent Non-Selective Cholinesterase Inhibitors: Design, Anti-Inflammatory Activity, and Computational Study
Source: Pharmaceuticals (Basel). 2025 Jul 11;18(7):1032. doi: 10.3390/ph18071032 (PMC12299554; doi:10.3390/ph18071032)
Supplement: Supplementary file 1 [file pharmaceuticals-18-01032-s001.zip › pharmaceuticals-3704784-supplementary.pdf]

Electronic Supporting Information

Article

Charged Thienobenzo-1,2,3-Triazoles as Especially Potent Non-Selective Cholinesterase Inhibitors: Design, Anti-Inflammatory Activity, and Computational Study

Antonija Jelčić [1,†], Anamarija Raspudić [2,†], Danijela Barić [3], Ana Ratković [1], Ivana Šagud [4], Paula Pongrac [5], Dora Štefok [5], Martina Bosnar [5], Sunčica Roca [6], Zlata Lasić [7], Ilijana Odak [2,*] and Irena Škorić [1,*]

[1] Department of Organic Chemistry, Faculty of Chemical Engineering and Technology, University of Zagreb, Trg Marka Marulića 19, HR-10 000 Zagreb, Croatia; ajelcic@fkit.unizg.hr (A.J.); ana.ratkovic30@gmail.com (A.R.)
[2] Department of Chemistry, Faculty of Science and Education, University of Mostar, Matice Hrvatske bb, 88 000 Mostar, Bosnia and Herzegovina; anamarija.raspudic@fpmoz.sum.ba
[3] Group for Computational Life Sciences, Division of Physical Chemistry, Ruđer Bošković Institute, Bijenička Cesta 54, HR-10 000 Zagreb, Croatia; dbaric@irb.hr
[4] Croatian Agency for Medicinal Products and Medical Devices, Ksaverska Cesta 4, HR-10 000 Zagreb, Croatia; ivana.sagud@halmed.hr
[5] Pharmacology In Vitro, Selvita Ltd., Prilaz Baruna Filipovića 29, HR-10 000 Zagreb, Croatia; paula.pongrac@selvita.com (P.P.); dora.stefok@selvita.com (D.Š.); martina.bosnar@selvita.com (M.B.)
[6] NMR Center, Ruđer Bošković Institute, Bijenička Cesta 54, HR-10 000 Zagreb, Croatia; sroca@irb.hr
[7] TEVA Global R&D, E&L R&D, Pliva Hrvatska d.o.o., Prilaz Baruna Filipovića 25, HR-10 000 Zagreb, Croatia; zlata.lasic01@pliva.com
* Correspondence: ilijana.odak@fpmoz.sum.ba (I.O.); iskoric@fkit.unizg.hr (I.Š.)
† These authors contributed equally to this work.

Table of contents:

1. ^{1}H and ^{13}C NMR spectra of new charged triazole salts **1–15**
2. Mass spectra and HRMS analyses of new charged triazole salts **1–15**
3. Cartesian coordinates of ligands **9** and **11** docked into the active site of AChE
4. Cartesian coordinates of ligands **9** and **11** docked into the active site of BChE
5. Free energies of binding, the number of conformational clusters, and distribution of conformations obtained by molecular docking
6. Table S1. The mutagenic potential of new charged thienobenzo-1,2,3-triazolinium salts **1–15** through Lhasa M7 evaluation

1. ^{1}H and ^{13}C NMR spectra of new charged triazole salts 1–15

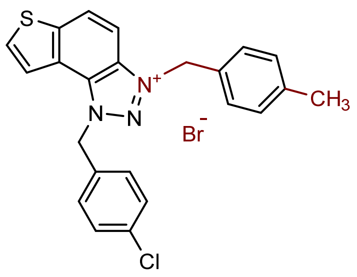

1

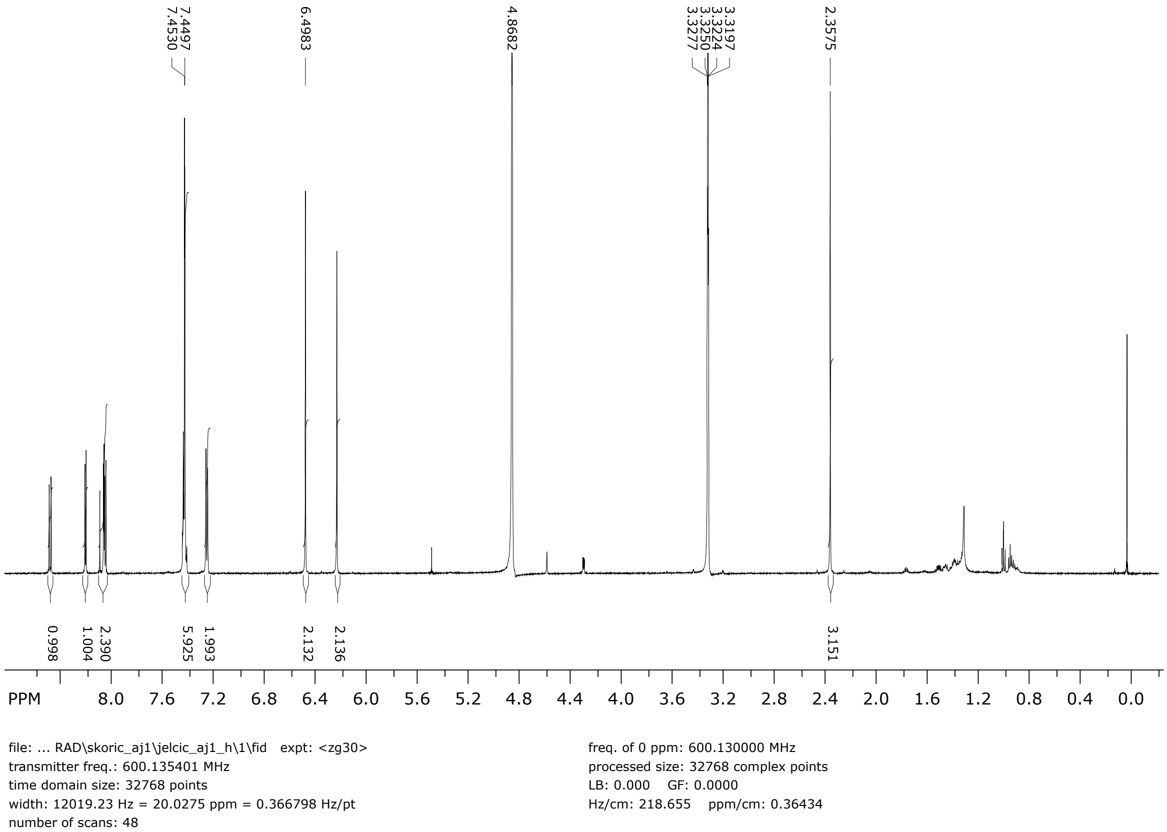

Figure S1. ^{1}H NMR spectrum (CD_3OD) of triazole salt **1**.

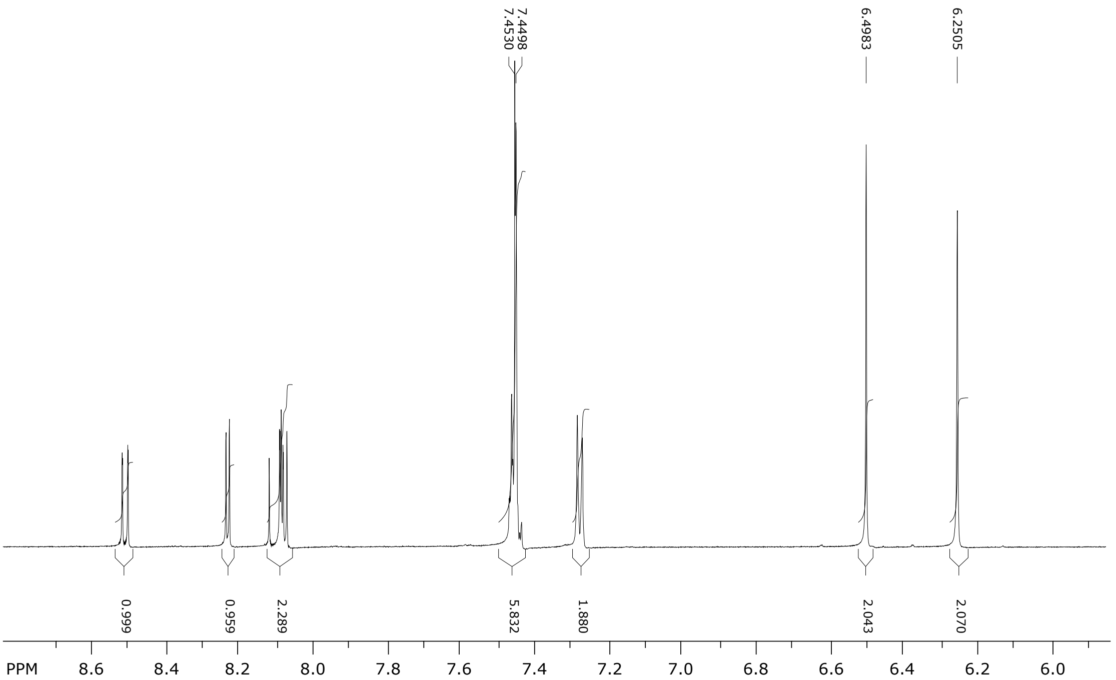

file: ... RAD\skoric_aj1\jelcic_aj1_h\1\fid expt: <zg30>
transmitter freq.: 600.135401 MHz
time domain size: 32768 points
width: 12019.23 Hz = 20.0275 ppm = 0.366798 Hz/pt
number of scans: 48

freq. of 0 ppm: 600.130000 MHz
processed size: 32768 complex points
LB: 0.000 GF: 0.0000
Hz/cm: 71.980 ppm/cm: 0.11994

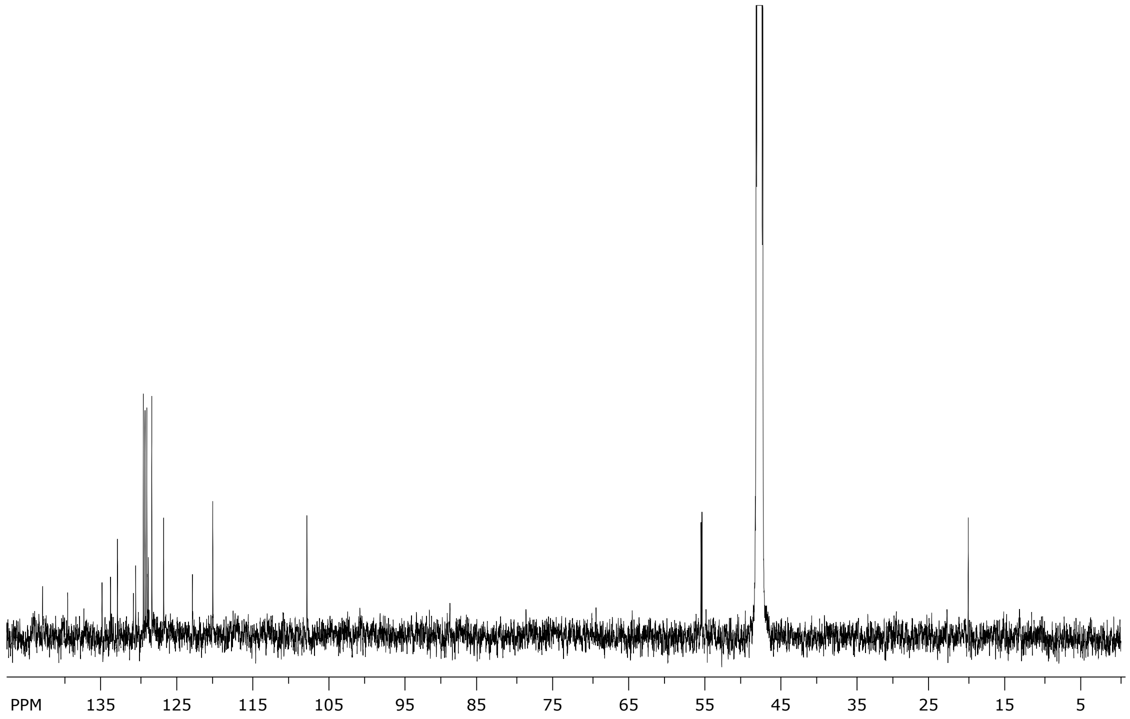

file: ...o sada za RAD\jelcic_aj1_13c\1\fid expt: <zgpg30>
transmitter freq.: 150.917899 MHz
time domain size: 65536 points
width: 35971.22 Hz = 238.3496 ppm = 0.548877 Hz/pt
number of scans: 8192

freq. of 0 ppm: 150.902809 MHz
processed size: 32768 complex points
LB: 0.000 GF: 0.0000
Hz/cm: 895.957 ppm/cm: 5.93672

Figure S2. ^{13}C NMR spectrum (CD_3OD) of triazole salt **1**.

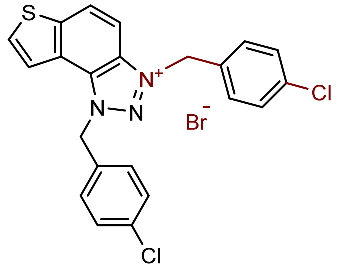

2

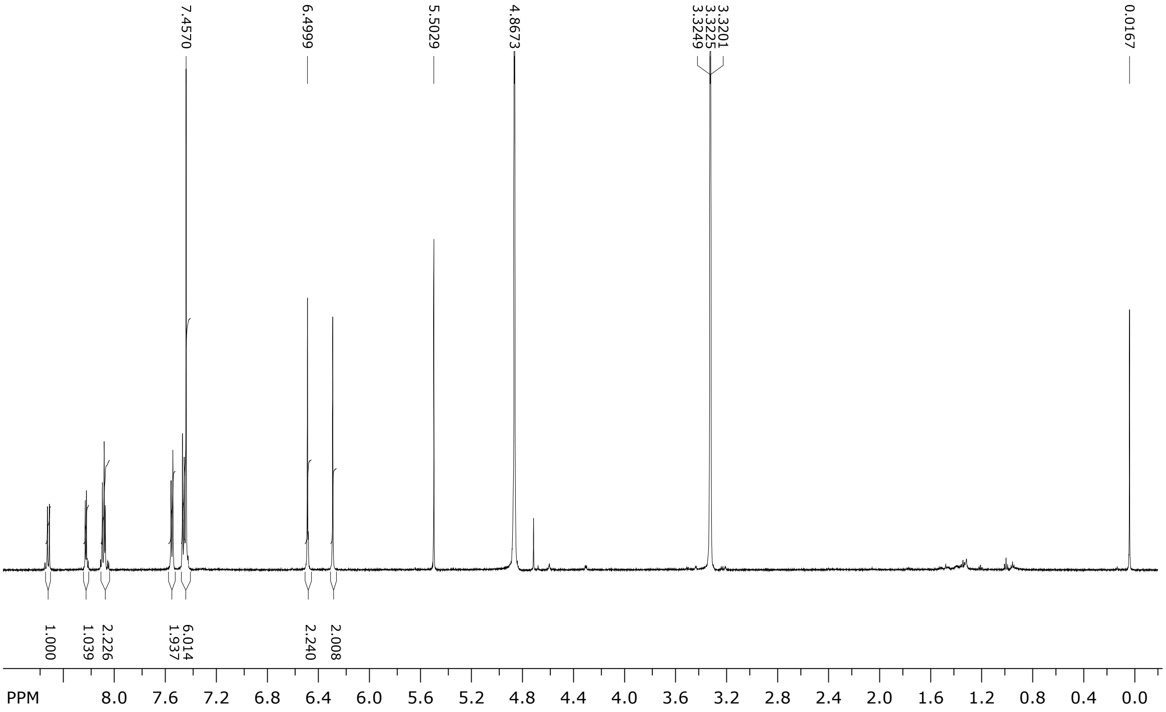

file: ...no do sada za RAD\jelcic_aj9\1\fid expt: <zg30>
transmitter freq.: 600.135401 MHz
time domain size: 32768 points
width: 12019.23 Hz = 20.0275 ppm = 0.366798 Hz/pt
number of scans: 32

freq. of 0 ppm: 600.130000 MHz
processed size: 32768 complex points
LB: 0.000 GF: 0.0000
Hz/cm: 218.655 ppm/cm: 0.36434

Figure S3. ^{1}H NMR spectrum (CD_3OD) of triazole salt **2**.

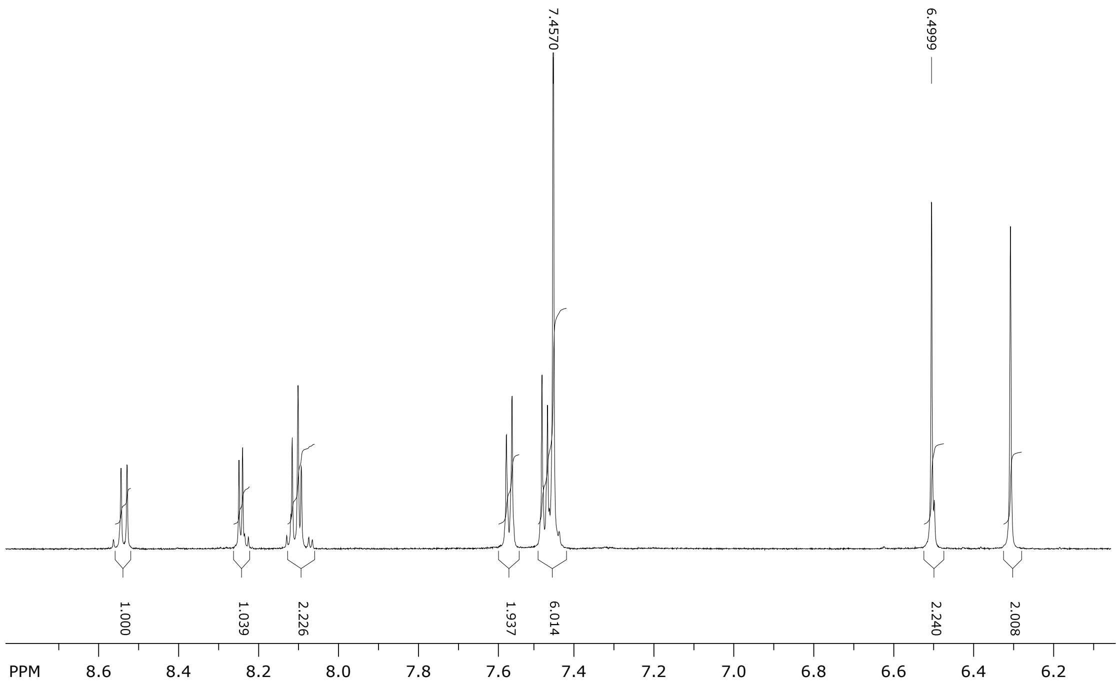

file: ...no do sada za RAD\jelcic_aj9\1\fid expt: <zg30>
transmitter freq.: 600.135401 MHz
time domain size: 32768 points
width: 12019.23 Hz = 20.0275 ppm = 0.366798 Hz/pt
number of scans: 32

freq. of 0 ppm: 600.130000 MHz
processed size: 32768 complex points
LB: 0.000 GF: 0.0000
Hz/cm: 67.172 ppm/cm: 0.11193

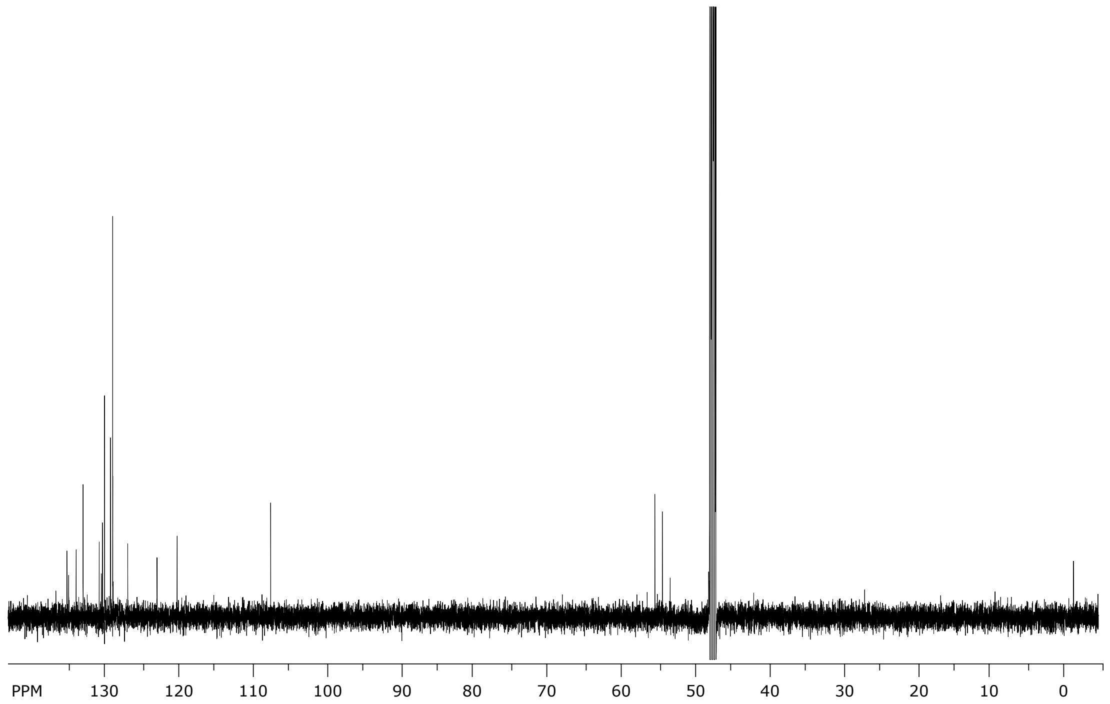

file: ...no do sada za RAD\jelcic_aj9\2\fid expt: <zgpg30>
transmitter freq.: 150.917899 MHz
time domain size: 65536 points
width: 35971.22 Hz = 238.3496 ppm = 0.548877 Hz/pt
number of scans: 4340

freq. of 0 ppm: 150.902809 MHz
processed size: 32768 complex points
LB: 0.000 GF: 0.0000
Hz/cm: 895.216 ppm/cm: 5.93181

Figure S4. ^{13}C NMR spectrum (CD_3OD) of triazole salt **2**.

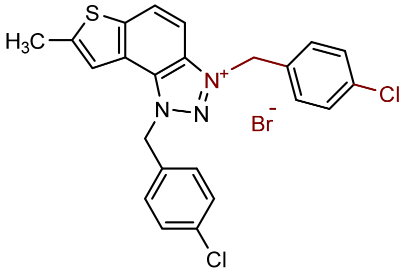

3

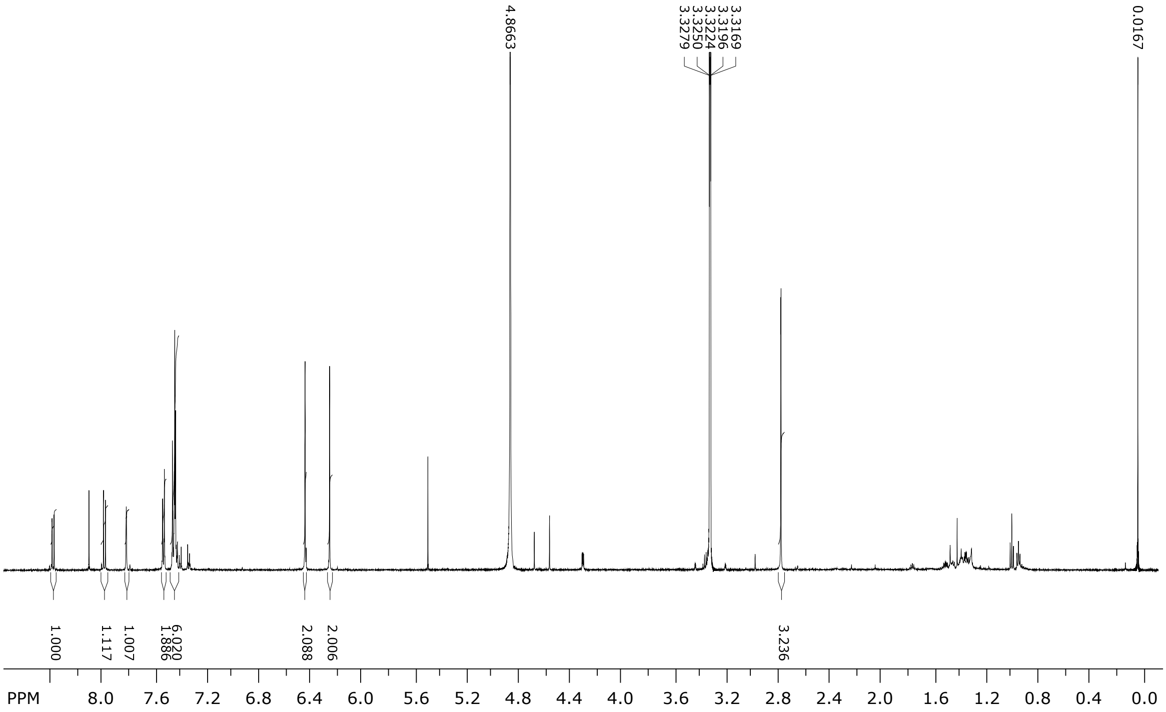

file: ...imljeno do sada za RAD\aj11_1H\fid expt: <zg30>
transmitter freq.: 600.134201 MHz
time domain size: 32768 points
width: 12019.23 Hz = 20.0276 ppm = 0.366798 Hz/pt
number of scans: 64

freq. of 0 ppm: 600.130000 MHz
processed size: 32768 complex points
LB: 0.000 GF: 0.0000
Hz/cm: 214.241 ppm/cm: 0.35699

Figure S5. ^1H NMR spectrum (CD_3OD) of triazole salt **3**.

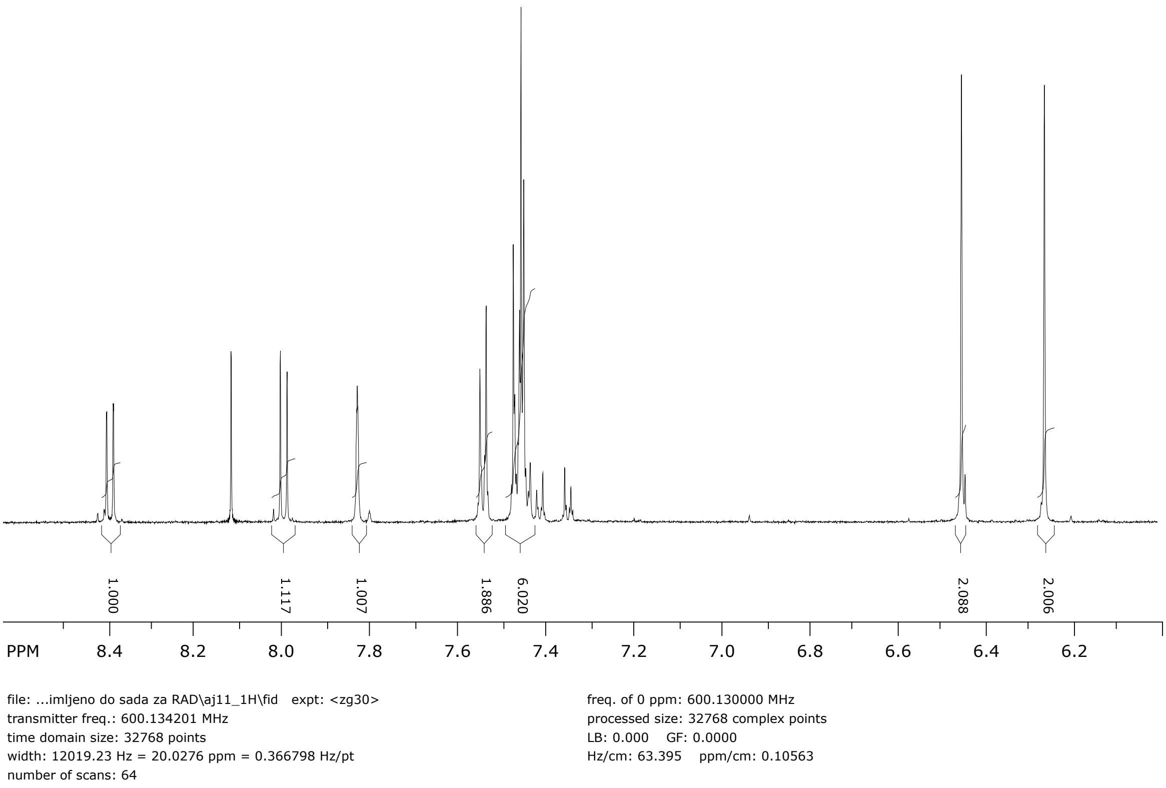

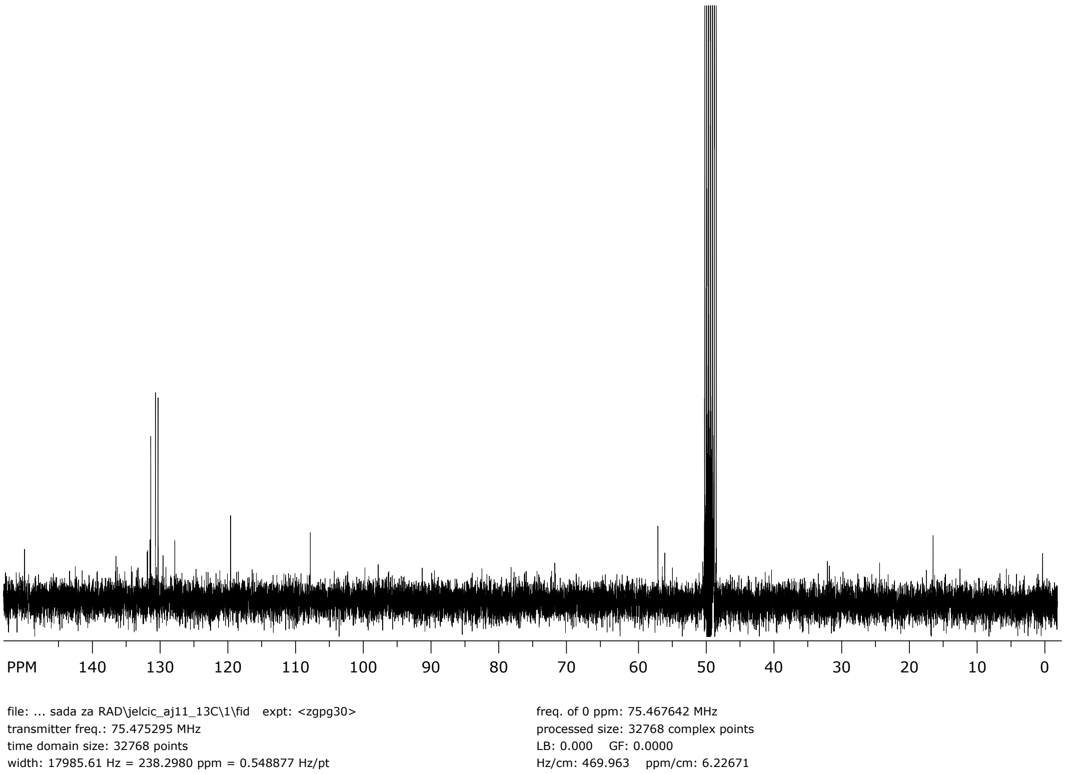

Figure S6. ^{13}C NMR spectrum (CD_3OD) of triazole salt **3**.

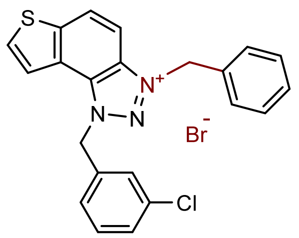

4

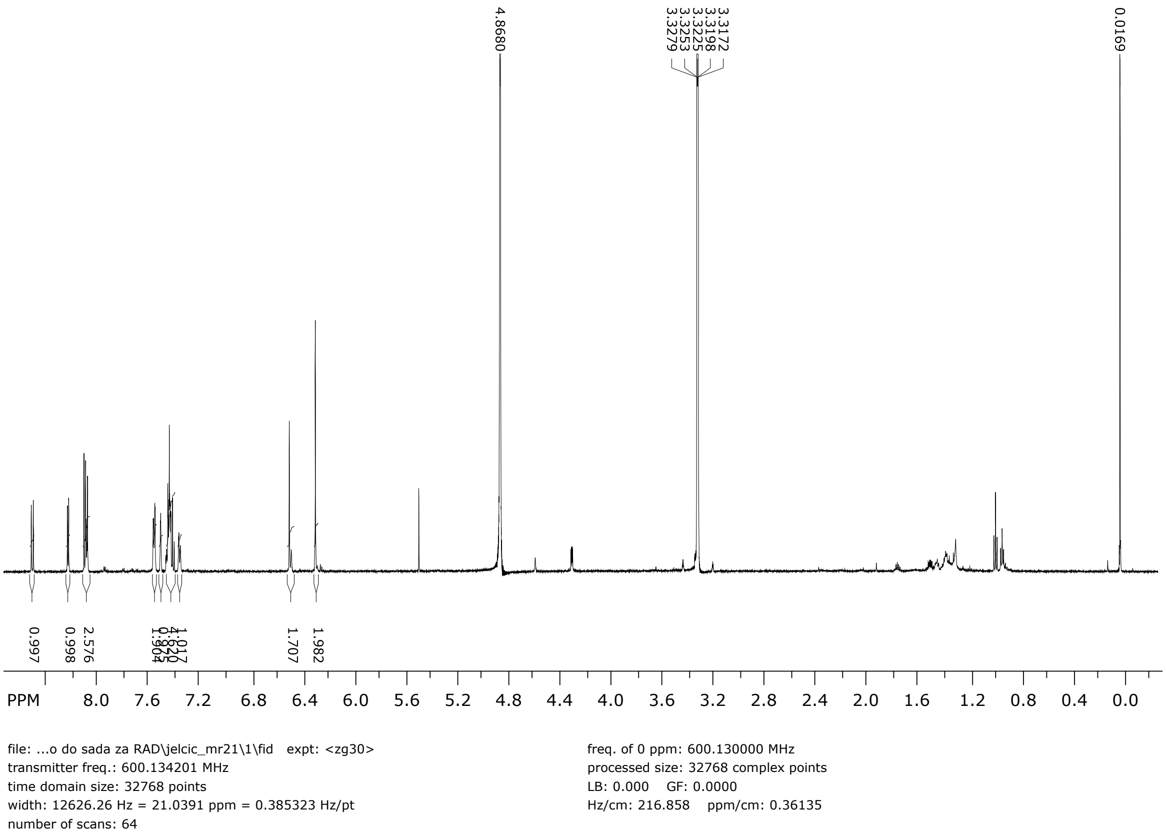

Figure S7. ^{1}H NMR spectrum (CD_3OD) of triazole salt **4**.

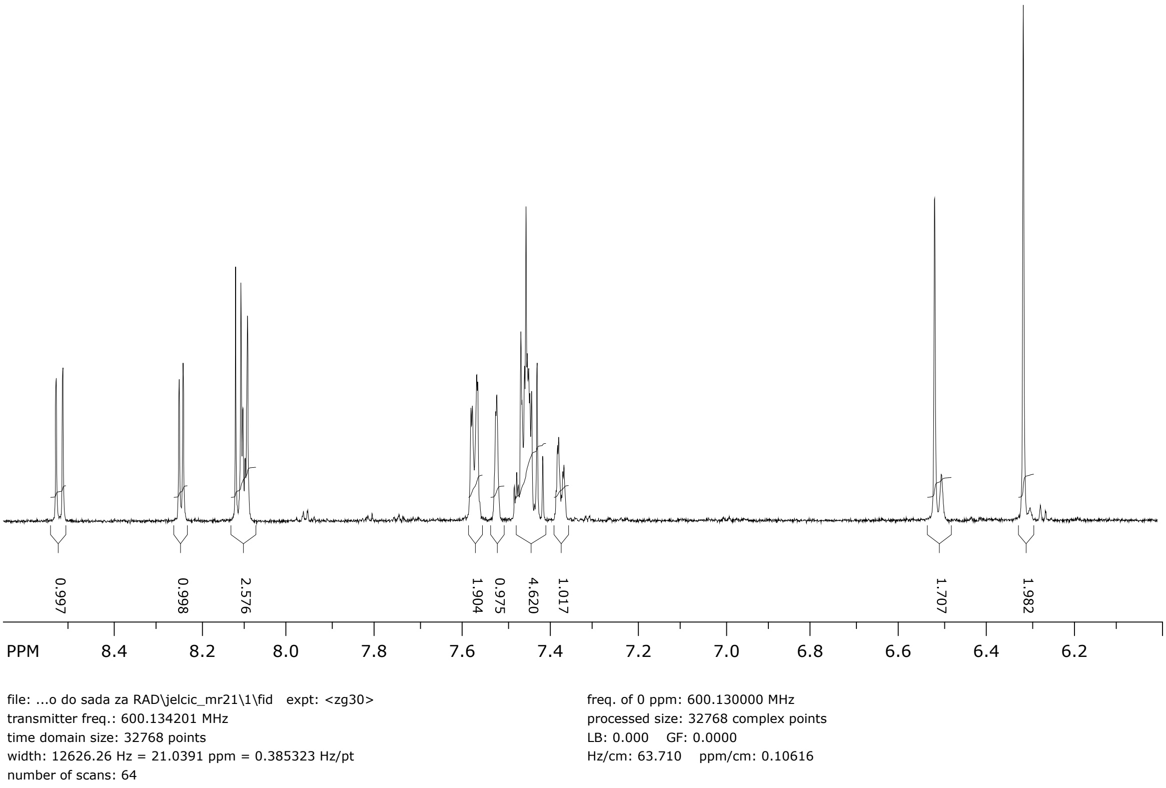

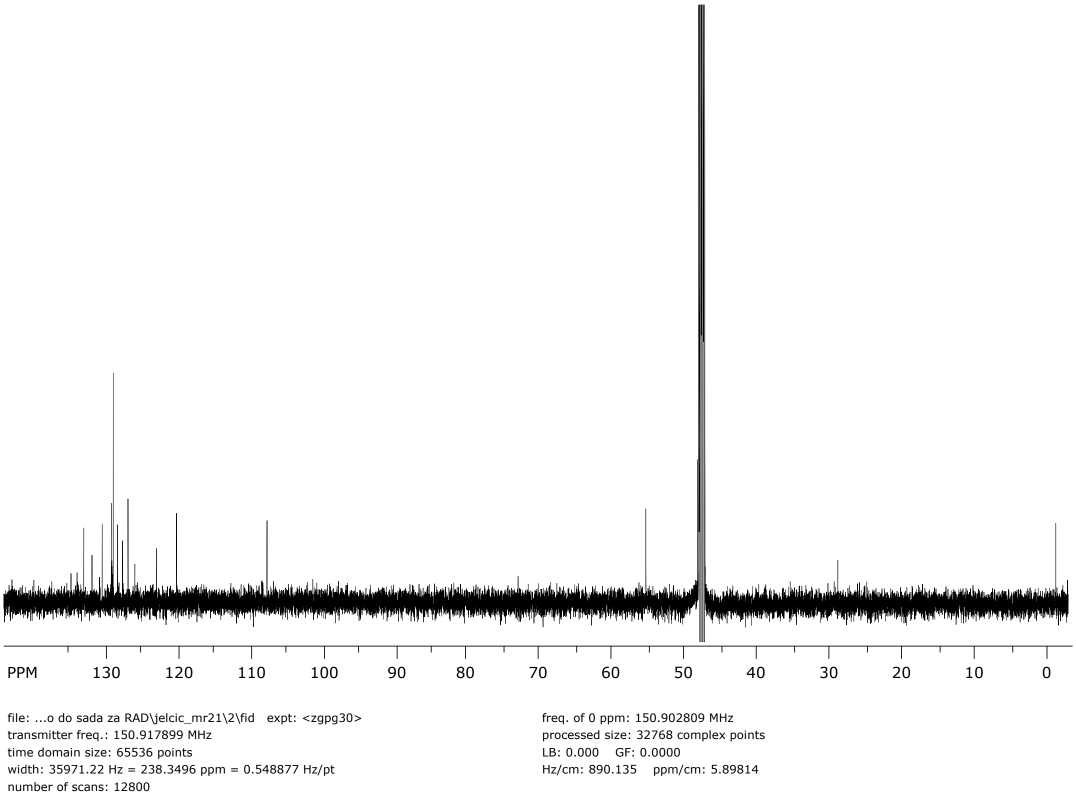

Figure S8. ^{13}C NMR spectrum (CD$_3$OD) of triazole salt **4**.

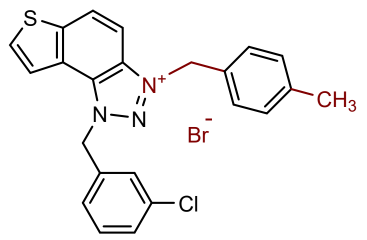

5

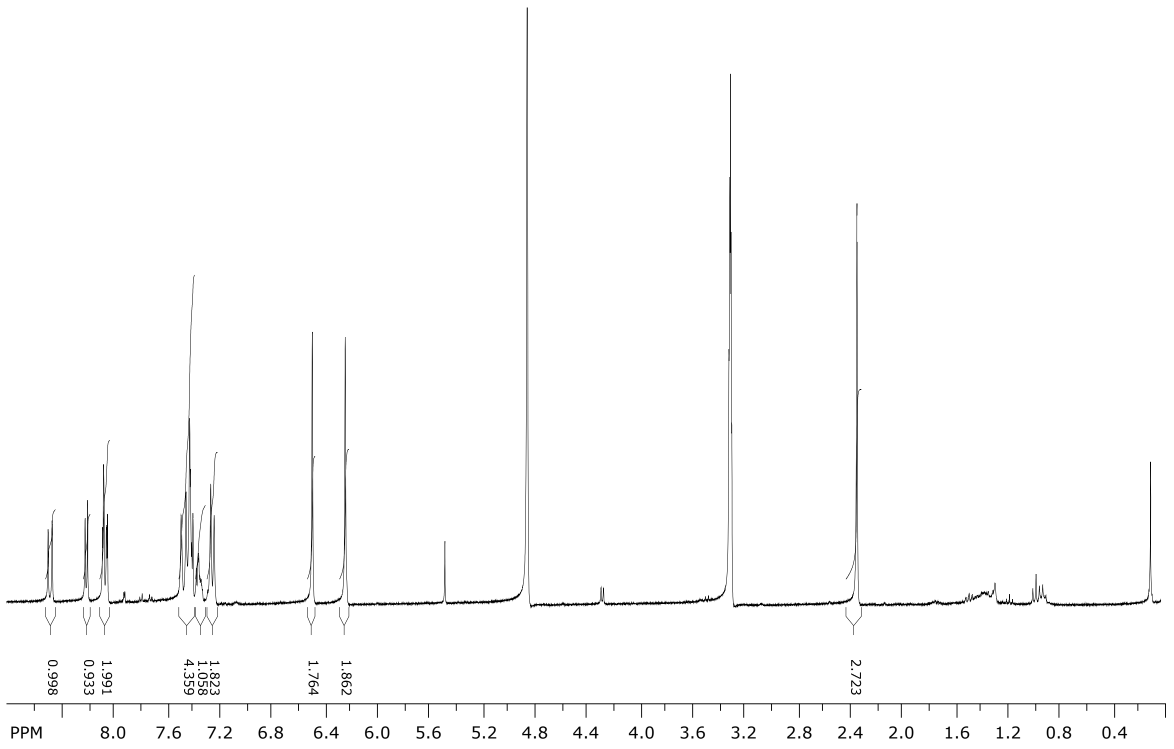

file: ... RAD\jelcic_mr22\skoric_mr22\1\fid expt: <zg30>
transmitter freq.: 300.132701 MHz
time domain size: 32768 points
width: 6172.84 Hz = 20.5670 ppm = 0.188380 Hz/pt
number of scans: 64

freq. of 0 ppm: 300.130006 MHz
processed size: 32768 complex points
LB: 0.000 GF: 0.0000
Hz/cm: 105.845 ppm/cm: 0.35266

Figure S9. ^{1}H NMR spectrum (CD_3OD) of triazole salt **5**.

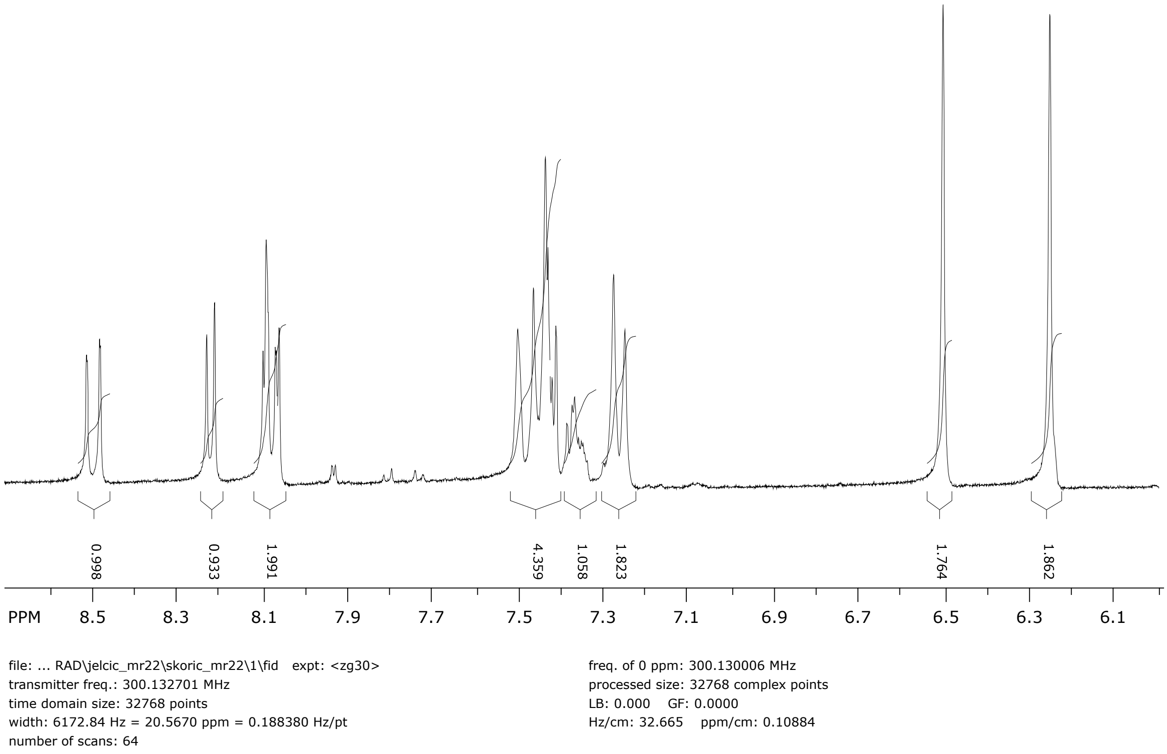

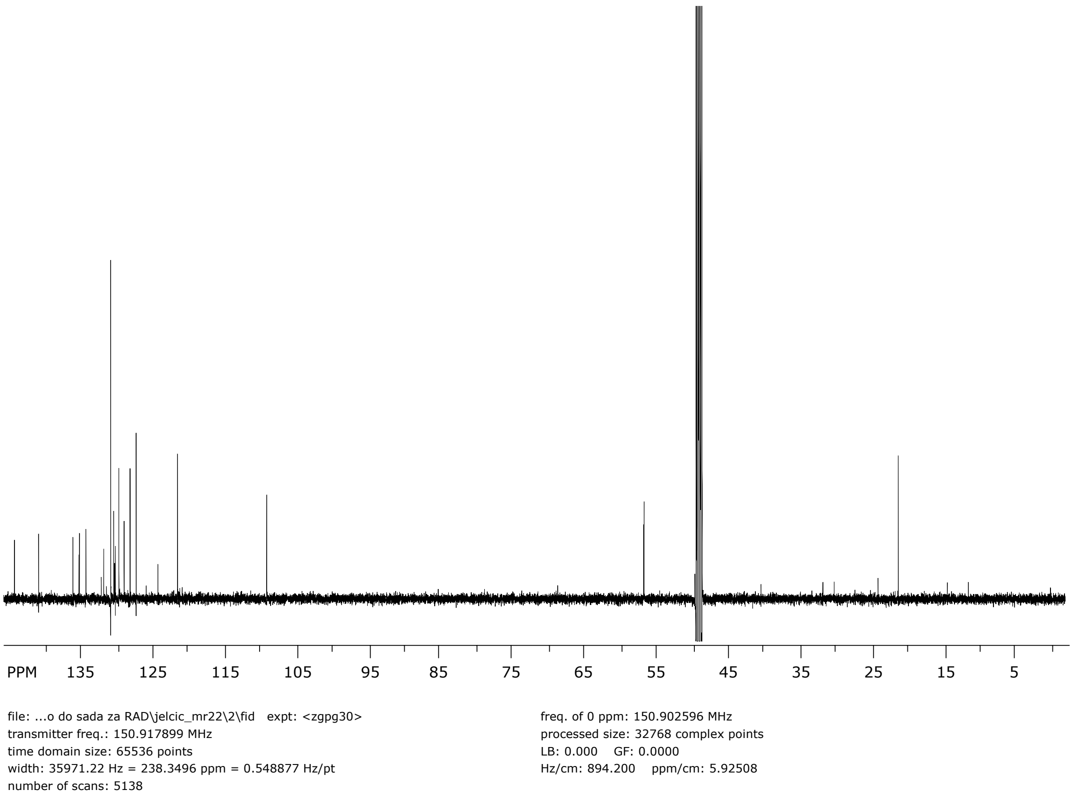

Figure S10. ^{13}C NMR spectrum (CD_3OD) of triazole salt **5**.

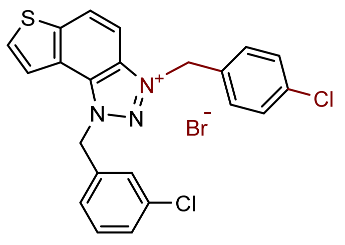

6

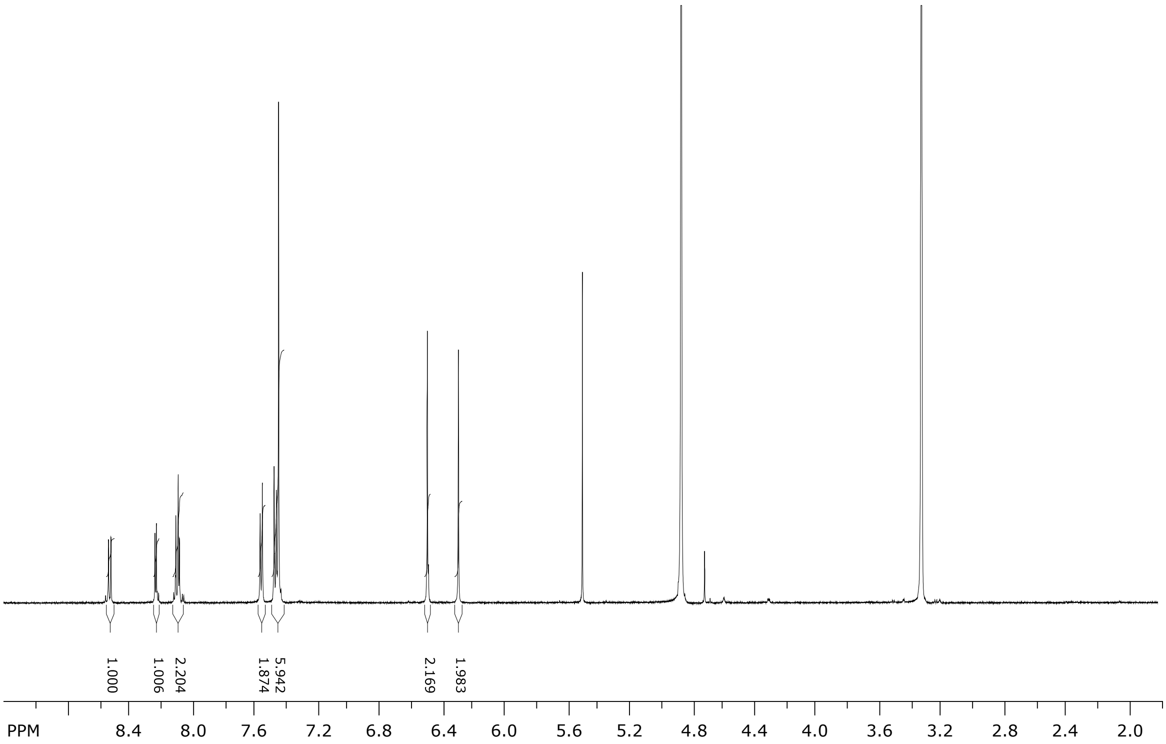

file: ...no do sada za RAD\jelcic_aj9\1\fid expt: <zg30>
transmitter freq.: 600.135401 MHz
time domain size: 32768 points
width: 12019.23 Hz = 20.0275 ppm = 0.366798 Hz/pt
number of scans: 32

freq. of 0 ppm: 600.130000 MHz
processed size: 32768 complex points
LB: 0.000 GF: 0.0000
Hz/cm: 178.251 ppm/cm: 0.29702

Figure S11. ^{1}H NMR spectrum (CD_3OD) of triazole salt **6**.

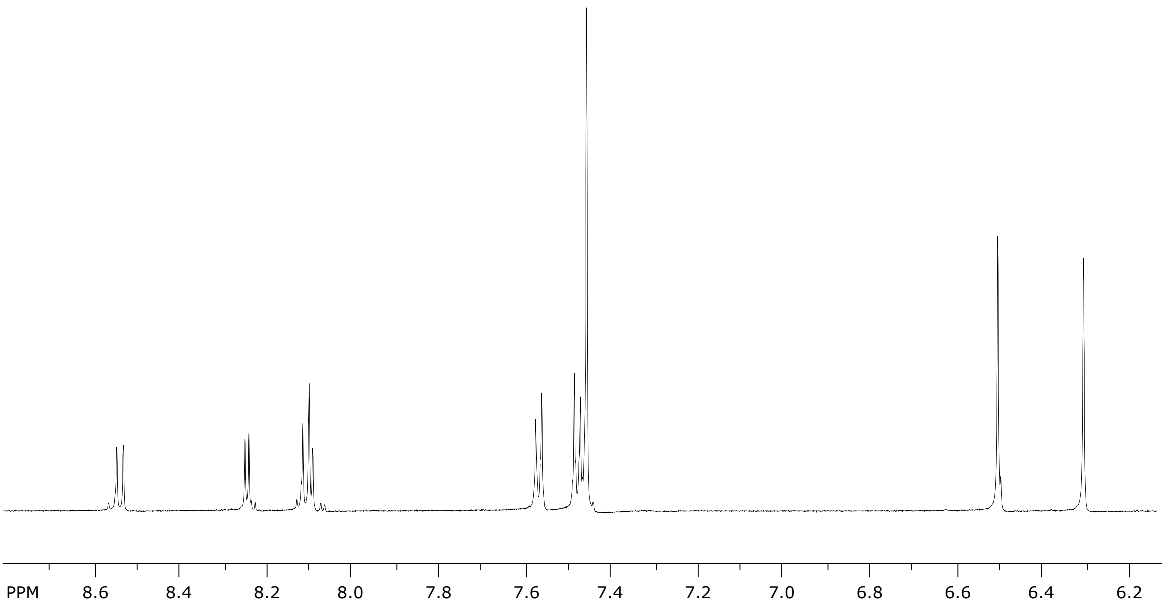

file: ...no do sada za RAD\jelcic_aj9\1\fid expt: <zg30>
transmitter freq.: 600.135401 MHz
time domain size: 32768 points
width: 12019.23 Hz = 20.0275 ppm = 0.366798 Hz/pt
number of scans: 32

freq. of 0 ppm: 600.130000 MHz
processed size: 32768 complex points
LB: 0.000 GF: 0.0000
Hz/cm: 64.510 ppm/cm: 0.10749

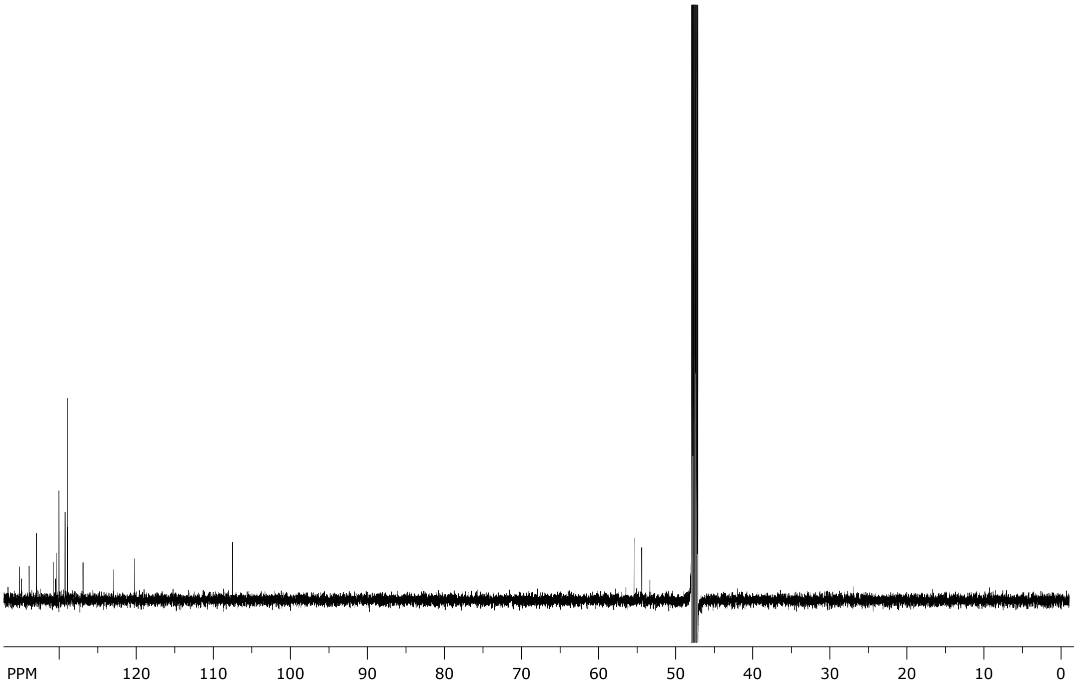

file: ...no do sada za RAD\jelcic_aj9\2\fid expt: <zgpg30>
transmitter freq.: 150.917899 MHz
time domain size: 65536 points
width: 35971.22 Hz = 238.3496 ppm = 0.548877 Hz/pt
number of scans: 4340

freq. of 0 ppm: 150.902809 MHz
processed size: 32768 complex points
LB: 0.000 GF: 0.0000
Hz/cm: 837.296 ppm/cm: 5.54802

Figure S12. ^{13}C NMR spectrum (CD_3OD) of triazole salt **6**.

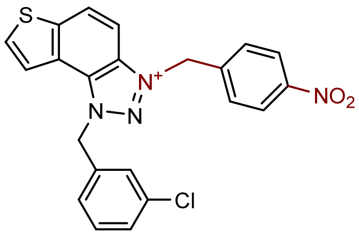

7

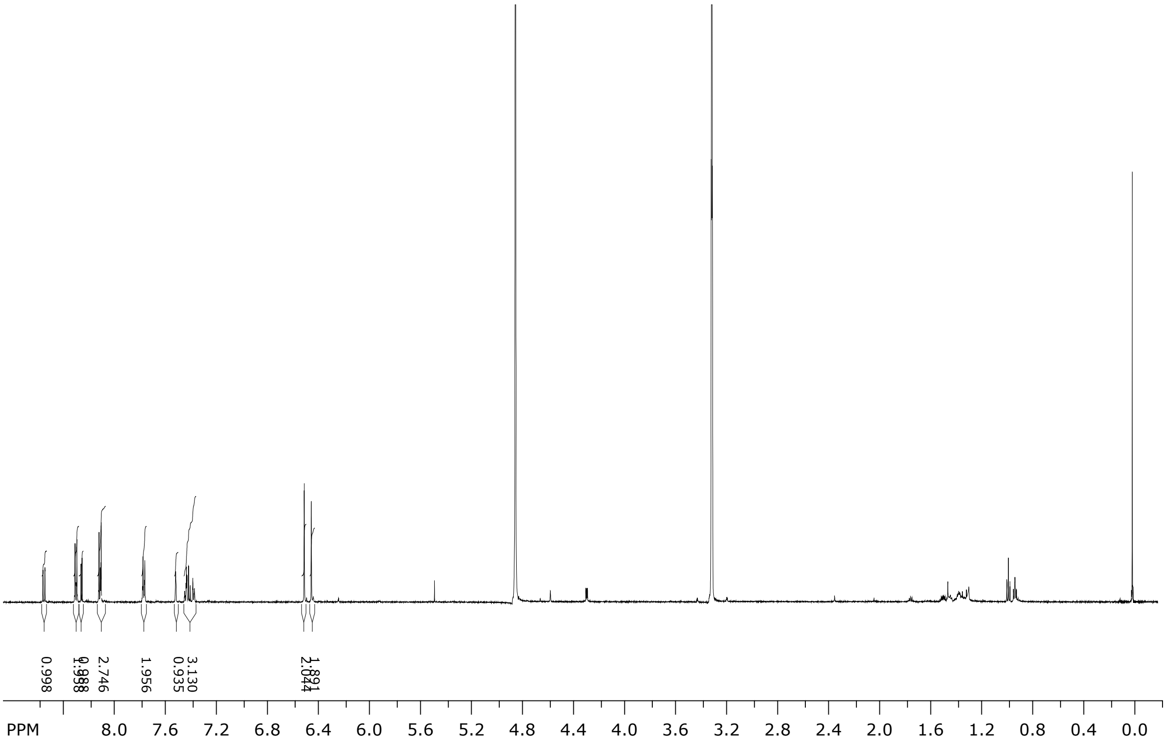

file: ...imljeno do sada za RAD\aj10_1H\fid expt: <zg30>
transmitter freq.: 600.134201 MHz
time domain size: 32768 points
width: 12019.23 Hz = 20.0276 ppm = 0.366798 Hz/pt
number of scans: 64

freq. of 0 ppm: 600.130000 MHz
processed size: 32768 complex points
LB: 0.000 GF: 0.0000
Hz/cm: 217.976 ppm/cm: 0.36321

Figure S13. ^{1}H NMR spectrum (CD_3OD) of triazole salt **7**.

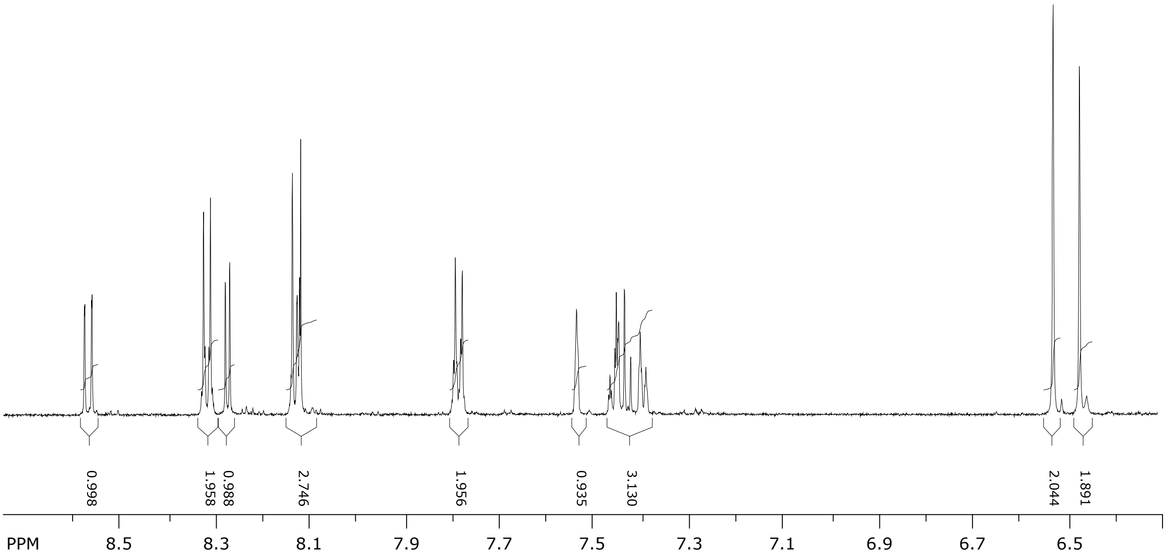

file: ...imljeno do sada za RAD\aj10_1H\fid expt: <zg30>
transmitter freq.: 600.134201 MHz
time domain size: 32768 points
width: 12019.23 Hz = 20.0276 ppm = 0.366798 Hz/pt
number of scans: 64

freq. of 0 ppm: 600.130000 MHz
processed size: 32768 complex points
LB: 0.000 GF: 0.0000
Hz/cm: 58.804 ppm/cm: 0.09799

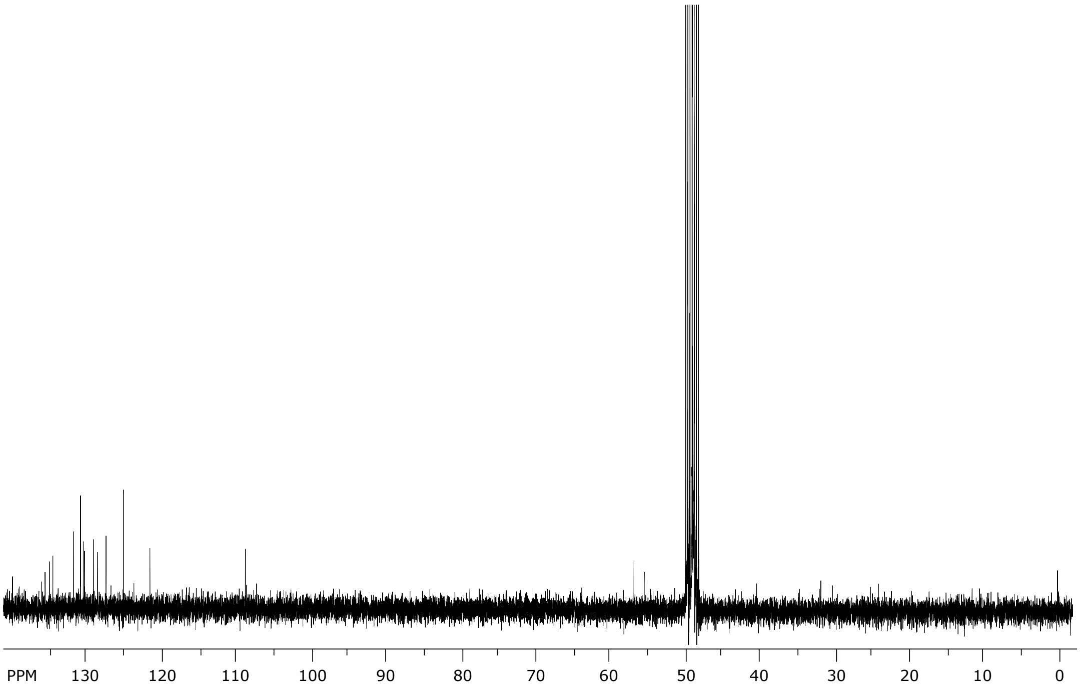

file: ... sada za RAD\jelcic_aj10_13C\1\fid expt: <zgpg30>
transmitter freq.: 75.475295 MHz
time domain size: 32768 points
width: 17985.61 Hz = 238.2980 ppm = 0.548877 Hz/pt
number of scans: 32000

freq. of 0 ppm: 75.467642 MHz
processed size: 32768 complex points
LB: 0.000 GF: 0.0000
Hz/cm: 433.382 ppm/cm: 5.74204

Figure S14. ^{13}C NMR spectrum (CD_3OD) of triazole salt **7**.

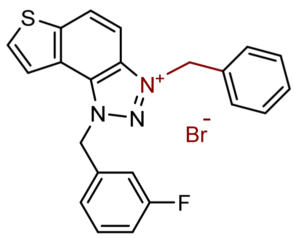

8

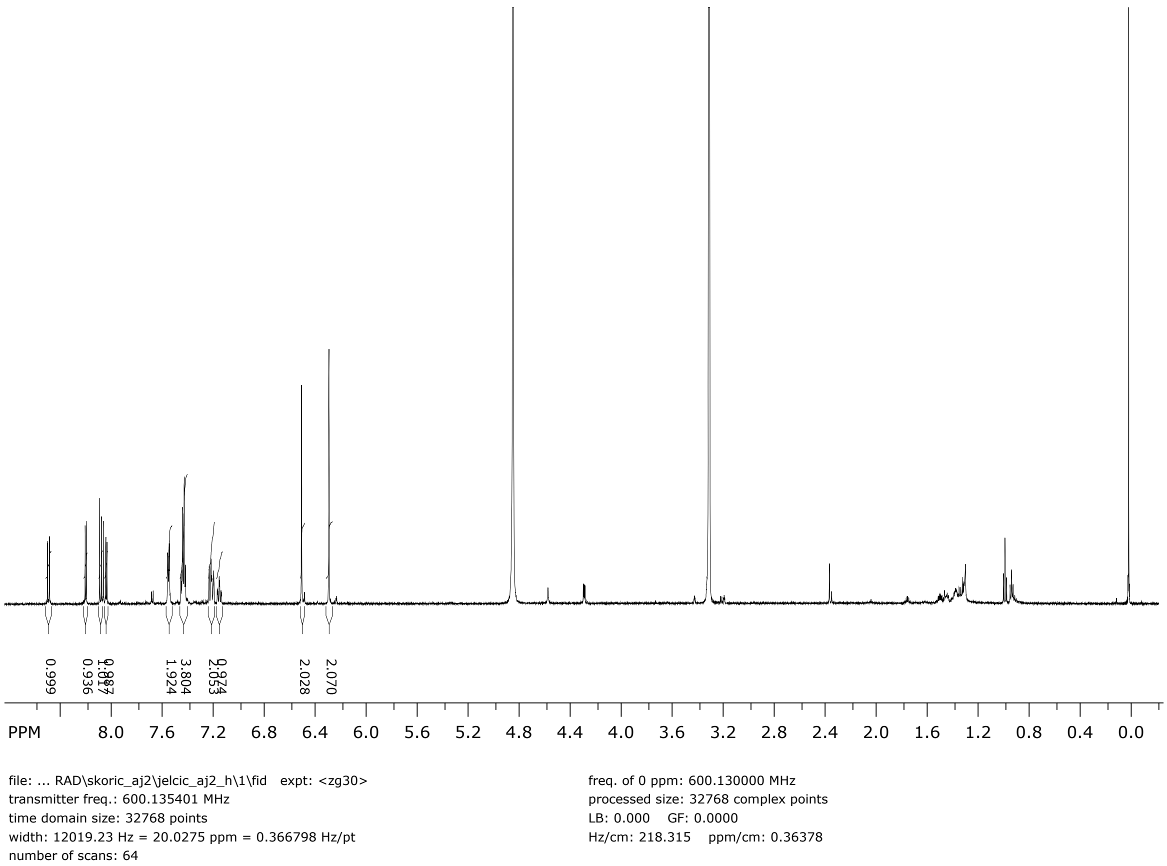

Figure S15. ^{1}H NMR spectrum (CD_3OD) of triazole salt **8**.

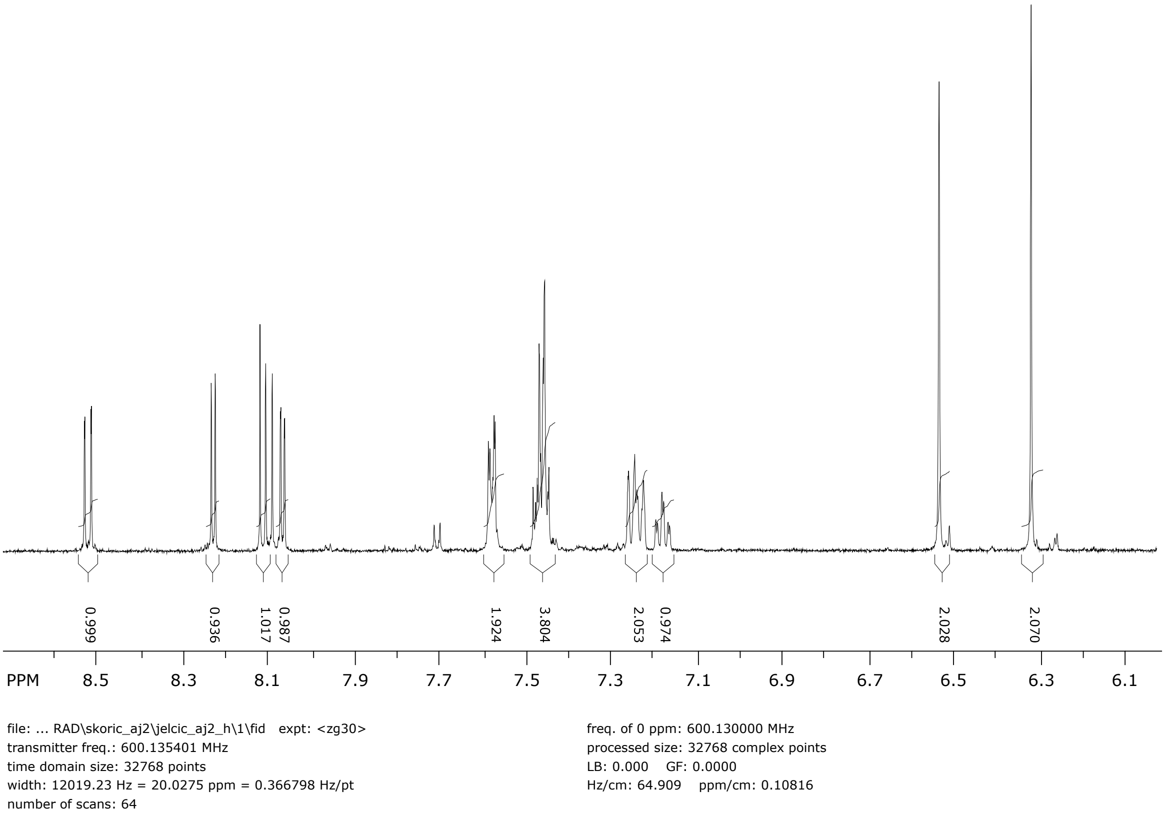

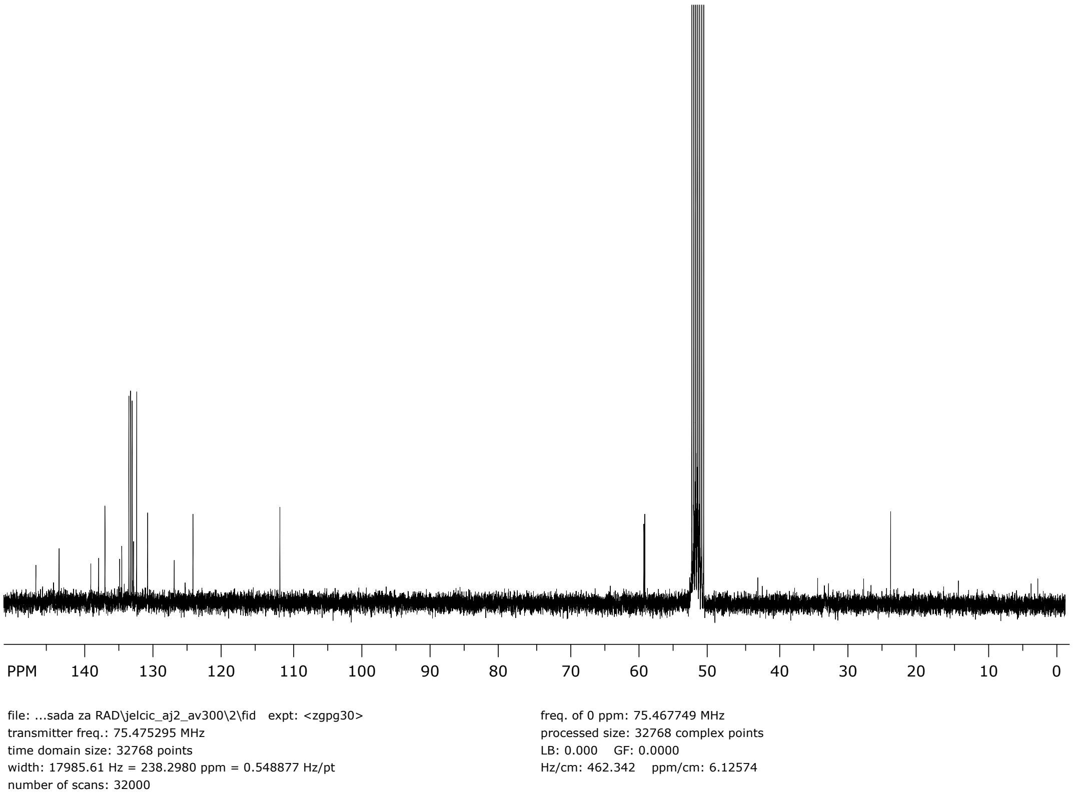

Figure S16. ${}^{13}C$ NMR spectrum (CD_3OD) of triazole salt **8**.

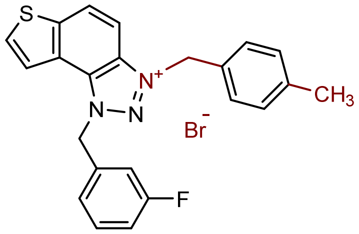

9

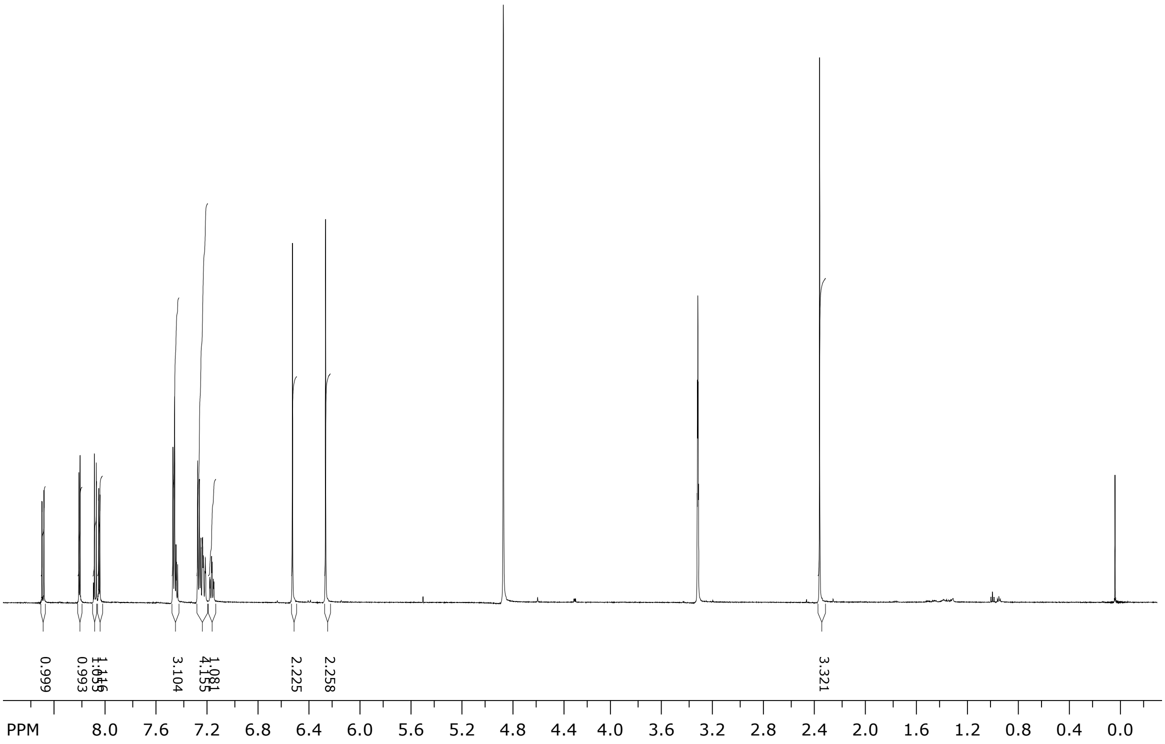

file: ... RAD\skoric_aj3\jelcic_aj3_h\1\fid expt: <zg30>
transmitter freq.: 600.135401 MHz
time domain size: 32768 points
width: 12019.23 Hz = 20.0275 ppm = 0.366798 Hz/pt
number of scans: 21

freq. of 0 ppm: 600.130000 MHz
processed size: 32768 complex points
LB: 0.000 GF: 0.0000
Hz/cm: 219.674 ppm/cm: 0.36604

Figure S17. ^{1}H NMR spectrum (CD_3OD) of triazole salt **9**.

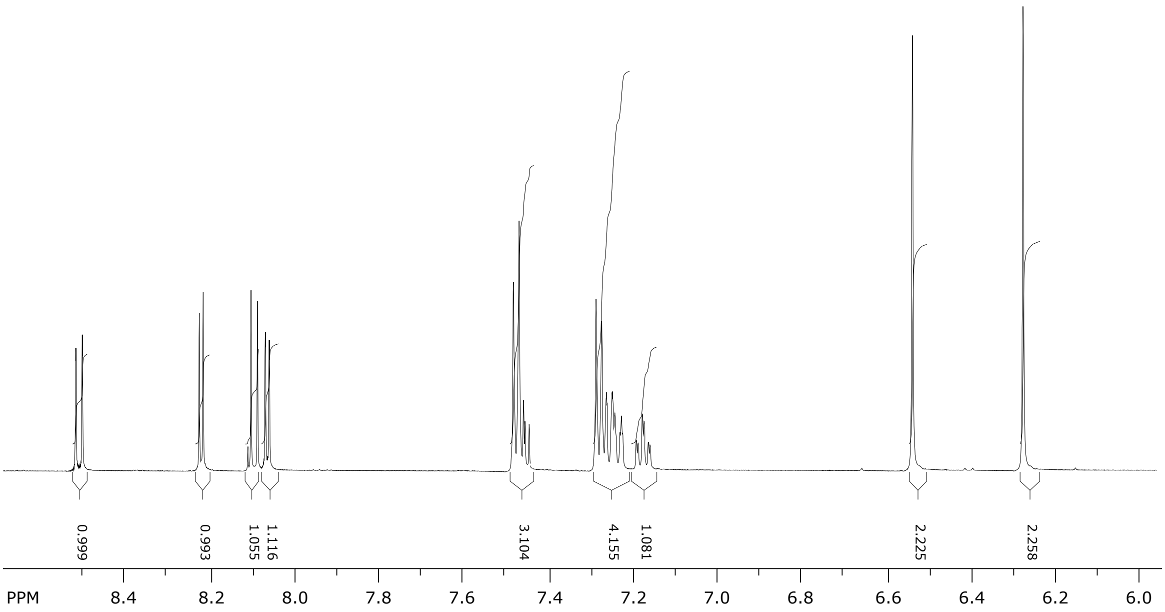

file: ... RAD\skoric_aj3\jelcic_aj3_h\1\fid expt: <zg30>
transmitter freq.: 600.135401 MHz
time domain size: 32768 points
width: 12019.23 Hz = 20.0275 ppm = 0.366798 Hz/pt
number of scans: 21

freq. of 0 ppm: 600.130000 MHz
processed size: 32768 complex points
LB: 0.000 GF: 0.0000
Hz/cm: 65.778 ppm/cm: 0.10961

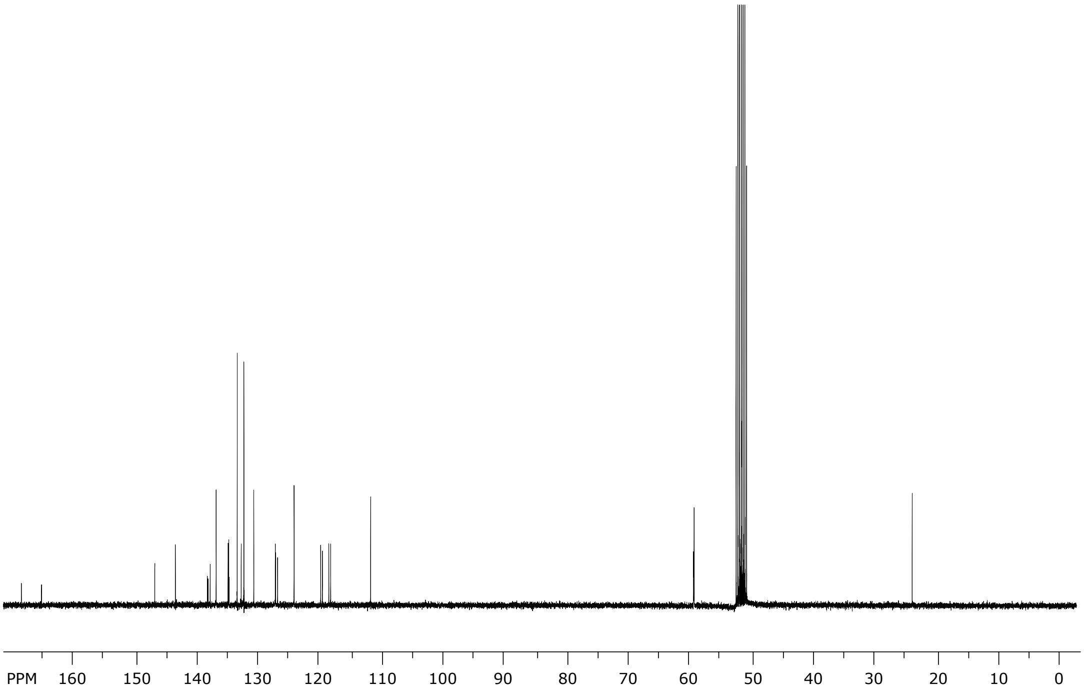

file: ...sada za RAD\jelcic_aj3_av300\2\fid expt: <zgpg30>
transmitter freq.: 75.475295 MHz
time domain size: 32768 points
width: 17985.61 Hz = 238.2980 ppm = 0.548877 Hz/pt
number of scans: 31394

freq. of 0 ppm: 75.467749 MHz
processed size: 32768 complex points
LB: 0.000 GF: 0.0000
Hz/cm: 527.375 ppm/cm: 6.98738

Figure S18. ^{13}C NMR spectrum (CD_3OD) of triazole salt **9**.

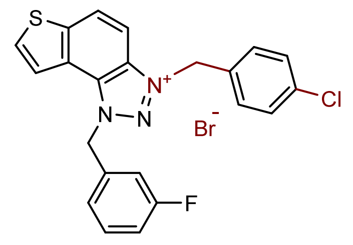

10

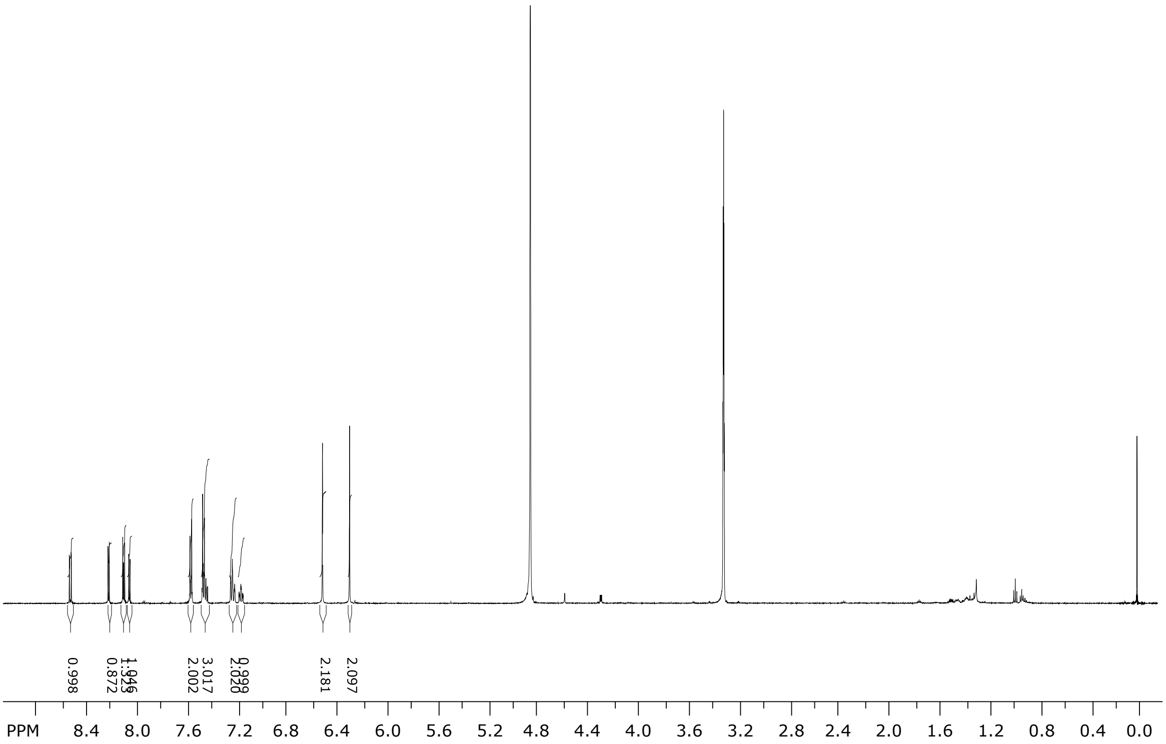

file: ...za RAD\skoric_aj4\jelcic_aj4\1\fid expt: <zg30>
transmitter freq.: 600.135401 MHz
time domain size: 32768 points
width: 12019.23 Hz = 20.0275 ppm = 0.366798 Hz/pt
number of scans: 32

freq. of 0 ppm: 600.130000 MHz
processed size: 32768 complex points
LB: 0.000 GF: 0.0000
Hz/cm: 221.711 ppm/cm: 0.36943

Figure S19. ^{1}H NMR spectrum (CD_3OD) of triazole salt **10**.

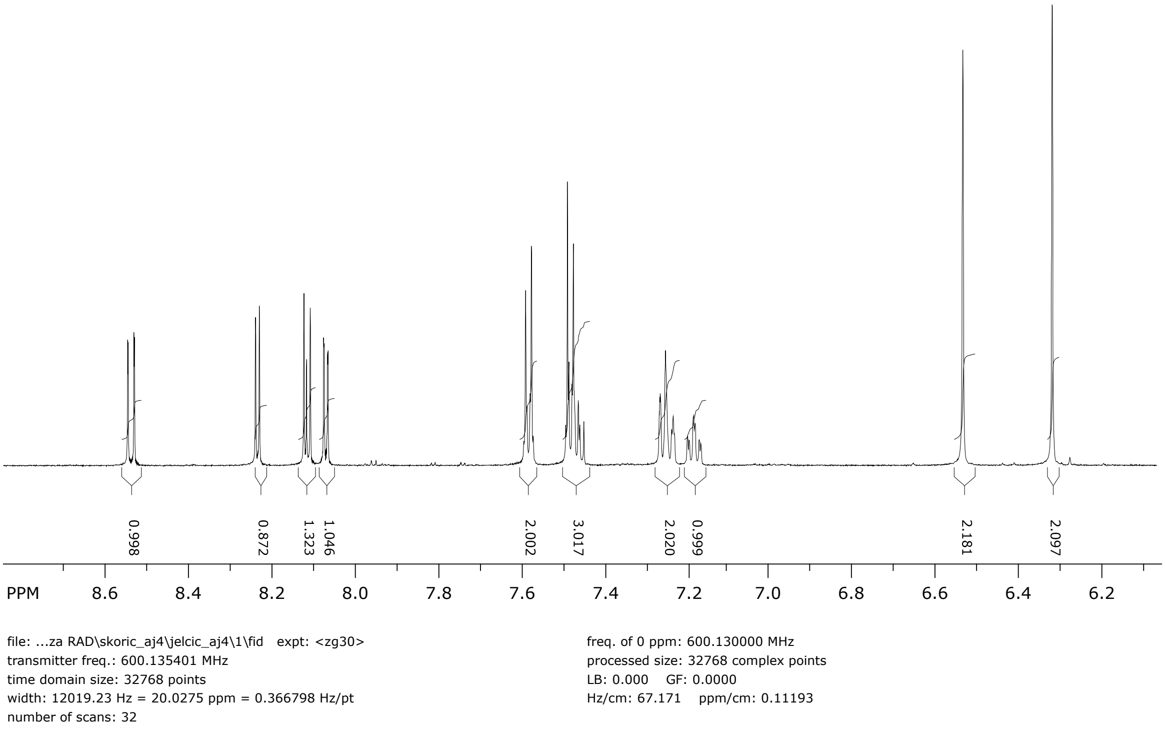

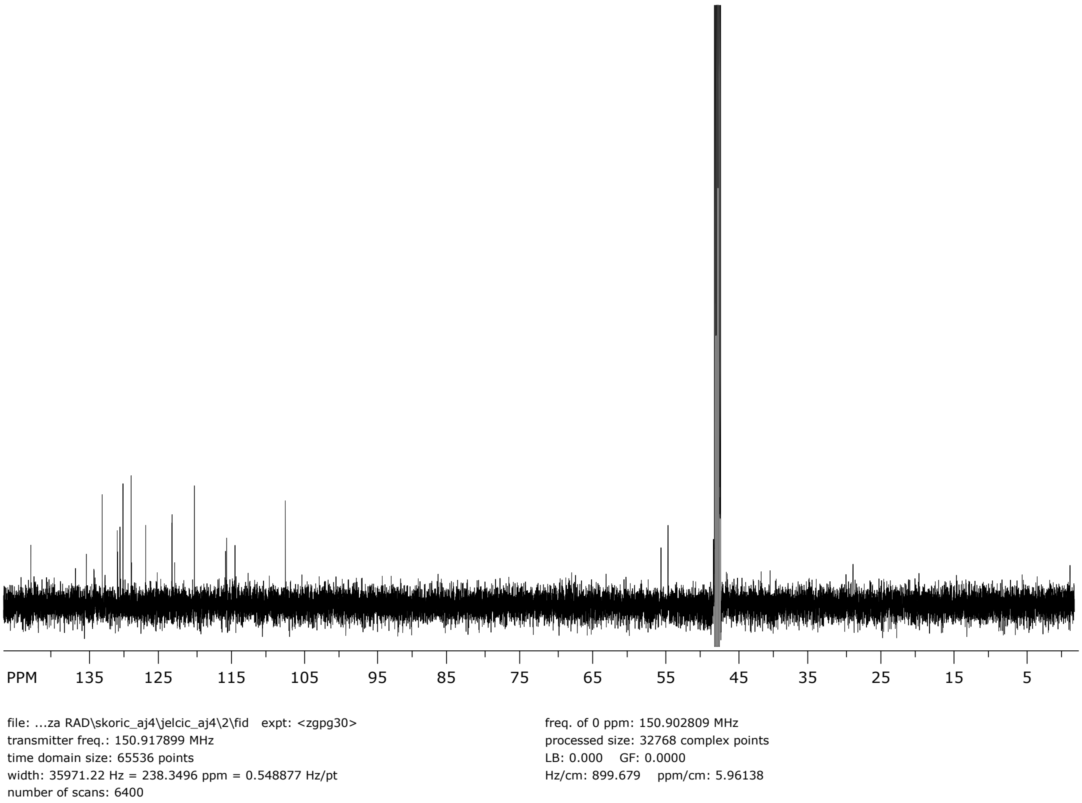

Figure S20. ^{13}C NMR spectrum (CD$_3$OD) of triazole salt **10**.

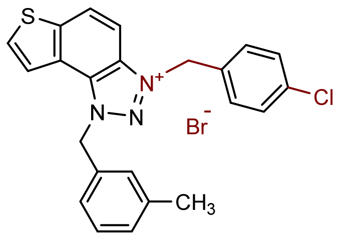

11

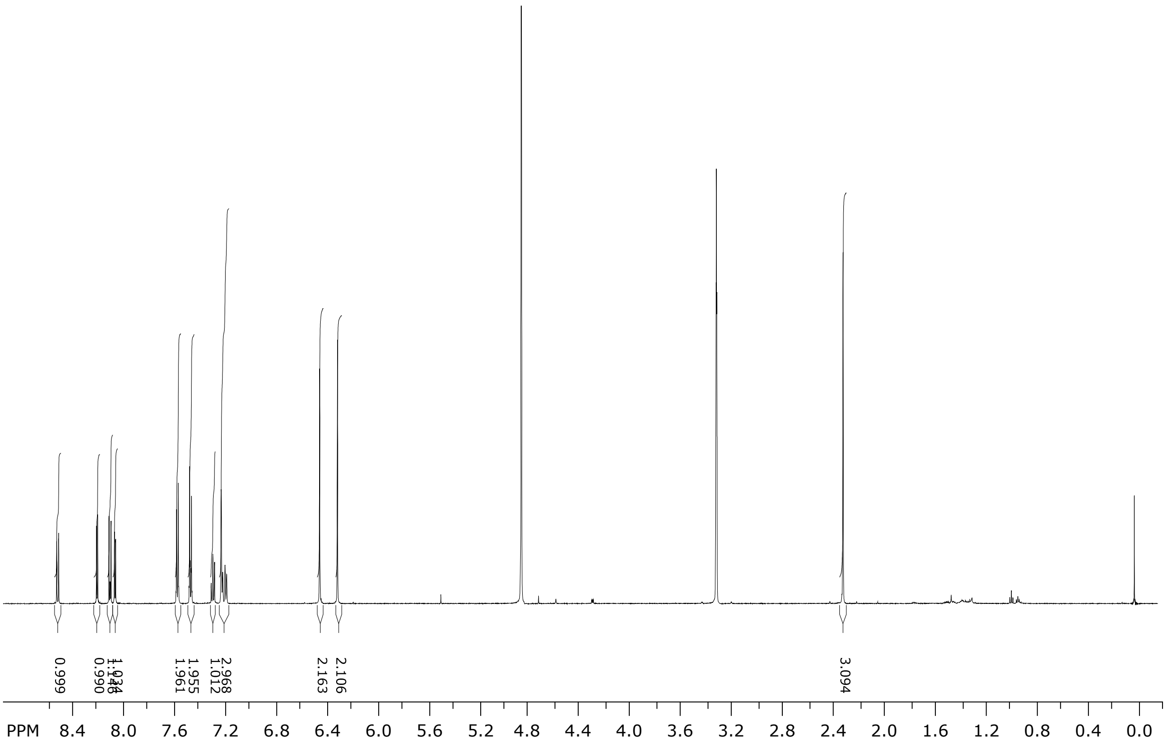

file: ...imljeno do sada za RAD\aj12_1H\fid expt: <zg30>
transmitter freq.: 600.134201 MHz
time domain size: 32768 points
width: 12019.23 Hz = 20.0276 ppm = 0.366798 Hz/pt
number of scans: 64

freq. of 0 ppm: 600.130000 MHz
processed size: 32768 complex points
LB: 0.000 GF: 0.0000
Hz/cm: 219.334 ppm/cm: 0.36547

Figure S21. ^{1}H NMR spectrum (CD_3OD) of triazole salt **11**.

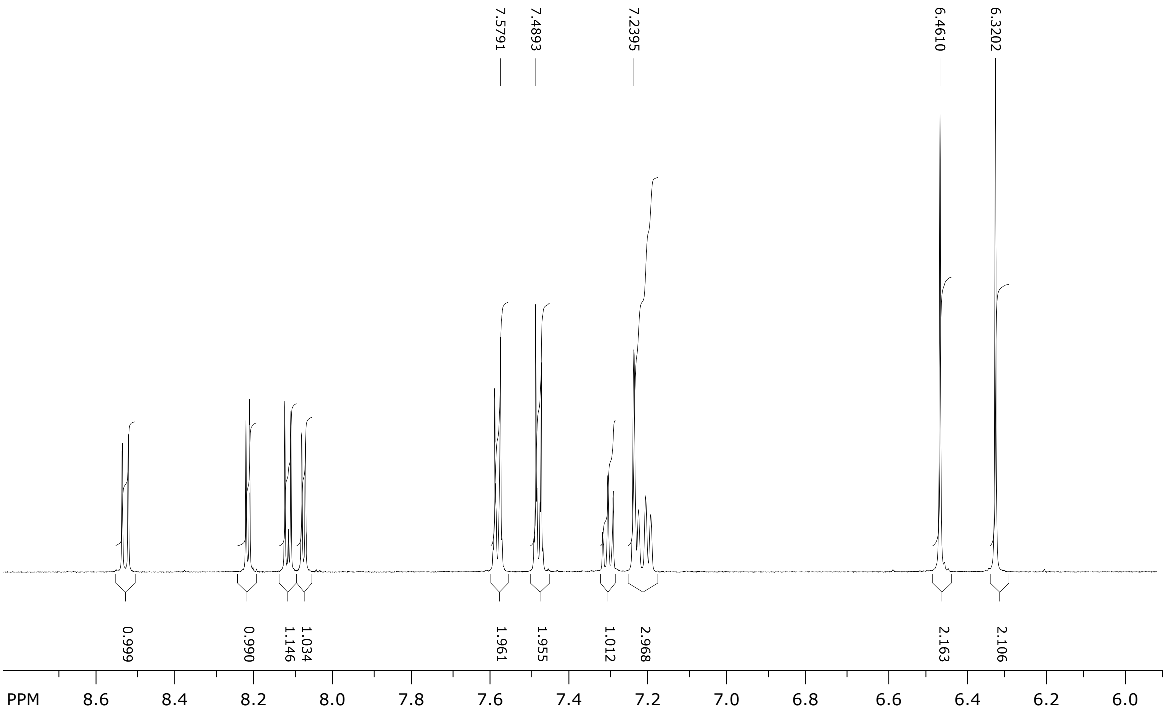

file: ...imljeno do sada za RAD\aj12_1H\fid expt: <zg30>
transmitter freq.: 600.134201 MHz
time domain size: 32768 points
width: 12019.23 Hz = 20.0276 ppm = 0.366798 Hz/pt
number of scans: 64

freq. of 0 ppm: 600.130000 MHz
processed size: 32768 complex points
LB: 0.000 GF: 0.0000
Hz/cm: 70.478 ppm/cm: 0.11744

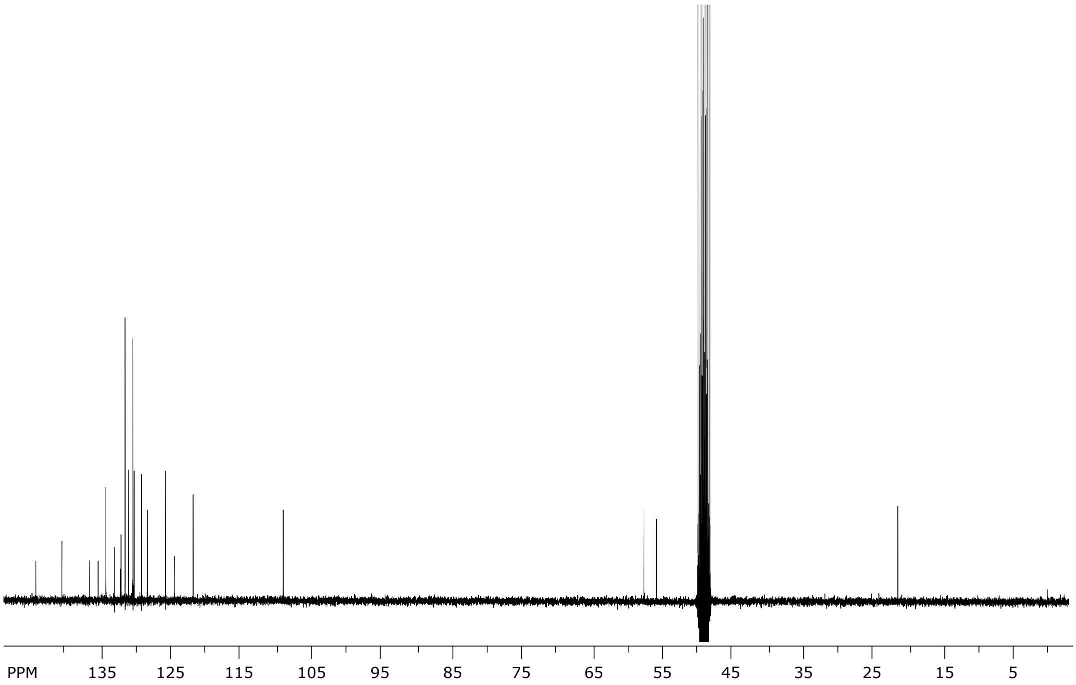

file: ...ada za RAD\jelcic_aj12_av300\2\fid expt: <zgpg30>
transmitter freq.: 75.475295 MHz
time domain size: 32768 points
width: 17985.61 Hz = 238.2980 ppm = 0.548877 Hz/pt
number of scans: 36649

freq. of 0 ppm: 75.467642 MHz
processed size: 32768 complex points
LB: 0.000 GF: 0.0000
Hz/cm: 459.294 ppm/cm: 6.08535

Figure S22. ^{13}C NMR spectrum (CD_3OD) of triazole salt **11**.

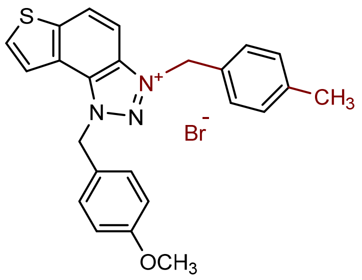

12

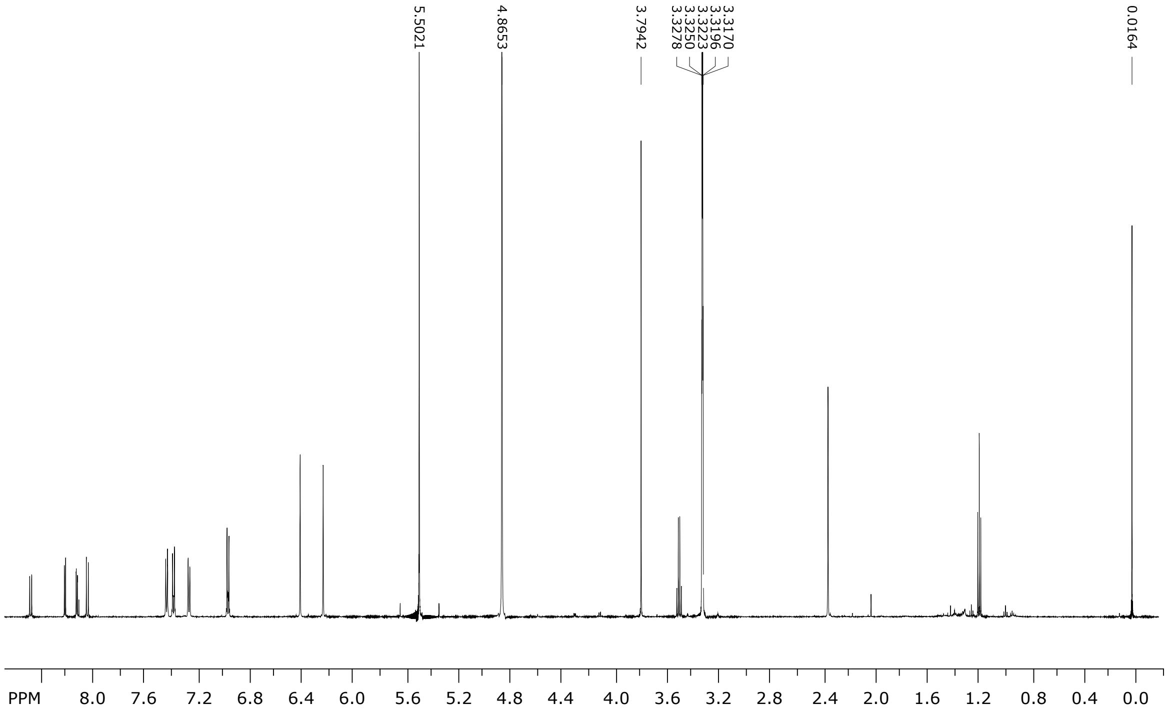

file: ...no do sada za RAD\jelcic_aj7\1\fid expt: <zg30>
transmitter freq.: 600.134201 MHz
time domain size: 32768 points
width: 12626.26 Hz = 21.0391 ppm = 0.385323 Hz/pt
number of scans: 64

freq. of 0 ppm: 600.130000 MHz
processed size: 32768 complex points
LB: 0.000 GF: 0.0000
Hz/cm: 213.291 ppm/cm: 0.35541

Figure S23. ^{1}H NMR spectrum (CD_3OD) of triazole salt **12**.

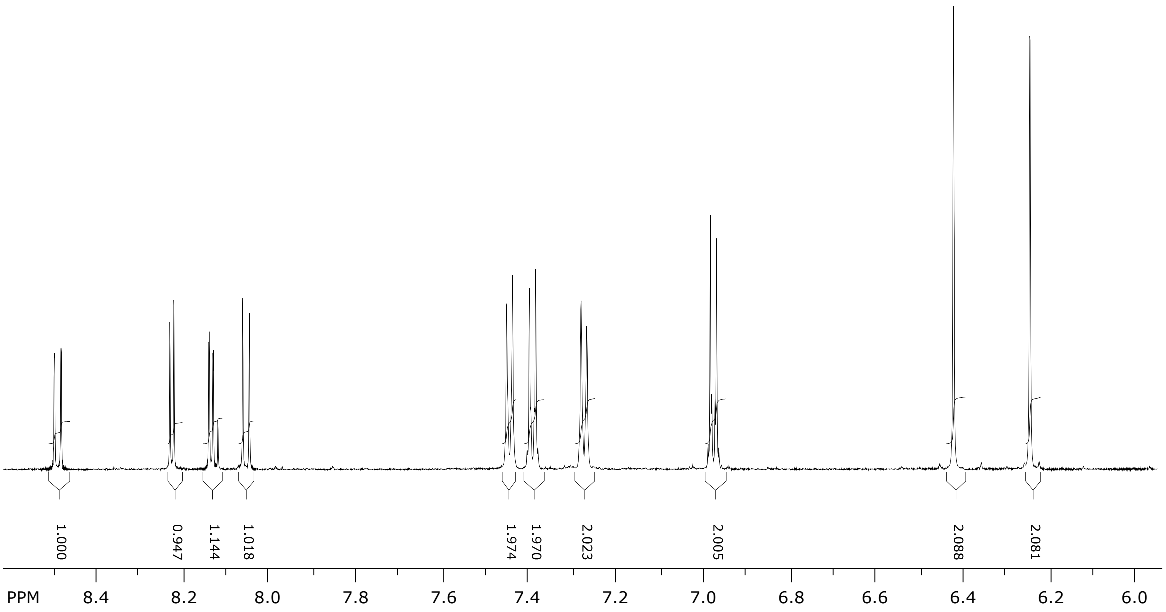

file: ...no do sada za RAD\jelcic_aj7\1\fid expt: <zg30>
transmitter freq.: 600.134201 MHz
time domain size: 32768 points
width: 12626.26 Hz = 21.0391 ppm = 0.385323 Hz/pt
number of scans: 64

freq. of 0 ppm: 600.130000 MHz
processed size: 32768 complex points
LB: 0.000 GF: 0.0000
Hz/cm: 64.168 ppm/cm: 0.10692

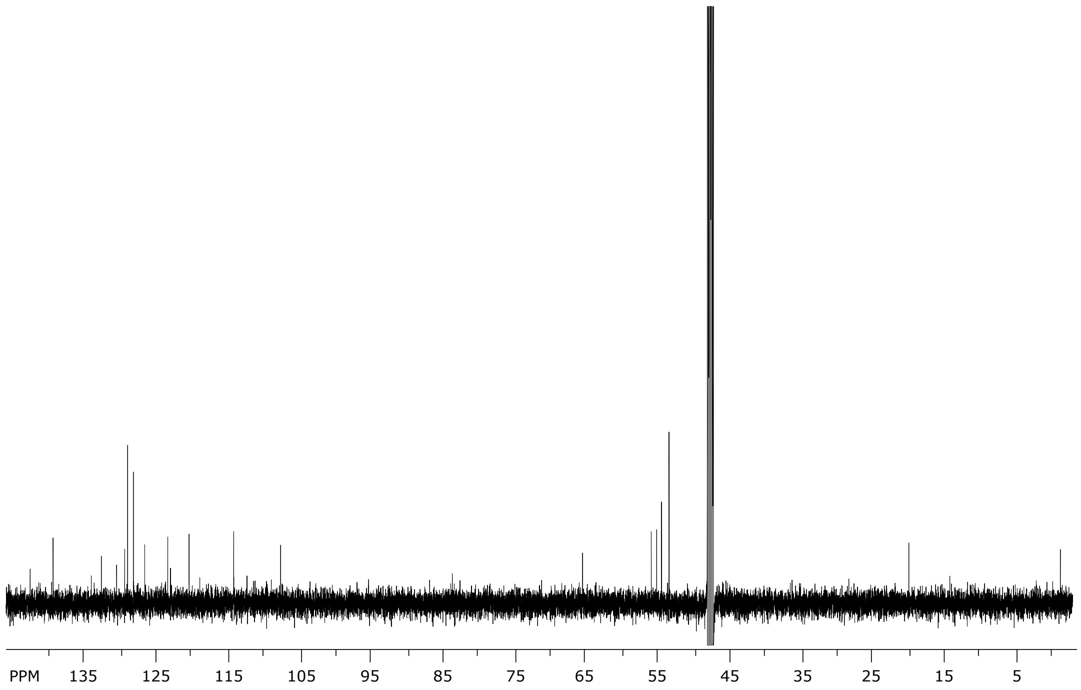

file: ...no do sada za RAD\jelcic_aj7\2\fid expt: <zgpg30>
transmitter freq.: 150.917899 MHz
time domain size: 65536 points
width: 35971.22 Hz = 238.3496 ppm = 0.548877 Hz/pt
number of scans: 2539

freq. of 0 ppm: 150.902809 MHz
processed size: 32768 complex points
LB: 0.000 GF: 0.0000
Hz/cm: 902.329 ppm/cm: 5.97894

Figure S24. ^{13}C NMR spectrum (CD_3OD) of triazole salt **12**.

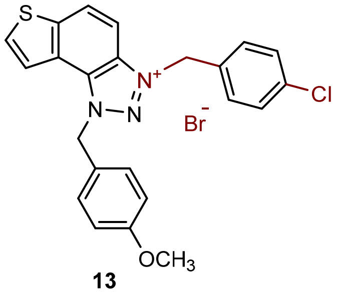

13

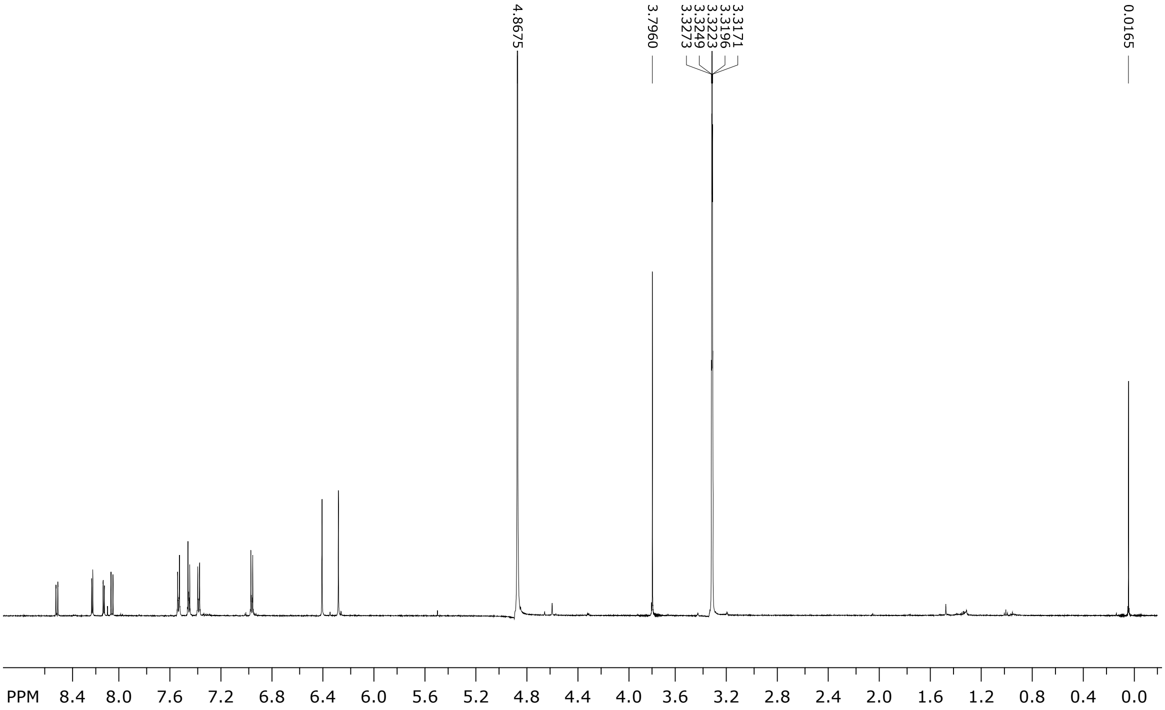

file: ...a za RAD\jelcic_aj8_1H\aj8_13C\fid expt: <zg30>
transmitter freq.: 600.134201 MHz
time domain size: 32768 points
width: 12019.23 Hz = 20.0276 ppm = 0.366798 Hz/pt
number of scans: 64

freq. of 0 ppm: 600.130000 MHz
processed size: 32768 complex points
LB: 0.000 GF: 0.0000
Hz/cm: 220.013 ppm/cm: 0.36661

Figure S25. ^{1}H NMR spectrum (CD_3OD) of triazole salt **13**.

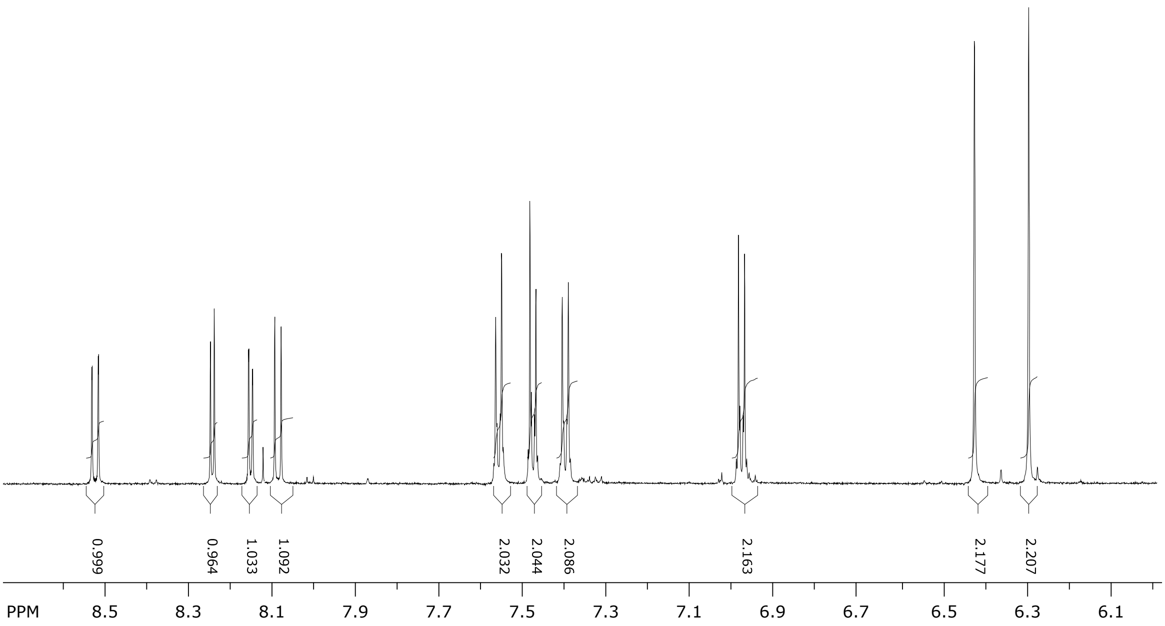

file: ...a za RAD\jelcic_aj8_1H\aj8_13C\fid expt: <zg30>
transmitter freq.: 600.134201 MHz
time domain size: 32768 points
width: 12019.23 Hz = 20.0276 ppm = 0.366798 Hz/pt
number of scans: 64

freq. of 0 ppm: 600.130000 MHz
processed size: 32768 complex points
LB: 0.000 GF: 0.0000
Hz/cm: 66.346 ppm/cm: 0.11055

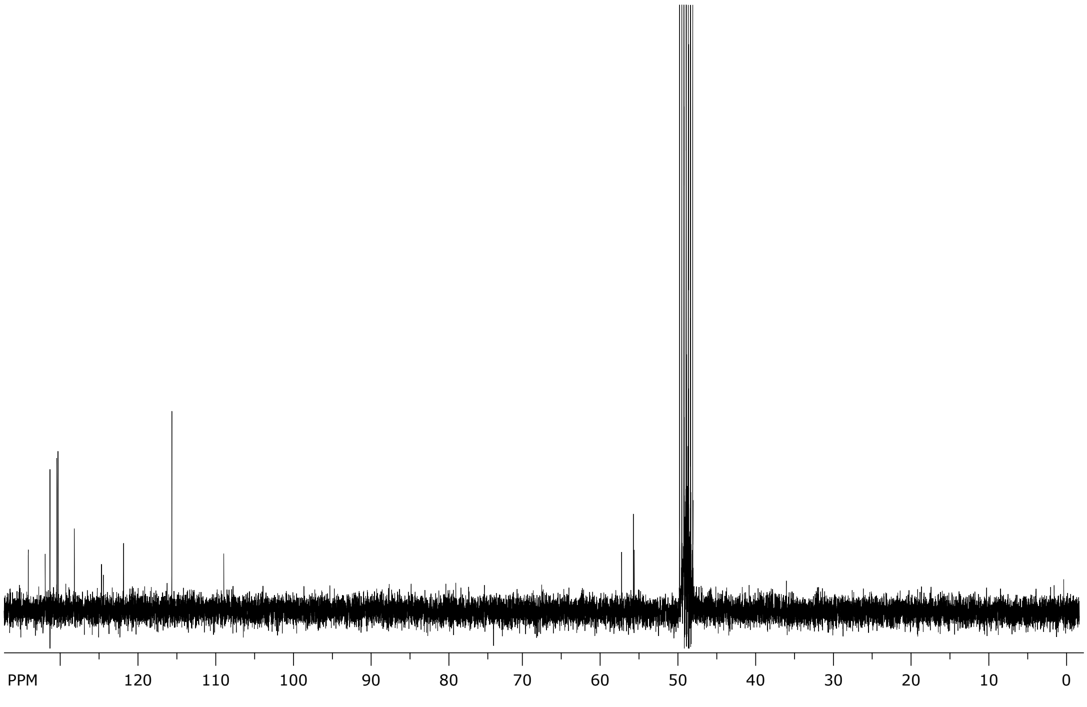

file: ...o sada za RAD\jelcic_aj8_13C\1\fid expt: <zgpg30>
transmitter freq.: 75.475295 MHz
time domain size: 32768 points
width: 17985.61 Hz = 238.2980 ppm = 0.548877 Hz/pt
number of scans: 12800

freq. of 0 ppm: 75.467642 MHz
processed size: 32768 complex points
LB: 0.000 GF: 0.0000
Hz/cm: 421.697 ppm/cm: 5.58721

Figure S26. ^{13}C NMR spectrum (CD_3OD) of triazole salt **13**.

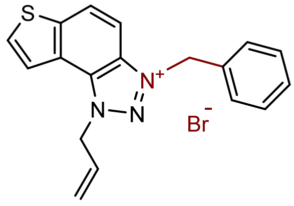

14

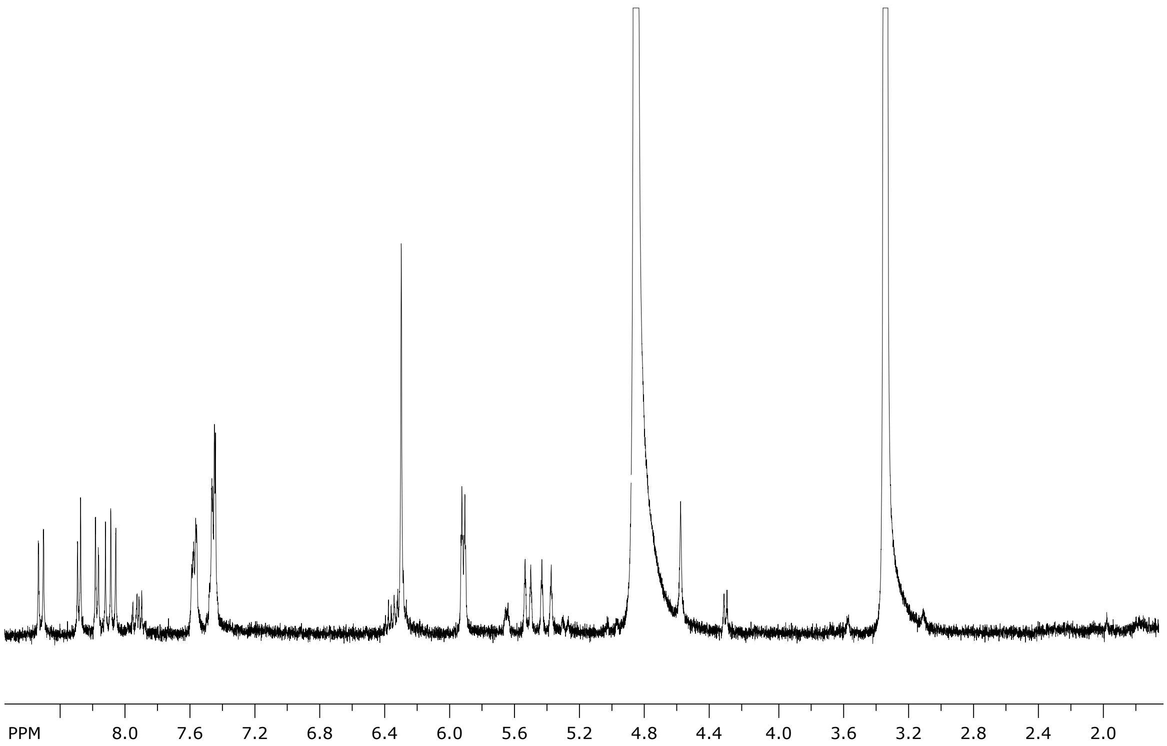

file: ...no do sada za RAD\MR-25-MeOD\1\fid expt: <zg30>
transmitter freq.: 300.132701 MHz
time domain size: 32768 points
width: 6172.84 Hz = 20.5670 ppm = 0.188380 Hz/pt
number of scans: 128

freq. of 0 ppm: 300.130000 MHz
processed size: 32768 complex points
LB: 0.000 GF: 0.0000
Hz/cm: 85.295 ppm/cm: 0.28419

Figure S27. ^{1}H NMR spectrum (CD_3OD) of triazole salt **14**.

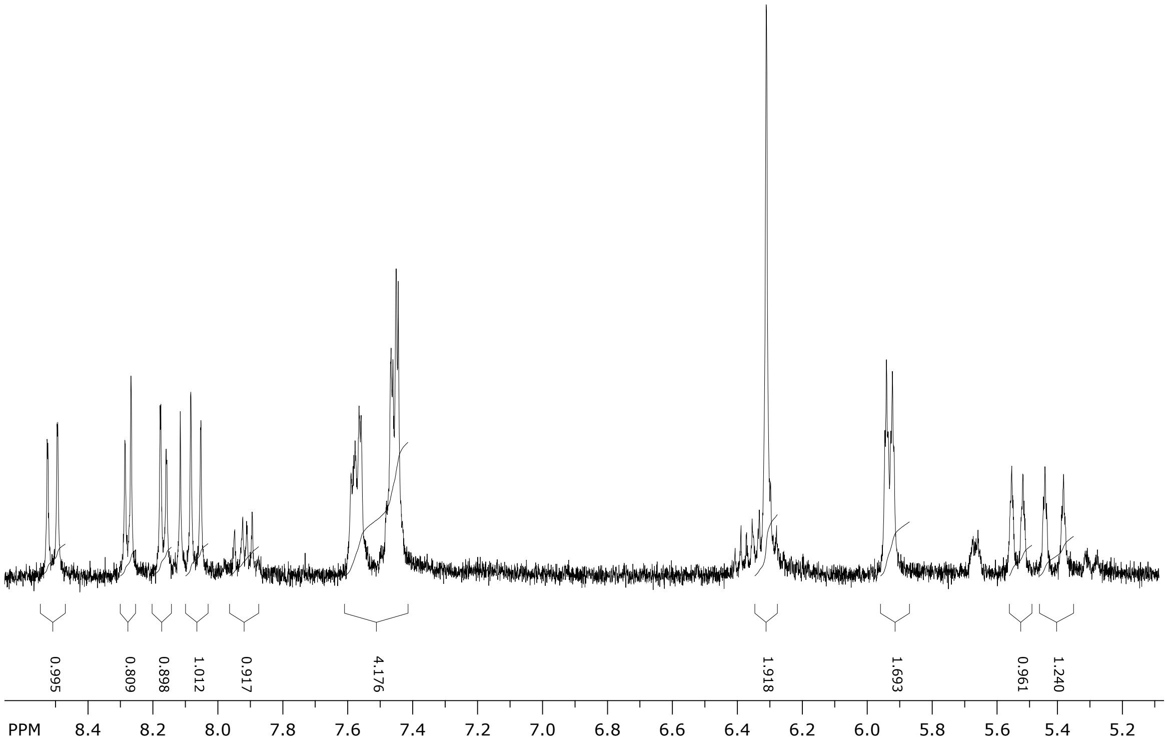

file: ...no do sada za RAD\MR-25-MeOD\1\fid expt: <zg30>
transmitter freq.: 300.132701 MHz
time domain size: 32768 points
width: 6172.84 Hz = 20.5670 ppm = 0.188380 Hz/pt
number of scans: 128

freq. of 0 ppm: 300.130000 MHz
processed size: 32768 complex points
LB: 0.000 GF: 0.0000
Hz/cm: 43.069 ppm/cm: 0.14350

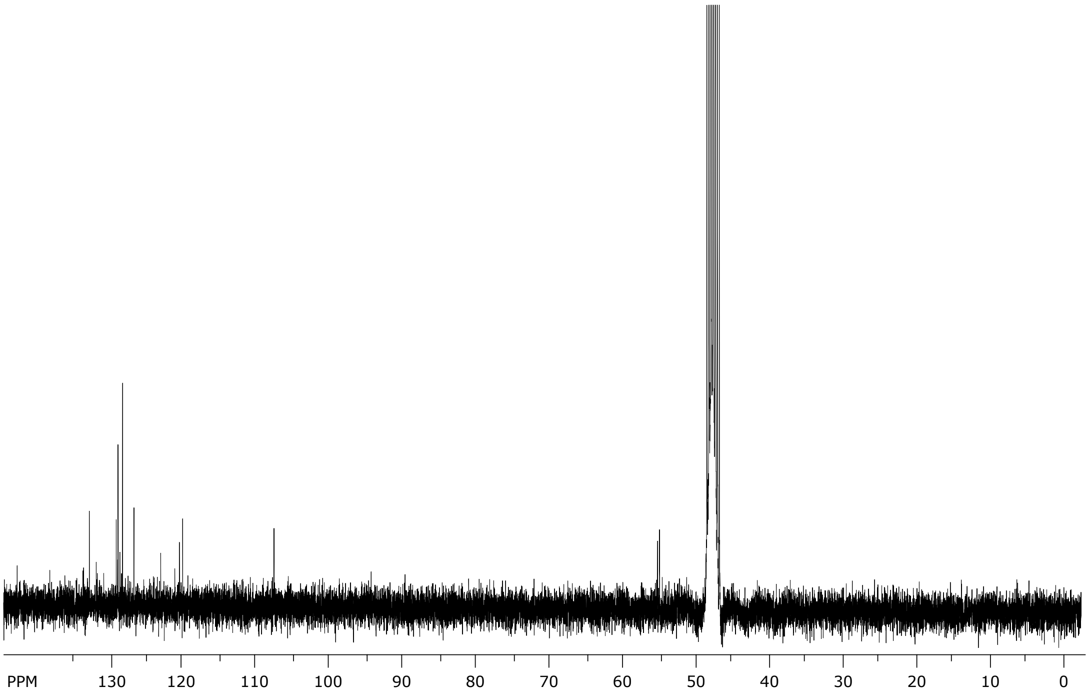

file: ...no do sada za RAD\MR-25-MeOD\2\fid expt: <zgpg30>
transmitter freq.: 75.475295 MHz
time domain size: 65536 points
width: 17985.61 Hz = 238.2980 ppm = 0.274439 Hz/pt
number of scans: 58397

freq. of 0 ppm: 75.467749 MHz
processed size: 32768 complex points
LB: 0.000 GF: 0.0000
Hz/cm: 445.576 ppm/cm: 5.90360

Figure S28. ^{13}C NMR spectrum (CD_3OD) of triazole salt **14**.

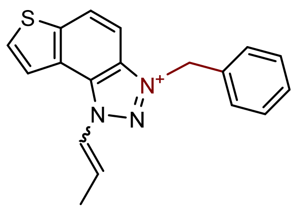

15

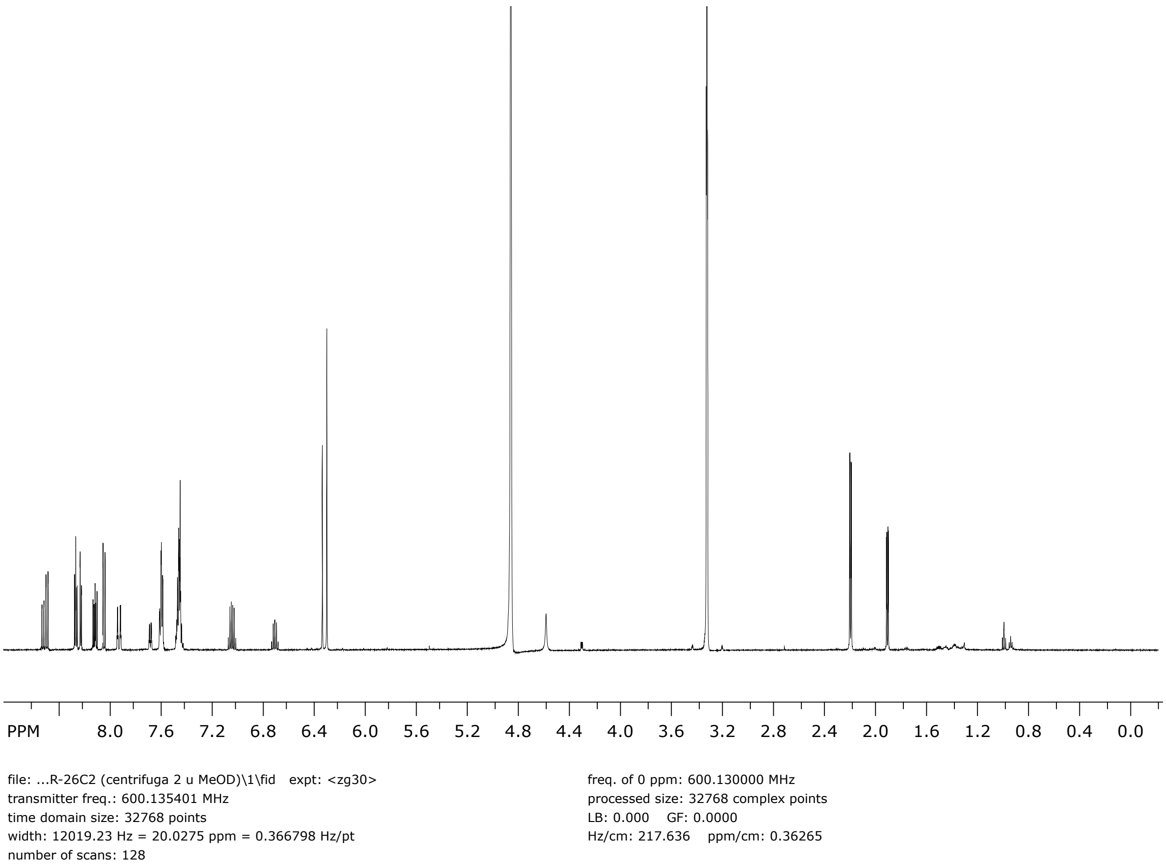

Figure S29. ^{1}H NMR spectrum (CD_3OD) of triazole salt **15** (as mixture of configurational isomers).

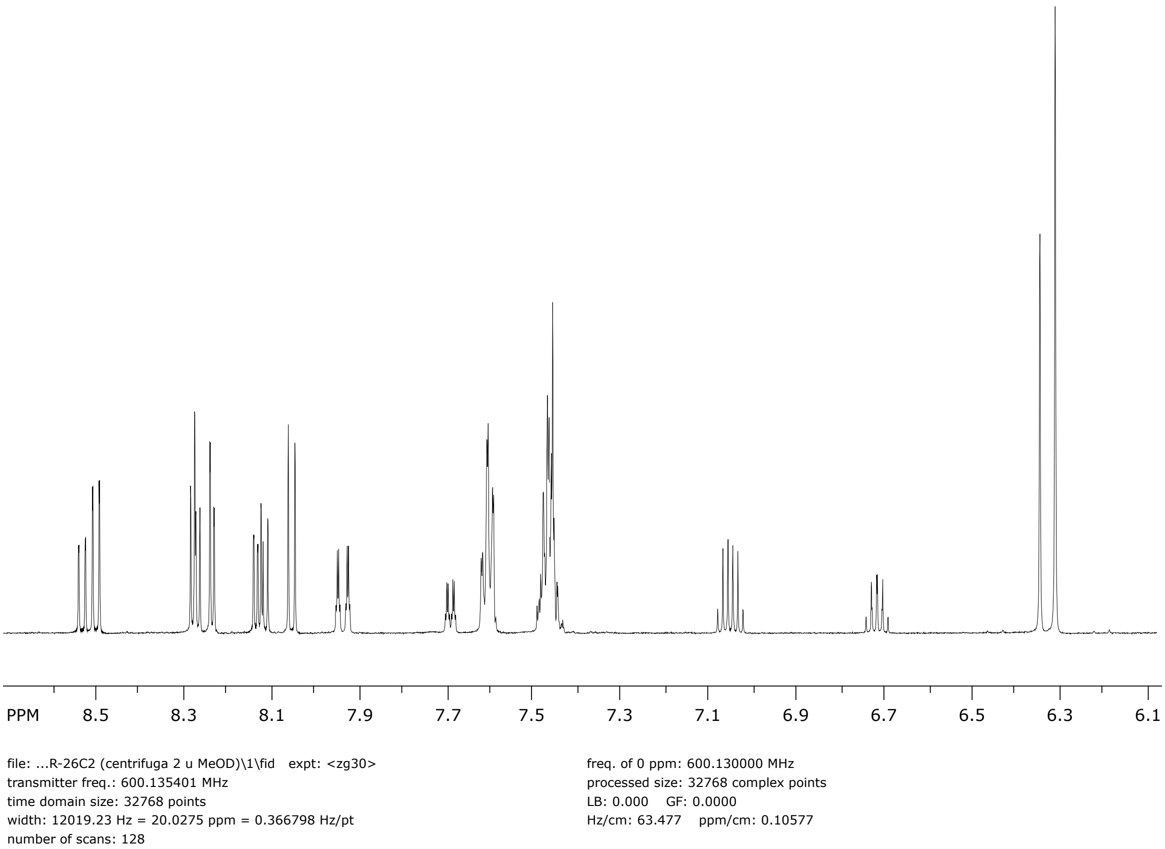

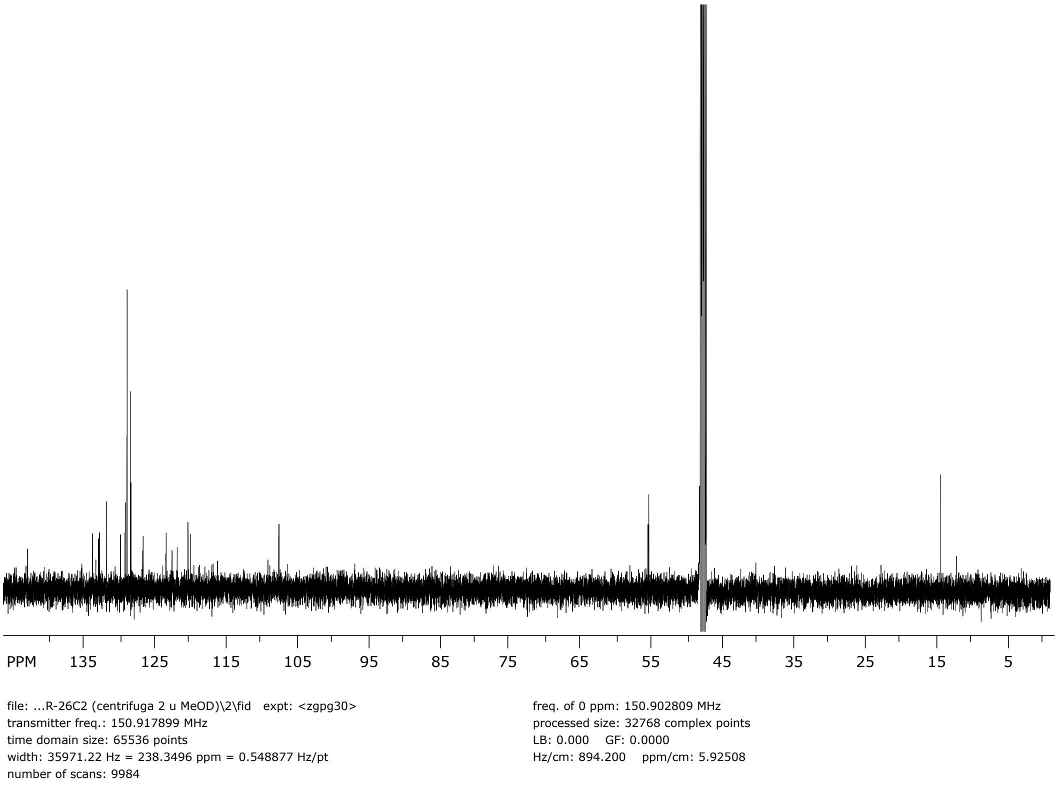

Figure S30. ^{13}C NMR spectrum (CD_3OD) of triazole salt **15** (as mixture of configurational isomers).

2. Mass spectra and HRMS analyses of new charged triazole salts 1–15

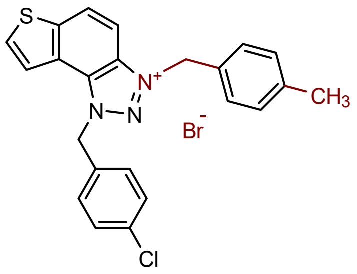

1

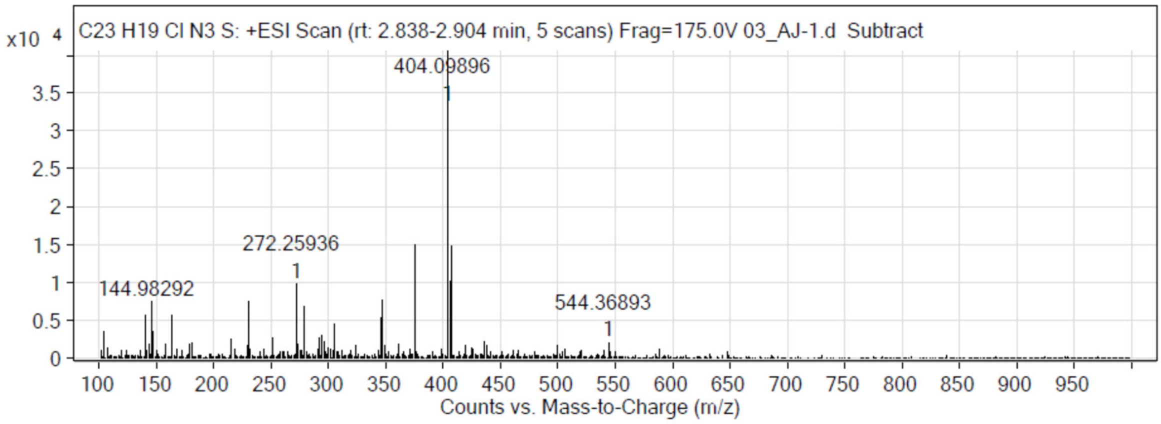

Formula Calculator Results

Formula	Best	Mass	Tgt Mass	Diff (ppm)	Ion Species	Score
C23 H19 Cl N3 S	True	404.09969	404.09882	-2.16	C23 H19 Cl N3 S	97.15

Figure S31. Mass spectrum and HRMS analysis of of triazole salt **1**.

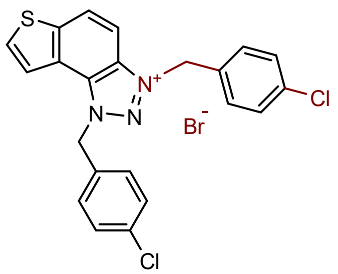

2

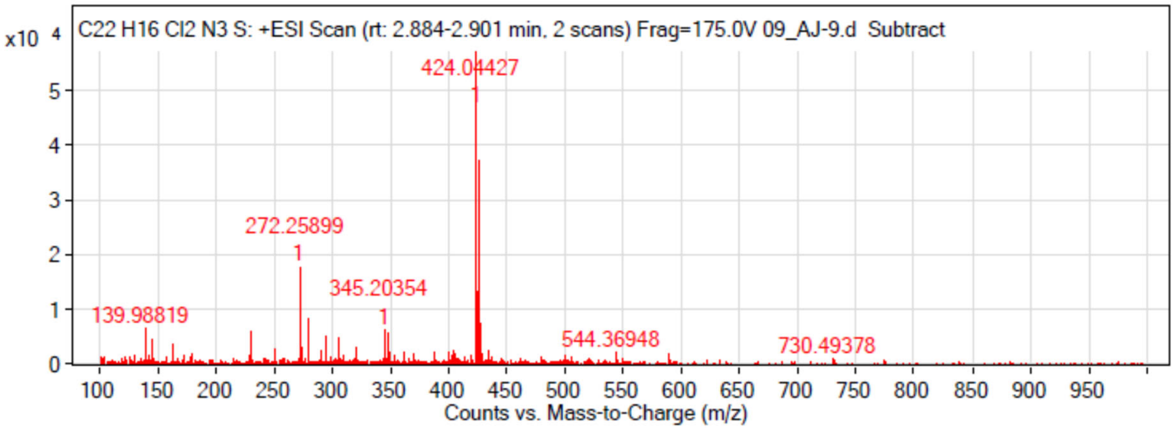

Formula Calculator Results

Formula	Best	Mass	Tgt Mass	Diff (ppm)	Ion Species	Score
C22 H16 Cl2 N3 S	True	424.04477	424.0442	-1.35	C22 H16 Cl2 N3 S	98.15

Figure S32. Mass spectrum and HRMS analysis of of triazole salt **2**.

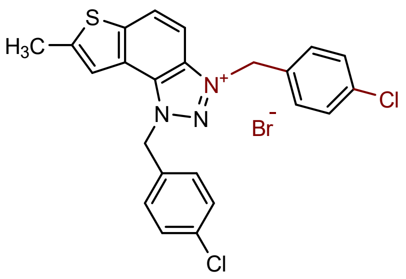

3

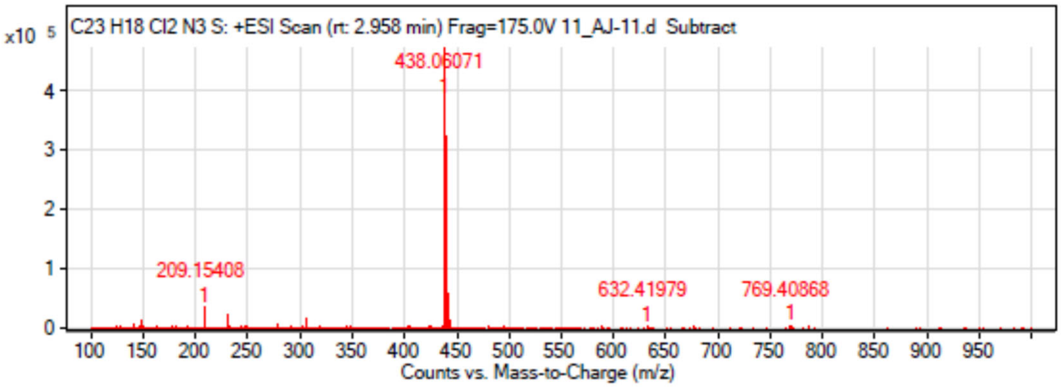

Formula Calculator Results

Formula	Best	Mass	Tgt Mass	Diff (ppm)	Ion Species	Score
C23 H18 Cl2 N3 S	True	438.06103	438.05985	-2.7	C23 H18 Cl2 N3 S	95.31

Figure S33. Mass spectrum and HRMS analysis of of triazole salt **3**.

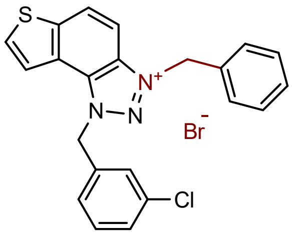

4

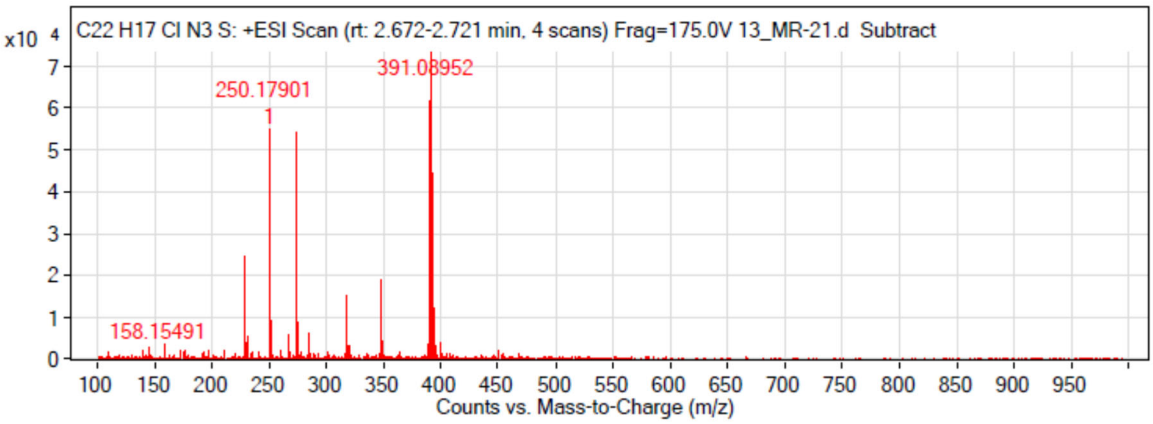

Figure S34. Mass spectrum of triazole salt **4**.

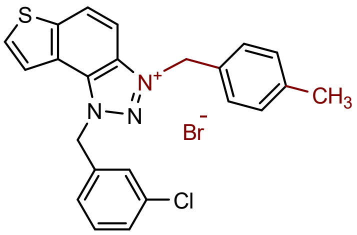

5

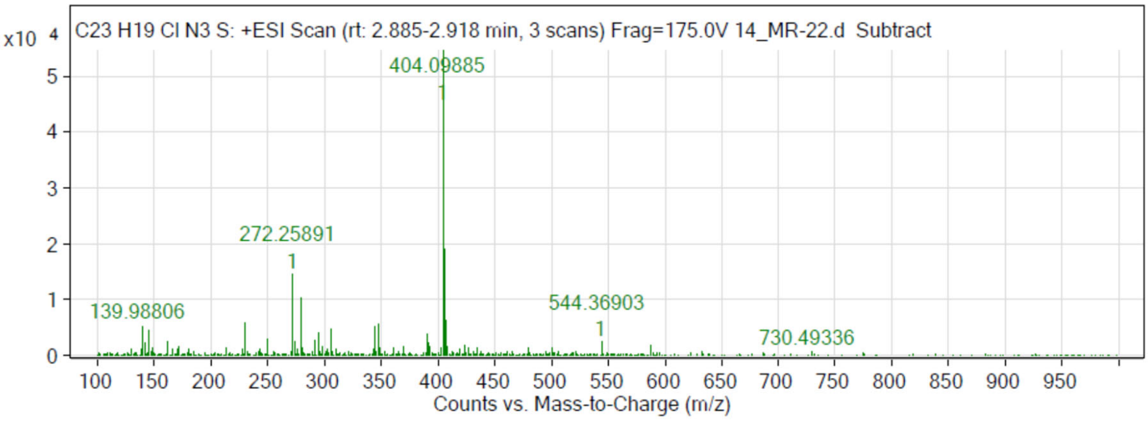

Formula Calculator Results

Formula	Best	Mass	Tgt Mass	Diff (ppm)	Ion Species	Score
C23 H19 Cl N3 S	True	404.09978	404.09882	-2.36	C23 H19 Cl N3 S	90.8

Figure S35. Mass spectrum and HRMS analysis of of triazole salt **5**.

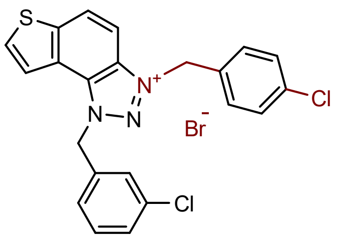

6

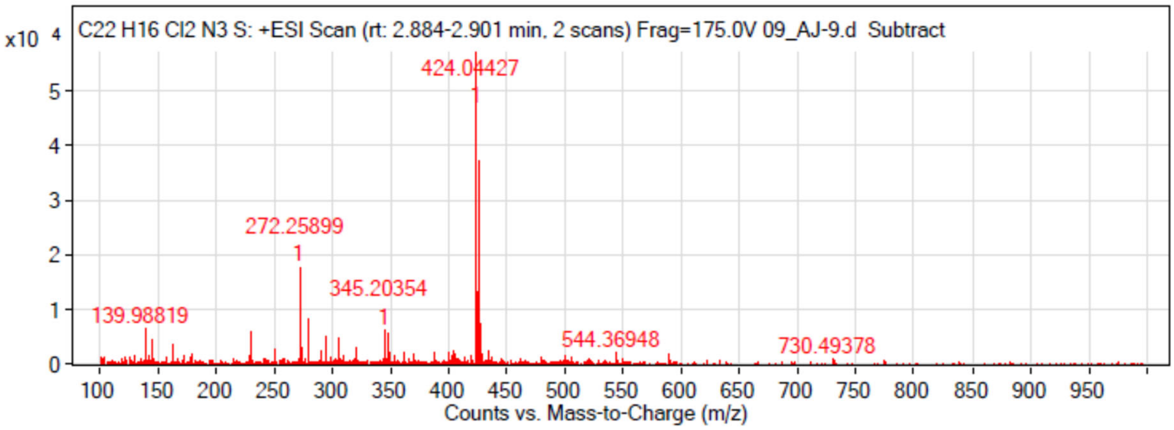

Formula Calculator Results

Formula	Best	Mass	Tgt Mass	Diff (ppm)	Ion Species	Score
C22 H16 Cl2 N3 S	True	424.04477	424.0442	-1.35	C22 H16 Cl2 N3 S	98.15

Figure S36. Mass spectrum and HRMS analysis of of triazole salt **6**.

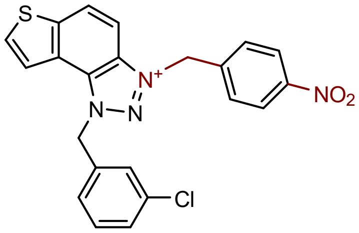

7

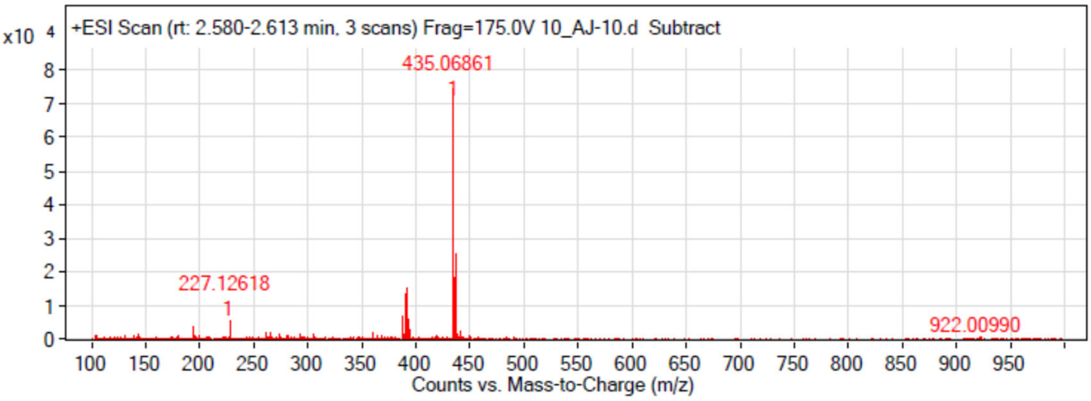

Formula Calculator Results

Formula	Best	Mass	Tgt Mass	Diff (ppm)	Ion Species	Score
C22 H16 Cl N4 O2 S	False	435.06902	435.06825	-1.78	C22 H16 Cl N4 O2 S	95.7

Figure S37. Mass spectrum and HRMS analysis of of triazole salt **7**.

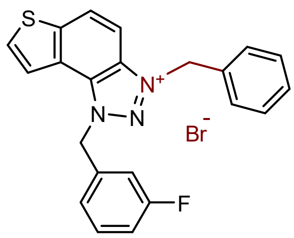

8

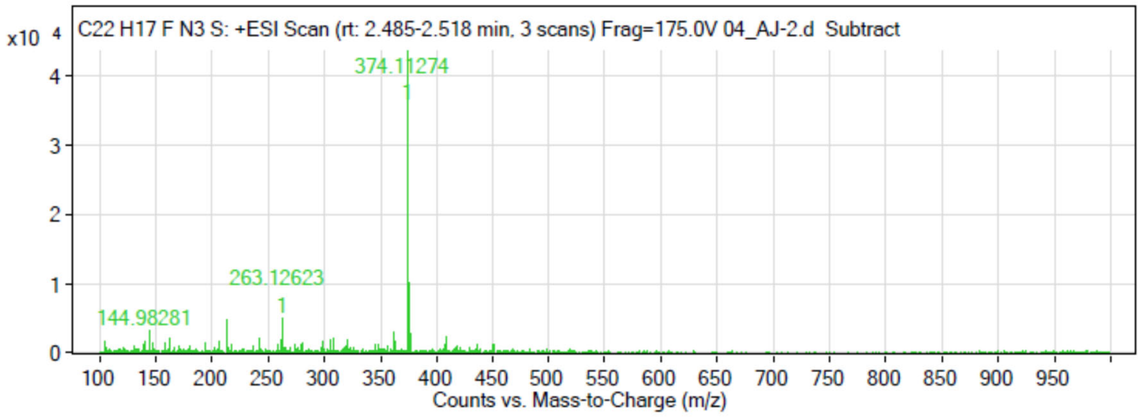

Formula Calculator Results

Formula	Best	Mass	Tgt Mass	Diff (ppm)	Ion Species	Score
C22 H17 F N3 S	True	374.1131	374.11272	-1.02	C22 H17 F N3 S	97.02

Figure S38. Mass spectrum and HRMS analysis of of triazole salt **8**.

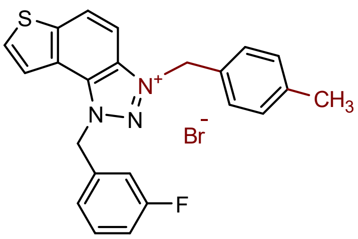

9

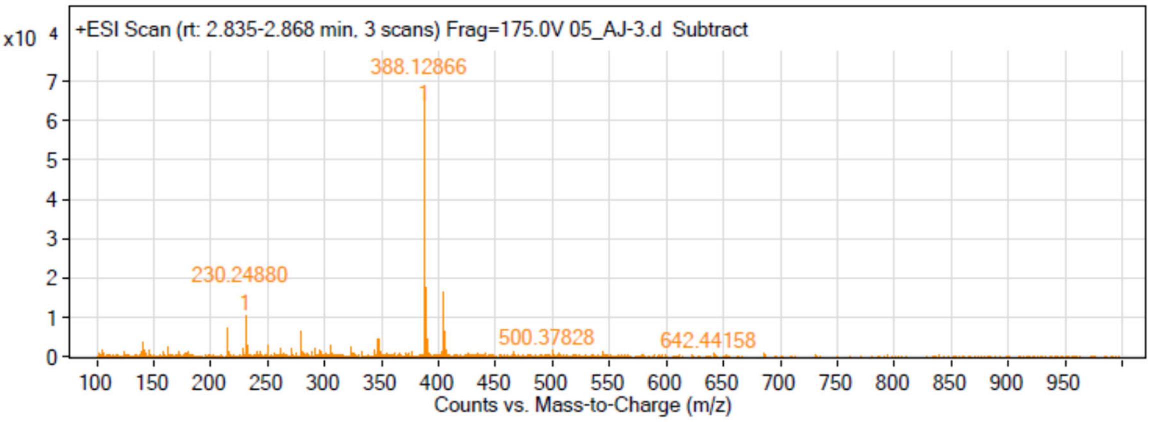

Formula Calculator Results

Formula	Best	Mass	Tgt Mass	Diff (ppm)	Ion Species	Score
C23 H19 F N3 S	False	388.12925	388.12837	-2.26	C23 H19 F N3 S	96.69

Figure S39. Mass spectrum and HRMS analysis of of triazole salt **9**.

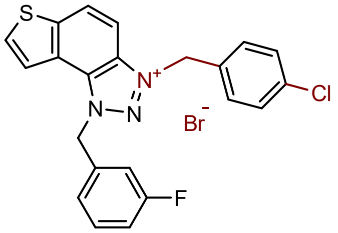

10

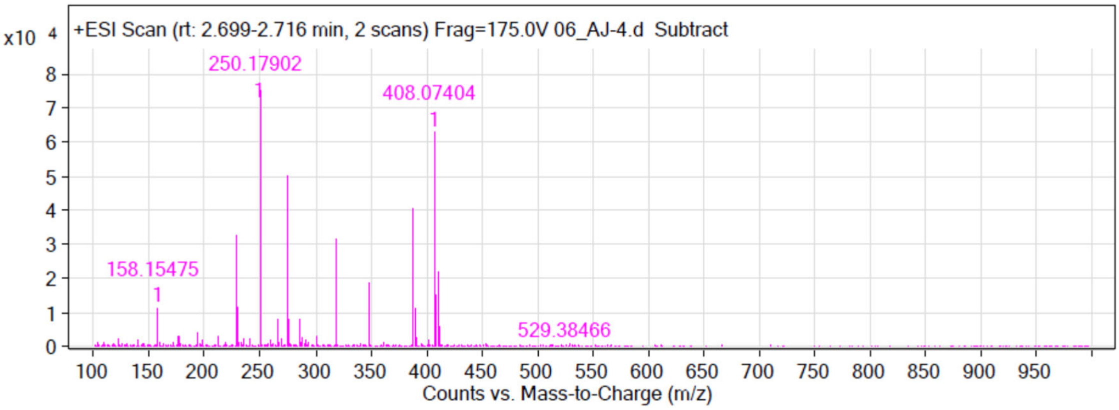

Formula Calculator Results

Formula	Best	Mass	Tgt Mass	Diff (ppm)	Ion Species	Score
C22 H16 Cl F N3 S	True	408.07454	408.07375	-1.93	C22 H16 Cl F N3 S	96.53

Figure S40. Mass spectrum and HRMS analysis of of triazole salt **10**.

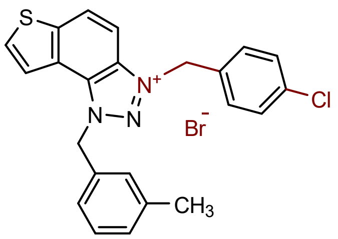

11

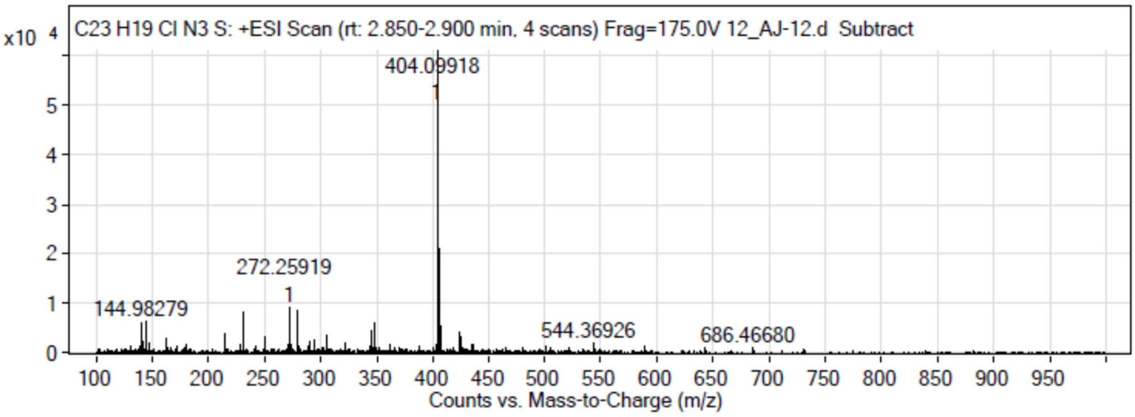

Formula Calculator Results

Formula	Best	Mass	Tgt Mass	Diff (ppm)	Ion Species	Score
C23 H19 Cl N3 S	True	404.0996	404.09882	-1.92	C23 H19 Cl N3 S	96.2

Figure S41. Mass spectrum and HRMS analysis of of triazole salt **11**.

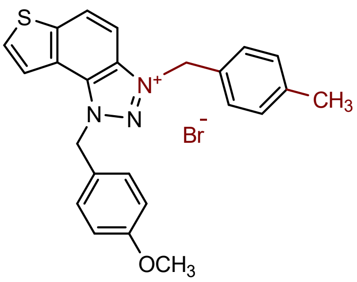

12

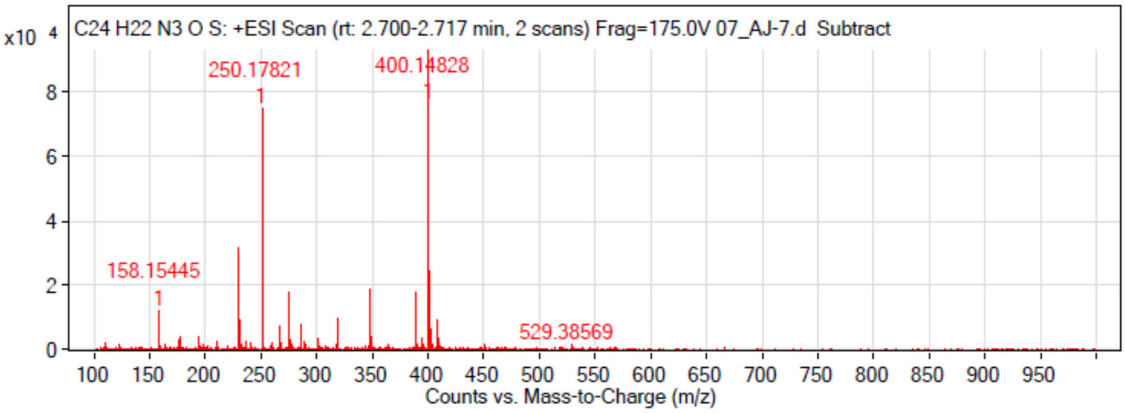

Formula Calculator Results

Formula	Best	Mass	Tgt Mass	Diff (ppm)	Ion Species	Score
C24 H22 N3 O S	True	400.14874	400.14836	-0.96	C24 H22 N3 O S	98.03

Figure S42. Mass spectrum and HRMS analysis of of triazole salt **12**.

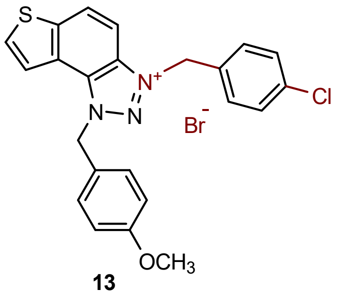

13

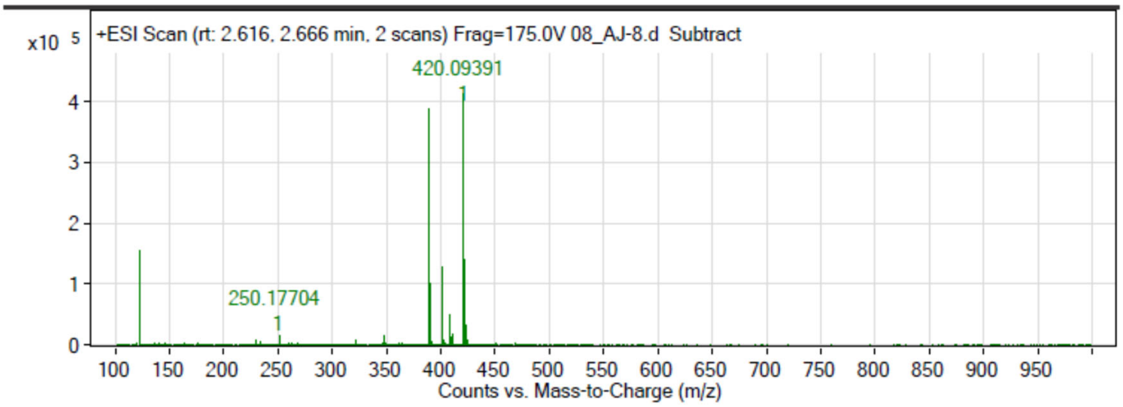

Formula Calculator Results

Formula	Best	Mass	Tgt Mass	Diff (ppm)	Ion Species	Score
C23 H19 Cl N3 O S	True	420.09431	420.09374	-1.36	C23 H19 Cl N3 O S	96.53

Figure S43. Mass spectrum and HRMS analysis of of triazole salt **13**.

3. Cartesian coordinates of ligands 9 and 11 docked into the active site of AChE

Cartesian coordinates of salt 11 docked into the active site of AChE

N	-16.197	-42.926	28.947
C	-15.426	-43.577	28.012
C	-14.553	-44.384	28.766
N	-14.889	-44.140	30.077
N	-15.871	-43.271	30.182
C	-13.567	-45.209	28.186
H	-12.902	-45.839	28.800
C	-13.479	-45.186	26.810
H	-12.720	-45.805	26.302
C	-14.352	-44.377	26.042
C	-15.362	-43.551	26.597
S	-14.352	-44.263	24.303
C	-15.678	-43.142	24.339
H	-16.125	-42.705	23.430
C	-16.113	-42.854	25.599
H	-16.945	-42.165	25.822
C	-17.281	-41.938	28.771
H	-16.921	-41.059	28.188
H	-17.517	-41.436	29.738
C	-18.536	-42.517	28.148
C	-19.230	-43.559	28.771
H	-18.849	-44.008	29.704
C	-20.408	-44.018	28.194
F	-21.065	-45.025	28.793
C	-20.926	-43.469	27.025
H	-21.864	-43.851	26.589
C	-20.231	-42.425	26.419
H	-20.626	-41.968	25.496
C	-19.038	-41.952	26.971
H	-18.491	-41.131	26.478
C	-14.294	-44.705	31.317
H	-14.864	-45.599	31.661
H	-14.471	-44.030	32.187
C	-12.825	-45.025	31.172
C	-12.381	-46.354	31.135
H	-13.111	-47.179	31.195
C	-11.024	-46.637	31.023
H	-10.692	-47.688	30.985
C	-10.069	-45.610	30.957
C	-10.523	-44.286	31.000
H	-9.793	-43.460	30.949
C	-8.597	-45.931	30.867
H	-7.839	-45.115	30.815
H	-8.441	-46.608	29.995
H	-8.333	-46.597	31.721
C	-11.883	-43.994	31.106
H	-12.217	-42.943	31.138

Cartesian coordinates of triazolium salt 11 docked into the active site of AChE

C	-11.893	-44.070	31.486
H	-12.222	-43.057	31.773
C	-12.848	-45.053	31.206
C	-12.421	-46.339	30.851
H	-13.167	-47.123	30.639
C	-11.064	-46.637	30.763
H	-10.733	-47.650	30.478
C	-10.125	-45.639	31.040
C	-10.531	-44.356	31.407
H	-9.783	-43.577	31.632
l	-8.423	-46.009	30.931
C	-14.323	-44.737	31.324
H	-14.899	-45.638	31.639
H	-14.520	-44.078	32.202
N	-14.890	-44.152	30.084
C	-14.558	-44.410	28.775
C	-13.602	-45.272	28.199
H	-12.965	-45.927	28.816
C	-15.396	-43.569	28.017

C	-13.506	-45.253	26.824
H	-12.770	-45.901	26.319
C	-14.344	-44.409	26.052
S	-14.330	-44.294	24.313
C	-15.323	-43.546	26.603
C	-15.612	-43.123	24.343
H	-16.037	-42.668	23.432
C	-16.043	-42.820	25.602
H	-16.850	-42.101	25.822
N	-16.145	-42.888	28.950
N	-15.838	-43.243	30.184
C	-17.181	-41.846	28.770
H	-17.436	-41.372	29.746
H	-16.761	-40.961	28.237
C	-18.432	-42.344	28.075
C	-19.202	-43.333	28.701
H	-18.915	-43.684	29.707
C	-20.327	-43.885	28.078
C	-20.677	-43.407	26.806
H	-21.566	-43.816	26.297
C	-21.137	-44.969	28.747
H	-22.033	-45.409	28.251
H	-21.446	-44.599	29.752
H	-20.446	-45.802	29.013
C	-19.918	-42.423	26.176
H	-20.211	-42.062	25.176
C	-18.790	-41.892	26.802
H	-18.185	-41.121	26.296

4. Cartesian coordinates of ligands 9 and 11 docked into the active site of BChE

Cartesian coordinates of salt 9 docked into the active site of BChE

N	134.315	112.882	42.978
C	133.204	113.693	42.958
C	133.606	114.835	42.240
N	134.915	114.595	41.898
N	135.335	113.429	42.338
C	132.760	115.940	42.007
H	133.098	116.820	41.434
C	131.488	115.862	42.534
H	130.785	116.700	42.389
C	131.074	114.718	43.259
C	131.897	113.587	43.495
S	129.499	114.490	43.969
C	129.948	112.917	44.553
H	129.265	112.266	45.124
C	131.227	112.564	44.237
H	131.690	111.604	44.520
C	134.517	111.553	43.591
H	133.589	111.212	44.106
H	135.202	111.624	44.468
C	135.005	110.501	42.615
C	135.951	109.547	43.003
H	136.393	109.573	44.013
C	136.324	108.565	42.093
F	137.239	107.658	42.470
C	135.781	108.493	40.814
H	136.095	107.703	40.111
C	134.832	109.442	40.442
H	134.381	109.399	39.436
C	134.447	110.448	41.333
H	133.702	111.201	41.025
C	135.860	115.460	41.141
H	135.606	116.538	41.273
H	135.693	115.367	40.043
C	137.305	115.199	41.491
C	137.995	116.046	42.369
H	137.475	116.905	42.825
C	139.332	115.809	42.668
H	139.857	116.480	43.368
C	140.026	114.731	42.095
C	139.330	113.894	41.214
H	139.852	113.041	40.749
C	141.486	114.503	42.403
H	142.037	113.647	41.948
H	141.603	114.443	43.510
H	142.042	115.440	42.167
C	137.987	114.122	40.915
H	137.459	113.448	40.219

Cartesian coordinates of salt 11 docked into the active site of BChE

C	132.678	111.928	41.487
H	132.431	111.953	40.412
C	134.019	111.931	41.889
C	134.322	111.893	43.256
H	135.376	111.885	43.581
C	133.307	111.865	44.208
H	133.551	111.841	45.283
C	131.974	111.868	43.786
C	131.651	111.894	42.430
H	130.596	111.888	42.107
Cl	130.698	111.834	44.977
C	135.123	111.938	40.855
H	134.720	111.713	39.840
H	135.795	111.057	40.983
N	135.901	113.201	40.848
C	137.209	113.413	41.216
C	138.200	112.542	41.711
H	138.011	111.463	41.843
C	137.418	114.793	41.025
C	139.418	113.108	42.022
H	140.228	112.475	42.421

C	139.639	114.495	41.834
S	141.127	115.334	42.179
C	138.667	115.391	41.324
C	140.448	116.838	41.641
H	141.008	117.788	41.641
C	139.156	116.732	41.218
H	138.559	117.579	40.839
N	136.212	115.270	40.564
N	135.311	114.312	40.459
C	135.801	116.647	40.208
H	136.598	117.378	40.481
H	135.795	116.782	39.101
C	134.471	117.060	40.803
C	133.836	116.193	41.702
H	134.343	115.260	42.001
C	132.573	116.485	42.229
C	131.961	117.687	41.844
H	130.971	117.948	42.254
C	131.883	115.533	43.176
H	132.369	114.578	43.482
H	130.879	115.292	42.755
H	131.614	116.097	44.100
C	132.585	118.557	40.952
H	132.086	119.497	40.663
C	133.837	118.245	40.422
H	134.323	118.930	39.707

5. Free energies of binding, the number of conformational clusters, and distribution of conformations obtained by molecular docking

Table S1. Free energies of binding, ΔG_{bind}, obtained by molecular docking of ligands **9**, **11**, and reference ligand donepezil into the active site of AChE (4EY7.pdb), along with the number of conformational clusters and distribution of conformations.

Ligand	ΔG_{bind}/kcal mol^{-1}		Number of distinctive conformational clusters	Distribution of conformations within clusters with $n > 1$ (n = cluster population)
	lowest	highest		
**9**	−8.46	−8.04	4	11, 8, 2, 2
**11**	−8.34	−8.28	3	12, 9, 2
Donepezil	−8.80	−8.23	1	25

Table S2. Free energies of binding, , ΔG_{bind}, obtained by molecular docking of ligands **9**, **11**, and reference ligand donepezil into the active site of BChE (1P0I.pdb), along with the number of conformational clusters and distribution of conformations.

Ligand	ΔG_{bind}/kcal mol^{-1}		Number of distinctive conformational clusters	Distribution of conformations within clusters with $n > 1$ (n = cluster population)
	lowest	highest		
**9**	−7.32	−6.63	4	7, 4, 3, 2
**11**	−7.42	−7.31	2	18, 7
Donepezil	−7.80	−6.23	6	8, 4, 5, 2, 2, 3

Table S3. The mutagenic potential of new charged thienobenzo-1,2,3-triazolinium salts **1–15** through Lhasa M7 evaluation (green square—negative, red square—positive, white square—no data available); grey highlight—negative, yellow highlight—positive, white—strongly negative.

Structure	ICH M7 Class	Derek Prediction	Sarah Prediction	Experimental Data	Overall In Silico
**1**	Class 5	[−][−][−][ ]	[−][ ][ ][ ]	Carc: Unspecified<br>Ames: Unspecified	Negative
**2**	Class 5	[−][−][−][ ]	[−][ ][ ][ ]	Carc: Unspecified<br>Ames: Unspecified	Negative
**3**	Class 5	[−][−][−][ ]	[−][ ][ ][ ]	Carc: Unspecified<br>Ames: Unspecified	Negative
**4**	Class 5	[−][−][−][ ]	[−][ ][ ][ ]	Carc: Unspecified<br>Ames: Unspecified	Negative
**5**	Class 3	[−][−][−][ ]	[+][ ][ ][ ]	Carc: Unspecified<br>Ames: Unspecified	Positive
**6**	Inconclusive	[−][−][−][ ]	[ ][ ][ ][ ]	Carc: Unspecified<br>Ames: Unspecified	Negative
**7**	Class 3	[+][+][+][ ]	[+][ ][ ][ ]	Carc: Unspecified<br>Ames: Unspecified	Positive
**8**	Class 5	[−][−][−][ ]	[−][ ][ ][ ]	Carc: Unspecified<br>Ames: Unspecified	Negative
**9**	Class 5	[−][−][−][ ]	[−][ ][ ][ ]	Carc: Unspecified<br>Ames: Unspecified	Negative
**10**	Class 5	[−][−][−][ ]	[−][ ][ ][ ]	Carc: Unspecified<br>Ames: Unspecified	Negative
**11**	Inconclusive	[−][−][−][ ]	[ ][ ][ ][ ]	Carc: Unspecified<br>Ames: Unspecified	Negative
**12**	Class 3	[−][−][−][ ]	[+][ ][ ][ ]	Carc: Unspecified<br>Ames: Unspecified	Positive
**13**	Class 3	[−][−][−][ ]	[+][ ][ ][ ]	Carc: Unspecified<br>Ames: Unspecified	Positive
**14**	Inconclusive	[−][−][−][ ]	[ ][ ][ ][ ]	Carc: Unspecified<br>Ames: Unspecified	Negative
**15**	Inconclusive	[−][−][−][ ]	[ ][ ][ ][ ]	Carc: Unspecified<br>Ames: Unspecified	Negative
